# Genomewide Identification of Essential Genes and Fitness Determinants of *Streptococcus mutans* UA159

**DOI:** 10.1128/mSphere.00031-18

**Published:** 2018-02-07

**Authors:** Robert C. Shields, Lin Zeng, David J. Culp, Robert A. Burne

**Affiliations:** a Department of Oral Biology, College of Dentistry, University of Florida, Gainesville, Florida, USA; University of Kentucky

**Keywords:** *Streptococcus*, antimicrobial targets, dental caries, essential genome, transposon sequencing

## Abstract

Transposon mutagenesis coupled with next-generation DNA sequencing (Tn-seq) is a powerful tool for discovering regions of the genome that are required for the survival of bacteria in different environments. We adapted this technique to the dental caries pathogen *Streptococcus mutans* UA159 and identified 11% of the genome as essential, with many genes encoding products required for replication, translation, lipid metabolism, and cell wall biogenesis. Comparison of the essential genome of *S. mutans* UA159 with those of selected other streptococci for which such information is available revealed several metabolic pathways and genes that are required in *S. mutans*, but not in some *Streptococcus* spp. We further identified genes that are essential for sustained growth in rich or defined medium, as well as for persistence *in vivo* in a rodent model of oral infection. Collectively, our results provide a novel and comprehensive view of the genes required for essential processes of *S. mutans*, many of which could represent potential targets for therapeutics.

**IMPORTANCE** Tooth decay (dental caries) is a common cause of pain, impaired quality of life, and tooth loss in children and adults. It begins because of a compositional change in the microorganisms that colonize the tooth surface driven by repeated and sustained carbohydrate intake. Although several bacterial species are associated with tooth decay, *Streptococcus mutans* is the most common cause. Therefore, it is important to identify biological processes that contribute to the survival of *S. mutans* in the human mouth, with the aim of disrupting the processes with antimicrobial agents. We successfully applied Tn-seq to *S. mutans*, discovering genes that are required for survival, growth, and persistence, both in laboratory environments and in a mouse model of tooth decay. This work highlights new avenues for the control of an important human pathogen.

## Abstract (precis)

Tooth decay (dental caries) is a common cause of pain, impaired quality of life, and tooth loss in children and adults. It begins because of a compositional change in the microorganisms that colonize the tooth surface driven by repeated and sustained carbohydrate intake. Although several bacterial species are associated with tooth decay, *Streptococcus mutans* is the most common cause. Therefore, it is important to identify biological processes that contribute to the survival of *S. mutans* in the human mouth, with the aim of disrupting the processes with antimicrobial agents. We successfully applied Tn-seq to *S. mutans*, discovering genes that are required for survival, growth, and persistence, both in laboratory environments and in a mouse model of tooth decay. This work highlights new avenues for the control of an important human pathogen.

## INTRODUCTION

Dental caries is one of the most prevalent biofilm-related infectious diseases, with approximately 2.4 billion adults and 621 million children living with untreated caries as recently as 2010 (1). A polymicrobial disease, caries is associated with the opportunistic pathogen *Streptococcus mutans* (2, 3), although the strength of the association is influenced by geographic and socioeconomic factors (4). Aspects of the physiology of *S. mutans* that are thought to explain its substantial cariogenic potential include its capacity to form biofilms on teeth, to produce significant quantities of organic acids from many different dietary carbohydrates, to grow and metabolize at low pH, and to adapt rapidly to fluctuations in oxygen tension, pH, carbohydrate source and availability, and other environmental inputs (5, 6). In recent years, Cnm-positive strains of *S. mutans*, Cnm being a collagen-binding protein, have been associated with nonoral diseases, including infective endocarditis and cerebral microbleeds (7, 8). Despite extensive research on the many virulence-associated attributes of *S. mutans*, more comprehensive approaches are needed to develop a complete understanding of the biology of this microorganism, with the aim of developing highly effective prevention and treatment strategies.

Essential gene screens have provided a wealth of information for several important bacterial species (9–14). In these studies, mutant libraries, designed to include all the nonessential genes of a microorganism, are screened across diverse environments, and the genes that contribute to survival in particular conditions are identified. Genes that cannot be mutagenized are also discovered, defining the essential elements in the genome that are required for survival. Earlier work by Quivey et al. (15) made an important contribution to mutant screens in *S. mutans* by creating a gene-specific barcode library in *S. mutans* UA159. However, coverage of the genome was relatively low, as deletion constructs were not created for 530 genes (*S. mutans* UA159 has 1,960 protein-coding genes). Recently, transposon mutagenesis has been combined with next-generation DNA sequencing (11, 16). This technique, known as Tn-seq (or TraDIS), measures changes in the relative abundance of thousands of transposon mutants simultaneously as they are grown under one or more experimental conditions. Transposon insertions that become under- or overrepresented in the population indicate genes and pathways that are important or potentially dispensable, respectively, in specific conditions. Tn-seq technologies have been applied in *Streptococcus pneumoniae* (11), *Streptococcus pyogenes* (17), and *Streptococcus agalactiae* (18). It is only recently that this approach has been applied to the study of *S. mutans* (19), with a focus on genetic competence.

In the current study, we created high-coverage transposon libraries and grew them in *in vitro* and *in vivo* environments. After sequencing, analysis of transposon insertion sites allowed for the determination of the essential genome of *S. mutans* UA159. Genes required for growth in rich and defined media were discovered. Finally, we applied Tn-seq to identify genes required for persistent colonization in a mouse model for dental caries. This study provides the foundation to identify new molecular mechanisms required for the pathogenicity of *S. mutans* and for the discovery of new targets for anticaries agents.

## RESULTS AND DISCUSSION

### The essential genome of *Streptococcus mutans* UA159.

A *Mariner in vitro* transposition mutagenesis technique was used to create *S. mutans* transposon libraries (11, 20). The *Mariner* transposable element inserts randomly at TA dinucleotide sites, which makes it well suited for use in low-G+C bacteria, such as *S. mutans* (36.82% G+C). There are 147,733 TA sites (the transposon can insert in either DNA strand) randomly distributed throughout the *S. mutans* UA159 genome. Another feature of the transposable element is the location of an MmeI restriction site in the inverted repeat that helps to facilitate Tn-seq and mapping of transposons to their insertion sites. After transposon libraries are cultured, the DNA is extracted and digested with MmeI, leaving 15 to 20 bp of chromosomal DNA that can be sequenced after ligation to suitable primers. Two transposon libraries were made in *S. mutans* UA159, a Bratthall serotype *c* strain isolated in 1982 from a child with active caries. The libraries extracted after initial isolation on blood agar contained 66,728 or 144,816 transposon insertion mutants (determined after sequencing and alignment to unique TA sites), respectively. Genomic DNA was extracted from both libraries and sequenced to determine the locations of transposon insertions. After next-generation DNA sequencing, we obtained 20,137,898 and 13,186,867 reads per library. These reads were trimmed and aligned to the *S. mutans* UA159 genome to determine the number of transposon insertions per gene. There was a strong degree of similarity between the number of insertions in each gene between the two libraries (ρ = 0.88) (see Fig. S1A in the supplemental material).

10.1128/mSphere.00031-18.1 FIG S1  Reproducibility among *S. mutans* UA159 transposon libraries is high. The total number of reads per gene (reads normalized to 1 million) per library is plotted for starter libraries selected on blood agar (A), rich media (B), and defined media (C). The number of reads per gene was highly correlated across the two transposon libraries. CPM, counts per million. Download FIG S1, PDF file, 0.3 MB. Copyright © 2018 Shields et al. 2018 Shields et al. This content is distributed under the terms of the Creative Commons Attribution 4.0 International license.

**FIG 1:**
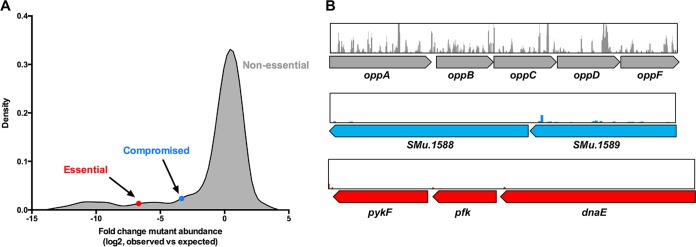
The *S. mutans* UA159 essential genome. (A) Density plot of the fold change in mutant abundance (observed versus expected reads). The blue circle indicates the point at which mutants are considered to display compromised fitness on blood agar, and the red circle indicates the point at which mutants are essential on blood agar. The majority of mutants are nonessential. (B) Example of the transposon insertion reads within genes that are considered nonessential (*oppA* to *oppF*), compromised (SMu.1588-SMu.89), and essential (*pykF*, *pfk*, and *dnaE*). Each peak represents a unique transposon insertion site, and the height of the peak relates to the number of sequencing reads for that site.

Multiple methods exist for determining essential genes from Tn-seq experiments, including annotation-dependent and -independent methods (21). Fundamentally, essential genes are defined as those that lack transposon insertions in a well-saturated transposon library that has been sequenced to saturation (each insertion has ≥50 reads on average) (21). It is known that certain genes are conditionally essential; that is, they may be required for growth in one environment, but not in others. For the purposes of this study, genes were designated essential under the conditions tested if they completely lacked transposon insertions or if the gene contained fewer than 1% of the insertions expected (based on gene size and TA site abundance; see Materials and Methods), similar to criteria applied in other Tn-seq studies (22–24). A total of 24 protein-coding genes (most often transposon related), 9 rRNA genes, and 8 tRNA genes were removed from the analysis because of multiple copies in the *S. mutans* UA159 genome (Table S1). Sixty-nine small genes (less than 100 bp) were also not included in the analysis (Table S1) on the basis that they have fewer than 10 TA sites, and therefore, transposon insertions could be underrepresented due to chance. Using these criteria, 203 genes were found to be essential for growth on blood agar, 53 of which contained no insertions and 150 had 100-fold or fewer insertions than expected (Fig. 1A and Table S2). Essential genes constituted 11% of the total annotated open reading frames (ORFs) in the *S. mutans* UA159 genome, within the range (5.3 to 22%) observed in other bacterial species (10, 17, 18, 25–28). A total of 101 genes contained 1 to 10% of the expected number of insertions. Insertions within these genes may compromise the fitness of transposon strains, but they are not essential for growth on blood agar. Examples of nonessential, compromised, and essential genes with mapped transposon insertions are shown in Fig. 1B (essential genes have very few or no transposon insertions present). Using the *S. mutans* core genome identified by Cornejo et al. (29), 177 of the 203 essential genes (87%) were part of the core genome, and the 26 remaining essential genes (13%) were in the accessory genome. Essential genes are expected to reside within the core genome, given that they encode proteins involved in fundamental biological functions and are therefore more likely to be conserved between strains (23, 30). Conversely, 100 to 300 noncore genes were estimated previously to differ between any two strains of *S. mutans* (29). When comparing the essential genomes of two strains of the same species, differences in essential gene content and metabolic pathways have been reported (26, 27). Clearly, accessory gene content is important and could alter gene-gene networks in such a way that genes that are essential in one strain become functionally redundant in another strain. Other essential functions of noncore genes may be related to coping with unique environmental conditions, associated with media and culture conditions, or from endogenously generated metabolic end products.

10.1128/mSphere.00031-18.3 TABLE S1  Genes removed prior to Tn-seq data analysis. Download TABLE S1, PDF file, 0.04 MB. Copyright © 2018 Shields et al. 2018 Shields et al. This content is distributed under the terms of the Creative Commons Attribution 4.0 International license.

10.1128/mSphere.00031-18.4 TABLE S2  Observed versus expected transposon insertion reads. Download TABLE S2, PDF file, 0.9 MB. Copyright © 2018 Shields et al. 2018 Shields et al. This content is distributed under the terms of the Creative Commons Attribution 4.0 International license.

**FIG 2:**
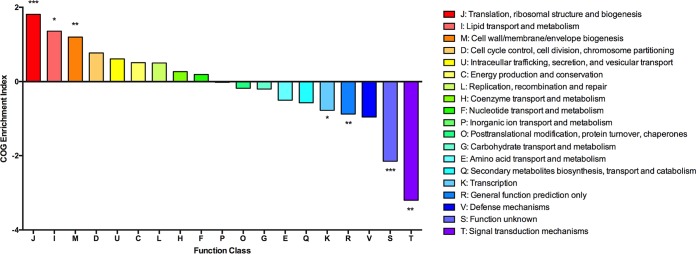
Essential genes by Clusters of Orthologous Groups (COG) classification. Essential and compromised genes that are annotated with known functions are plotted according to the COG enrichment index. This index is calculated as the percentage of the essential genome made up of a COG divided by the percentage of the whole genome made up by the same COG. The log_2_ fold enrichment is displayed, and significant differences were calculated using the two-tailed Fisher’s exact test. If the COG enrichment index for a given COG is >0, it implies that a greater proportion of genes within that COG are essential than would be expected and vice versa for a COG enrichment index of <0. Log_2_ COG enrichment index values that are significantly different from expected are indicated by asterisks as follows: *, *P* < 0.05; **, *P* < 0.01;***, *P* < 0.001.

**FIG 3:**
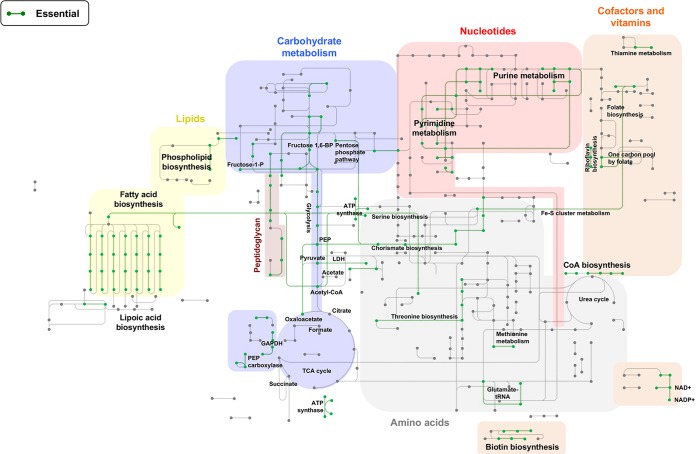
The essential metabolic pathways of *S. mutans* UA159 for the initial library recovered on blood agar. Essential metabolic pathways are indicated by green lines. Shaded areas represent major metabolic processes (e.g., carbohydrate metabolism). We have also highlighted metabolic pathways that are essential (e.g., threonine biosynthesis). Abbreviations: TCA, tricarboxylic acid; CoA, coenzyme A; GAPDH, glyceraldehyde-3-phosphate dehydrogenase; LDH, lactate dehydrogenase; PEP, phosphoenolpyruvate; P, phosphate; BP, bisphosphate.

As an additional component of our analysis, essential genes were grouped according to Clusters of Orthologous Groups (COG) functional categories (31) to yield further insights into the functions of their gene products. For this analysis, we included genes that showed evidence of compromised fitness on blood agar (0.01- to 0.1-fold expected reads). Genes encoding products related to translation, lipid metabolism, and cell wall biogenesis were enriched among essential genes (COGs J, I, and M) (Fig. 2), consistent with essential genomes identified in other bacterial species (23, 27). Genes associated with signal transduction, transcription, general functions, or of unknown function were depleted (COGs K, R, S, and T), suggesting that these functions are less important for growth on blood agar. We also visualized metabolic pathways essential for *S. mutans* using the Kyoto Encyclopedia of Genes and Genomes (KEGG) mapper (32). A total of 194 of the 203 essential genes had KEGG numbers and were included in the analysis. As shown in Fig. 3, key cellular processes associated with the functions of genes included aminoacyl-tRNA biosynthesis, glycolysis, purine and pyrimidine metabolism, fatty acid metabolism, peptidoglycan biosynthesis, ribosome biogenesis, DNA replication and repair, and pathways involved in amino acid biosynthesis. These functions broadly fit into three major biological pathways: processing of genetic information, energy production, and maintenance of the cell envelope. Eleven of the essential genes are described as hypothetical proteins without a putative function, which is not surprising considering that even in *Escherichia coli* (strain K-12 W3110) more than 40% of the protein-coding genes have not been assigned a function (33). However, system-level approaches, such as Tn-seq, should lead to better annotation of microbial genomes by facilitating the association of phenotypes with uncharacterized genes. Finally, computational analyses of Tn-seq data sets have identified drug targets in other microbial pathogens (34). Thus, identification of essential genes in *S. mutans* and their metabolic processes may delineate putative therapeutic targets against caries.

### Conservation of essential genes among streptococci.

Essential genes in *S. mutans* that are also essential in other streptococci may be effectively targeted by antimicrobial agents to control a variety of streptococcal infections, while genes that are essential only in *S. mutans* may be targeted for control of dental caries without perturbation of resident beneficial streptococci. Using the Database of Essential Genes (DEG) (http://www.essentialgene.org/) (35), we examined whether orthologs of essential genes of *S. mutans* UA159 were conserved in *Streptococcus agalactiae*, *Streptococcus pneumoniae*, *Streptococcus pyogenes*, and *Streptococcus sanguinis*. The conservation of essential genes between individual *Streptococcus* spp. and *S. mutans* ranged from 43% for *S. pneumoniae* (combination of strains R6 and Rx1) to 77% for *S. agalactiae* A909 (Table S3). More broadly, there were only 19 *S. mutans* essential genes without orthologs in any of the four streptococcal species. There was strong conservation across all four species for genes associated with fundamental cellular processes, including DNA replication, peptidoglycan synthesis, transcription and translation, lipid synthesis, and acetyl coenzyme A biochemical pathways. We also identified biological functions essential in *S. mutans*, but not in the other four streptococci. All genes involved in the shikimate pathway (*aroABCDEGHK*) were essential in *S. mutans* UA159, but not the other streptococci examined. The shikimate pathway has been identified as an attractive target for drug design against *Mycobacterium tuberculosis*, where it is also essential (36, 37). Genes for an arginine repressor (SMu.2093, *argR*), the l-lactate dehydrogenase enzyme (LDH) (SMu.1115, *ldh*), and the superoxide dismutase enzyme (SMu.629, *sod*) were also not essential in the other four streptococcal species. Hillman et al. (38) reported that LDH is essential for *S. mutans* (strain JH1000). Also, deletion of *ldh* in *S. pneumoniae* D39 led to distinct fitness defects and overaccumulation of pyruvate (39). However, the *S. pneumoniae ldh* mutant can maintain sufficient redox balance via alcohol dehydrogenase, thereby allowing *ldh* mutants to remain viable. Evidently, *S. mutans* is unable to achieve redox balance or survive toxic levels of pyruvate without LDH. The environmental niches of *S. pneumoniae* and *S. mutans* are distinct, and it is not surprising that the central metabolic processes are regulated in divergent ways. Notably, many commensal and potentially beneficial oral streptococci are members of the mitis group of streptococci, as is the pneumococcus. The connection between central carbon metabolism and *S. mutans* virulence is an important area on which to focus future Tn-seq studies.

10.1128/mSphere.00031-18.5 TABLE S3  Conservation of essential genes. Download TABLE S3, PDF file, 0.2 MB. Copyright © 2018 Shields et al. 2018 Shields et al. This content is distributed under the terms of the Creative Commons Attribution 4.0 International license.

### Metabolic requirements for survival in rich versus defined medium.

The initial selection of our transposon libraries on blood agar was designed to ensure that the maximum number of mutants was recovered. That is, blood agar is relatively nutritious, so the impact of gene deletions on auxotrophy should be reduced and growth for 48 h of well-diluted samples would increase the chances that even slow-growing colonies could be isolated. To begin to probe more deeply those genes needed for sustained growth in the laboratory, we cultured the mutant libraries in a rich liquid medium (brain heart infusion [BHI] broth) and a chemically defined medium (FMC [40]). Both media are used extensively to study *S. mutans*. We inoculated each medium with 10^7^ CFU of the mutant library, passaged the cells for approximately 30 generations, and then isolated DNA from the populations for Tn-seq. Read counts were strongly correlated between the two libraries for both conditions (rich media, ρ = 0.89; defined media, ρ = 0.90) (Fig. S1). Analysis of transposon insertion reads, using the criteria that genes yielding fewer than 1% of the expected transposon reads were defined as essential, revealed that 295 genes were essential in rich medium and 319 were required in defined medium (Table S2). Compared to essential genes on blood agar, 96 and 123 genes were differentially represented when passaged in rich or defined medium, respectively. Of these genes, 76 genes were shared across the two conditions, but 20 were unique to rich medium and 47 were unique to defined medium. Of the 101 genes that displayed compromised fitness on blood agar, i.e., yielded 1 to 10% of the expected transposon insertions compared to the library prepassage, 50 and 53 genes were essential for growth in rich and defined medium, respectively. Implicit in the interpretation of these data is the assumption that all of the genes that are essential for growth on blood agar are also essential for growth in BHI broth or FMC.

**FIG 4:**
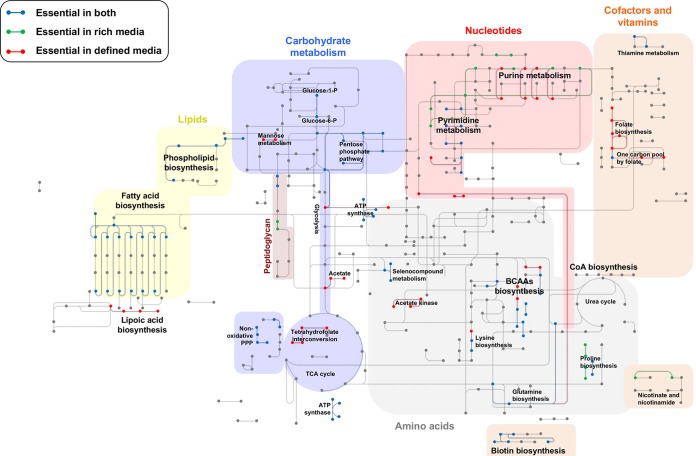
Essential metabolic pathways of *S. mutans* in rich and defined media. Metabolic pathways essential in both rich and defined media (blue lines), essential only in rich medium (green lines), and essential only in defined medium (red lines) are shown. Shaded areas represent major metabolic processes (e.g., carbohydrate metabolism). We have also highlighted metabolic pathways that become essential in these environments (e.g., acetate metabolism). BCAAs, branched-chain amino acids; TCA, tricarboxylic acid; CoA, coenzyme A; P, phosphate; PPP, pentose phosphate pathway.

Using KEGG pathway analysis, we visualized the metabolic pathways that are essential in rich medium, defined medium, or both (Fig. 4). Metabolic pathways essential in both environments include processes involved in fatty acid biosynthesis, the pentose phosphate pathway, and biotin biosynthesis. Biological processes essential differentially in defined medium included lipoteichoic acid biosynthesis (*dltABCD*), folate biosynthesis (*folADKP*), and pathways involved in pyrimidine biosynthesis (*pyrA* and *pyrAB*). In rich medium, purine (*purB* and *guaA*) and proline (*proC*) metabolism are important. Differences in requirements for gene products appear to be consistent with differences in medium composition. For instance, we would predict that BHI broth is richer in folic acid than FMC, leading to an increased demand for tetrahydrofolate production by *folADKP*. Loss of mutants with insertions in the sucrose-6-phosphate hydrolase gene *scrB* (41) in rich medium may be due to the fact that although glucose is the dominant source of carbohydrate in BHI, this medium is contaminated with small amounts of sucrose and probably other carbohydrates (42); glucose is the sole carbohydrate in the FMC medium used here. In support of this concept, insertions within the glucose/mannose-specific phosphoenolpyruvate:sugar phosphotransferase system (*manLMN*) (43) were lost from the mutant pool during passage in defined medium compared to the initial library on blood agar and the library on rich medium.

Although the clear majority (99.5%) of genes were either essential, compromised, or nonessential, a small subset of genes had increased transposon sequencing reads after passage in defined or rich media, and therefore, the mutations were interpreted to have enhanced the fitness of the strains. Alternatively, mutations within these genes may be overrepresented because they result in a phenotype with increased yields of DNA during DNA isolation versus other insertion mutants. Insertions in genes that encode proteins determining cell shape were notably overrepresented. In rich medium, overrepresented genes included *mreC* (SMu.20) and *mreD* (SMu.21), and in defined medium, *mreC*, *mreD*, *pbp2b* (SMu.597), and *rodA* (SMu.1279c) were overrepresented. Although not studied in *S. mutans*, the products of these genes contribute to peripheral peptidoglycan synthesis in *S. pneumoniae*, forming an “elongasome” consisting of MreC, MreD, RodA, RodZ, PBP2b, PBP1a, and GpsB (44). In this study, *gpsB* (SMu.471) was essential (on blood agar), and insertions within *pbp1a* (SMu.467) led to compromised fitness (in rich and defined media). The essentiality of genes that constitute the elongasome appears to be dependent on the species and strain. Indeed, in the coccus-shaped bacterium *Staphylococcus aureus* strain COL, *mreC* and *mreD* are dispensable and have no role in cell shape (45). In *S. pneumoniae*, *mreC* and *mreD* are dispensable in the unencapsulated strain R6 but required in the encapsulated strain D39 and the unencapsulated D39 Δ*cps* mutant (46). Further evaluation of these cell-shape-determining genes in *S. mutans* is warranted because it appears that the regulation of this process might differ from regulation in previously studied bacteria.

### Validation of the *in vitro* Tn-seq screen.

**FIG 5:**
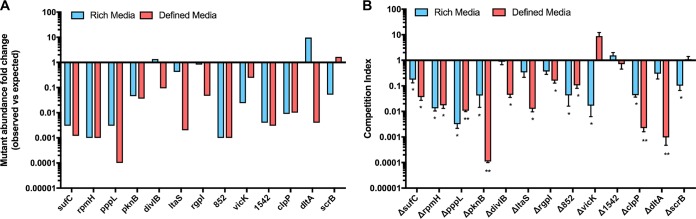
Validation of Tn-seq fitness defects in rich and defined media. (A) Tn-seq results (rich and defined media) for 13 genes (observed versus expected Tn insertion reads) displayed as a bar graph. Values less than 1 are predictive of a gene that is required for optimal fitness in that environment. (B) Bar graph depicting the fitness of mutants compared to the fitness of the wild type using a 1 × 1 competition method. A competition index of >1 indicates that the mutant grew better than the wild type did. A competition index of <1 indicates that the mutant displayed reduced fitness compared to the wild-type strain. The genes and proteins they encode follow: *sufC*, ABC transporter involved in Fe-S cluster assembly; *rpmH*, 50S ribosomal protein L34; *pppL*, phosphoprotein phosphatase; *pknB*, serine/threonine phosphatase; *divIB*, cell division protein; *ltaS*, phosphoglycerol transferase; *rgpI*, glycosyltransferase 2-like protein; SMu.852, transcriptional regulator; *vicK*, histidine kinase; SMu.1542, diacylglycerol kinase; *clpP*, ATP-dependent Clp protease; *dltA*, d-alanine-d-alanyl carrier protein ligase; *scrB*, sucrose-6-phosphate hydrolase. *, *P* < 0.05; **, *P* < 0.01.

We selected a set of genes in which insertions caused fitness reductions (essential or compromised) in rich medium, defined medium, or both to validate the data set described above. A total of 13 genes were replaced with a nonpolar kanamycin cassette and competition assays against wild-type *S. mutans* UA159 were performed (see Materials and Methods). Briefly, a 1:1 ratio of exponentially growing cultures of the wild type and each of the mutant strains were harvested at the same optical density and inoculated together to approximately 1 × 10^6^ CFU/ml in either rich or defined medium. After 20 h, equivalent to about 10 generations, mutant and wild-type *S. mutans* were serially diluted and plated. Of the strains with mutations in genes that were essential or mutant strains that showed compromised growth in Tn-seq screens in rich medium, 8 of 9 also displayed growth defects in the competition assays (Fig. 5). In defined medium, 9 out of 11 mutants were unable to compete against *S. mutans* UA159 (Fig. 5). Across both assays, 85% of the genes identified by Tn-seq screens to have reduced fitness also displayed reduced fitness during competition assays, validating our Tn-seq methodology. Other studies also reported similar levels of concurrence between fitness screens and subsequent validation (11, 47).

### Fitness determinants in a mouse model of dental caries.

**FIG 6:**
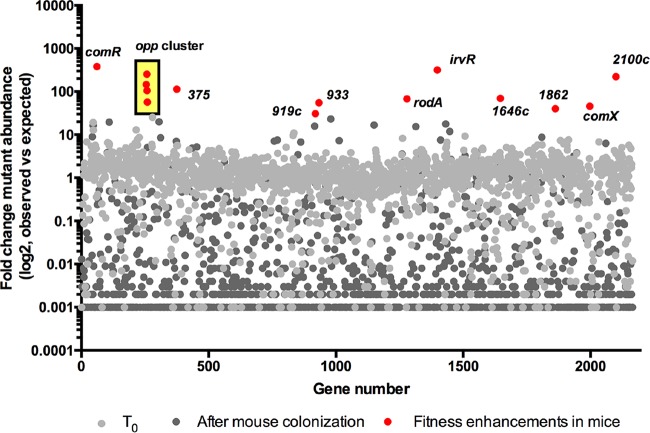
*Streptococcus mutans* UA159 fitness determinants in a rodent caries model. Tn-seq results summarized using a scatterplot that displays the fold change in mutant abundance (observed versus expected reads). Genes containing transposon insertions that lead to fitness enhancements are highlighted in red. The complete list of insertions can be found in Table S2 in the supplemental material. SMu locus tags are shown without the SMu prefix in the figure (e.g., SMu.919c is shown as *919c*).

One powerful application of high-throughput transposon screens has been to identify genes that are important during infection. We used an established rodent dental caries model (48) to interrogate our *S. mutans* transposon libraries when challenged with colonization and survival in the oral cavity. Briefly, after antibiotic suppression of oral microbiota, 7-week-old BALB/cJ mice were placed on a carbohydrate-rich diet with sweetened drinking water (2% sucrose) and orally inoculated daily for five successive days with approximately 1 × 10^8^ cells from either the library or with wild-type *S. mutans* UA159. The experiment was terminated after 3 weeks. Bacteria were collected from the mandibular molars, and DNA was isolated as outlined in Materials and Methods. Colonization of molars by *S. mutans* as a percentage of the total recovered bacterial population was similar for the transposon library and for wild-type *S. mutans*, as measured by quantitative PCR (qPCR) (24.4% ± 12.2% and 27.1% ± 12.8%, respectively). As a control, *S. mutans* was not detected from the molars of a third group consisting of uninoculated mice, even though total recovered bacteria from this group were equivalent to those from the other two groups (7.8 × 10^6^ ± 2.7 × 10^6^ from the uninoculated group, 6.5 × 10^6^ ± 5.7 × 10^6^ from the group inoculated with wild-type *S. mutans*, and 8.1 × 10^6^ ± 6.3 × 10^6^ from the group inoculated with streptococci from the transposon library). These results indicate our protocols were effective in preventing cross-contamination between animal groups and that total molar colonization by the transposon library and wild-type *S. mutans* were similar, averaging approximately 2.5 × 10^6^ *S. mutans* per mouse. From the 16 mice inoculated with the transposon library, we obtained 3.6 μg of bacterial DNA from the mandibular molars, and this DNA was used to generate Tn-seq libraries. After next-generation DNA sequencing, we obtained a total of 9.28 × 10^7^ sequenced reads, of which 8.98 × 10^7^ remained after quality control and trimming of adapter and barcode sequences. Transposon insertion mutations were recovered from 1,725 of the 1,940 genes within the *S. mutans* UA159 genome (small and duplicate genes were removed from the analysis). After read analysis, negative selection (less than 1% of expected transposon reads) was observed for 79% of the *S. mutans* UA159 genome (Fig. 6 and Table S2) comparing the input library to the output library. In other Tn-seq studies, negative selection was also the dominant selective process in *in vivo* experiments (49, 50). Although it is surprising that >75% of the genes in *S. mutans* UA159 are required for colonization of the mouse oral cavity, there is obviously considerable selective pressure during the initial colonization of and then survival during competition with resurgent commensal bacteria for 3 weeks in the mouse oral cavity. Notably, mutant strains carrying transposon insertions in genes involved in genetic competence (i.e., *comR*, *oppABCDF*, *irvR*, and *comX*) showed an enhancement in recovery from the mouse oral cavity. Insertions within these genes were 50-fold (*comX*) to 400-fold (*comR*) higher than would be expected if Tn insertions within these genes had no effect on fitness (Fig. 5B). The “blooming” of these Tn insertions likely partly accounts for the low number of Tn insertions recovered from most other genes. Genetic competence is required for the internalization of extracellular DNA and has been linked to other behaviors, including stress tolerance and biofilm formation (51, 52). Both ComR and the Opp oligopeptide permease transporter are required for the activation of transcription of *comX* (often called *sigX*), encoding the alternative sigma factor that controls late competence gene activation (53, 54). IrvR is a regulator important for the development of genetic competence in *S. mutans* (55). In general, the fitness of strains carrying mutations in genes required for genetic competence has rarely been assessed in *in vivo* models. In one study, *S. pneumoniae* virulence was attenuated in a Δ*comX1* Δ*comX2* mutant, apparently due to loss of induction of the allolytic genes *cbpD* and *cibAB* (56). Orthologs of *cbpD* and *cibAB* are not present in the *S. mutans* UA159 genome. Notably, production of ComX was shown to lead to growth arrest and cell lysis in *S. mutans* (57), so fitness enhancements in competence-related mutants may account for the increases in recovery of these mutants. Alternatively, *comRS* and many of the competence genes are highly conserved across human isolates of *S. mutans* (58), so the possibility that the mouse caries model may not fully recapitulate the selective pressures found in a human host must be considered. One notable difference in our mouse model from the natural environment in humans is that we suppressed the endogenous flora with antibiotics prior to infecting with *S. mutans*. Thus, the competence genes may play important roles in competition with commensal bacteria.

Rodent models, more commonly involving rats, have been used to study dental caries for decades, and have made important contributions to the understanding of molecular components of the virulence of *S. mutans* (59–61). Potential limitations of *in vivo* Tn-seq experiments include infection bottlenecks (whereby most transposon mutants are lost during inoculation), complementation by other mutants (e.g., secreted products), the time point chosen for infection or collection of organisms, and differences in the microbiome of animals versus the human oral cavity. Bottlenecks are a major technical challenge for *in vivo* Tn-seq experiments (62). There was a significant bottleneck associated with this study (0.5% of the original inoculum was recovered), but mutants were recovered from 90% of the *S. mutans* UA159 genome in the 16 animals. Now that a protocol that allows for *in vivo* screening of libraries in the oral cavity has been established, it should be possible to further explore these *in vivo* phenotypes under different experimental conditions to ascertain how diet and other factors could influence the colonization, persistence, and virulence of *S. mutans*.

### Limitations of Tn-seq analysis.

Here, we provide the first comprehensive overview of the essential genome of *S. mutans* UA159, but at the same time, we acknowledge the limitations of this technique. One, all transposons (Tn*5*, Tn*7*, Tn*10*, or *Mariner*) have insertion biases whereby the sequence of a region of the genome could lead to “cold spots.” Two, small genes can be misidentified as essential because of a lack of transposon insertions due to either too few insertion sites (TA dinucleotide sites in the case of *Mariner*) or a lack of transposon insertion density. We employed stringent criteria to disregard small genes. However, included within our analysis are genes that are classified as essential but that may be sufficiently small to be nonessential. A third issue relates to the initial selection of the library, whereby a limited number of genes are classified as essential but they are truly conditionally essential. For example, *sod* (SMu.629), encoding superoxide dismutase (SOD), was determined to be essential by our methodology but was previously shown to be nonessential in *S. mutans* under both aerobic and anaerobic environments (63). The absence of a growth defect for *sod* mutants in aerobic environments is somewhat surprising. SOD is employed by nearly all living organisms as an antioxidant defense and might be assumed to have increased prominence in facultative anaerobes, such as *S. mutans*. Our efforts to delete *sod* by PCR ligation mutagenesis in aerobic environments failed but were successful if the transformation was kept strictly anaerobic (Fig. S2A). The *sod* mutants had 1,000-fold-diminished competitiveness versus the wild type when grown in BHI in an aerobic environment (Fig. S2B). Therefore, *sod* essentiality is related to the conditions of initial selection, and this gene is not essential in all environments. As an aside, we observed evidence that extragenic suppression of the *sod* deletion may occur, which may further explain the discrepancies between our results and the previous report (63). Genes may also be classified as essential because strains lacking these genes display such a severe growth defect that they never replicate enough to be identified during library preparation and sequencing. Last, the polarity of the transposon insertion may also lead to incorrect recording of an essential gene, although this is thought to be a minor issue with the *magellan6* transposon (11).

10.1128/mSphere.00031-18.2 FIG S2  Validation of *sodA* as a conditionally essential gene. (A) PCR ligation mutagenesis (see Materials and Methods) was employed to replace the *sodA* gene with a nonpolar kanamycin cassette on both BHI (brain heart infusion) and blood agar, with incubation at 37°C in anaerobic, aerobic, and microaerophilic environments. Kanamycin-resistant colonies were obtained only if transformations were kept strictly anaerobic, except for one colony isolated in the microaerophilic conditions (white arrow). (B) Isolated *sodA* mutants (Km^r^), kept strictly anaerobic during routine culture, were competed against wild-type *S. mutans* in both anaerobic (BHI) and aerobic (BHI, with shaking at 150 rpm) environments. A competition index of >1 indicates that the mutant grew better than the wild type did. A competition index of <1 indicates that the mutant displayed reduced fitness compared to the wild-type strain. Download FIG S2, PDF file, 2.2 MB. Copyright © 2018 Shields et al. 2018 Shields et al. This content is distributed under the terms of the Creative Commons Attribution 4.0 International license.

Limitations exist for other methods, including gold standard gene deletion libraries such as the Keio collection in *Escherichia coli* (9). In this comprehensive library, genes were denoted as nonessential but have since been shown to be essential. An example is *holD*, a gene denoted as nonessential in the Keio collection. However, it has been shown that Δ*holD* mutants accumulate suppressor mutations that override the essentiality of the gene (64). Therefore, a complete understanding of essential genes will likely require using multiple approaches, combining gene deletion libraries with modern technologies, such as Tn-seq. Furthermore, complete validation via essential gene knockdowns employing antisense RNA or clustered regularly interspaced short palindromic repeat (CRISPR) interference assays can also provide further confirmation of essential genes.

### Conclusions.

We developed and validated Tn-seq technology for use in *S. mutans* using a *Mariner* minitransposon and next-generation DNA sequencing. We determined the essential genome for particular *in vitro* conditions and identified fitness determinants required for establishment and/or persistence in an animal model. Going forward, Tn-seq methods are sufficiently flexible for use under conditions to model processes important for the colonization, growth, stress tolerance, and persistence of genetically competent strains of *S. mutans* as well as related streptococci. In addition, Tn-seq may highlight metabolic pathways that are interesting candidates to disrupt oral colonization by *S. mutans*.

## MATERIALS AND METHODS

### Strains and growth conditions.

*S. mutans* UA159 and mutant strains are listed in Table S4 in the supplemental material. *S. mutans* strains were routinely cultured in brain heart infusion (BHI) broth at 37°C in a 5% CO_2_ microaerophilic environment. The chemically defined medium FMC (40) was used during Tn-seq and competition assay studies. Antibiotics were used at the following concentrations: kanamycin (1.0 mg/ml for *S. mutans*; 50 µg/ml for *E. coli*), spectinomycin (1.0 mg/ml for *S. mutans*; 50 µg/ml for *E. coli*) and ampicillin (100 µg/ml for *E. coli*). A list of strains and plasmids (Table S4) and oligonucleotide primers (Table S5) can be found in the supplemental material.

10.1128/mSphere.00031-18.6 TABLE S4  List of strains and plasmids used in this study. Download TABLE S4, PDF file, 0.05 MB. Copyright © 2018 Shields et al. 2018 Shields et al. This content is distributed under the terms of the Creative Commons Attribution 4.0 International license.

10.1128/mSphere.00031-18.7 TABLE S5  Oligonucleotides used in this study. Download TABLE S5, PDF file, 0.1 MB. Copyright © 2018 Shields et al. 2018 Shields et al. This content is distributed under the terms of the Creative Commons Attribution 4.0 International license.

### Ethics statement.

The University of Florida IACUC committee approved all animal procedures (IACUC study 201509214).

### Tn-seq experiments.

Transposon libraries were created following a previously published *in vitro* transposition technique with minor modifications (20). Genomic DNA from *S. mutans* UA159 was combined with the transposon Magellan6 and the transposase MarC9, and the resulting transposon DNA was repaired. This DNA was transformed into *S. mutans*, and transposon mutants were selected on blood agar containing spectinomycin with incubation for 2 days at 37°C in a 5% CO_2_ microaerophilic environment. Transposon mutants were collected and stored at −80°C. Two libraries were created in *S. mutans* UA159, and selection was carried out in rich medium, defined medium, and an *in vivo* mouse model. After selection, genomic DNA was obtained from each sample and digested with MmeI, prior to adapter ligation (see Table S5 for adapter sequences), as previously described (20). After Illumina sequencing, FASTQ sequencing files were processed on the University of Florida Research Computing Galaxy Instance. Files were split according to the sample barcode (Barcode Splitter version 1.0.0) and trimmed of the 8-nucleotide barcode (Trimmomatic version 0.32.2). The transposon sequence was removed (ACAGGTTGGATGATAA; Clip version 1.0.1), and the resulting 14- to 16-bp genomic DNA sequence was aligned to the *S. mutans* UA159 genome using Bowtie 2 (65). Importantly, 15% of the 3′ end of genes was removed from analysis, as insertions in this region may not interrupt gene function. Transposon insertion counts per gene were calculated using HTSeq-count (66). To determine gene essentiality, an annotation-dependent method was used (22). In short, the expected number of reads per gene was calculated based on gene size and transposon library size, assuming total randomness of Tn insertion (normalized to 1 million reads). The observed reads per gene (normalized to 1 million reads) was divided by the expected reads obtaining a ratio. For a gene, a ratio of 0.01 to 0.1 indicates compromised fitness, with a ratio less than 0.01 indicating essentiality of that gene.

### Essential gene function and metabolic network analysis.

For the *S. mutans* UA159 genome, genes were assigned functions based on COG and KEGG annotations. Approximately 60% of *S. mutans* genes have been assigned a KEGG number. Five hundred eighty-nine *S. mutans* genes have not been assigned to a COG category. For each COG, the total number of genes related to that function (across the entire genome) and the total number of essential genes related to that function were counted in Excel. COG enrichment was determined using a previously described method (27). Metabolic networks were generated using KEGG Mapper (http://www.genome.jp/kegg/mapper.html) (32) and a previously described protocol (27).

### Essential gene conservation.

We used the Database of Essential Genes (http://www.essentialgene.org) (35) to compare the essential genes of *S. mutans* UA159 to those of *Streptococcus agalactiae*, *Streptococcus pneumoniae*, *Streptococcus pyogenes*, and *Streptococcus sanguinis*. If a gene was not essential in these four species, then a comparison was made against all known essential genes (from the essential genomes of 46 bacterial species). For each essential gene, the protein sequence was used with default BLASTP parameters.

### Gene mutagenesis.

Standard DNA manipulation techniques were used to engineer deletion strains (67). A PCR ligation mutagenesis method was used to replace genes with nonpolar kanamycin markers (68). For each gene deletion, primers A and B were designed to amplify 500 to 600 bp upstream of the coding sequence (with ca. 50 bp overlapping the coding sequence of the gene). Primers C and D were designed to amplify 500 to 600 bp downstream of the coding sequence (with ca. 50 bp overlapping the coding sequence of the gene). Primers B and C contain BamHI restriction enzyme sites for ligation of the AB and CD fragments to a nonpolar kanamycin cassette (lacking both a promoter and terminator) digested from plasmid pALH124 (69). The nonpolar kanamycin cassette was always inserted in the same direction as the open reading frame it was replacing. Transformants were selected on BHI agar containing kanamycin. Double-crossover recombination, without introduction of nearby secondary mutations, was confirmed by PCR and Sanger sequencing using primers E and F, away from the site of recombination.

### Mouse model.

The mouse caries protocol was performed as previously described (48) with modifications. Inbred 5-week-old female specific-pathogen-free (SPF) BALB/cJ mice (The Jackson Laboratory) were allowed to acclimate for 2 days after shipping. Mice were then provided drinking water containing 0.8 mg/ml sulfamethoxazole and 0.16 mg/ml trimethoprim for 10 days to suppress indigenous oral bacteria. After a 3-day washout period with no antibiotics, mice were placed on a diet of irradiated powdered AIN-93G purified diet without fluoride, but with increased vitamins to compensate for irradiation (Envigo, Madison, WI). This diet contains 37.5% total sucrose, 24% corn starch, 17.7% protein as casein, and 4.45% maltodextrin. Mice were also given sterile water containing 2% sucrose. A total of 16 mice were inoculated with one of the transposon mutant libraries. Mice were inoculated each day for 5 days with ~1 × 10^8^ cells in 100 µl of 1.5% carboxymethyl cellulose in sterile distilled water (dH_2_O). As additional controls, 16 mice were used as uninoculated controls, and a group of 8 mice were inoculated at the same time with wild-type *S. mutans* UA159. After 21 days, mice were euthanized by CO_2_ asphyxiation followed by cervical dislocation. Mandibles were aseptically extracted, dissected to isolate molar teeth, and then sonicated on ice in 1 ml sterile phosphate-buffered saline (PBS) using a Fisher F60 sonic dismembrator (100 W and 22.5 kHz) with six pulses (each pulse 10 s long) at 90-s intervals. To obtain linear standard curves, cells in quantitative PCR (qPCR) assays (see below), we added approximately 5 × 10^8^ cells of *S. mitis* laboratory strain UF2 to each sonicate. *S. mitis* cells first underwent depurination (two 1-h incubations in 0.2 N HCl at 70°C) with extensive neutralization and washing in PBS. DNA was recovered from cell pellets obtained by centrifugation at 10,000 × *g* for 10 min at 4°C using the UltraClean microbial DNA isolation kit (Mo Bio Labs, Inc., Carlsbad, CA). Quantitative PCR was used to estimate total recovered bacteria and *S. mutans*. Primers (0.5 µM) specific for *S. mutans* were directed against the SMu.292 gene, a putative transcriptional regulator (forward primer, 5′-TGGCAAGTCCTGATGGTTTGAC-3′; reverse primer, 5′-GGAAGCGGAAGCTGTGATGAAC-3′). PCR mixtures (20 µl) were run on a Bio-Rad CFX96 thermocycler using SsoAdvanced Universal SYBR green supermix (Bio-Rad) as follows: (i) 3 min at 98°C; (ii) 40 cycles, with 1 cycle consisting of 15 s at 98°C and 45 s at 68°C. To estimate the total number of bacteria, we developed degenerate primers to conserved regions of the ubiquitous single-copy gene *rpsL* (30S ribosomal protein S12) (70) after ClustalW alignment (MacVector v.12) of sequences from 22 different species of oral and nonoral streptococci, two strains of *Lactobacillus murinus* (ASF361 and DSM20452), two species of *Staphylococcus* (*Staphylococcus saprophyticus* 772 and *Staphylococcus xylosus* HKUOPL8), three species of *Corynebacterium* (*Corynebacterium pilosum* CIP103422, *Corynebacterium renale* CIP52.96, and *Corynebacterium mastitidis* DSM44356), and *Muribacter* *muris* Ackerman 80-443-D. These organisms represent taxa, and include species, identified previously in SPF mice under biosafety level 2 (BSL2) conditions (48). Reaction conditions were similar to those for SMu.292 except the annealing temperature was 55°C for 30 cycles and with 2.5 µM primers (forward primer, 5′-CCKAAYTCNGCNYTNCGTAAR-3′; reverse primer, 5′-CGHACMCCHGGDARGTCYTT-3′). Standard curves were derived from DNA isolated from 10^3^ to 10^8^ *S. mutans* grown to mid-exponential phase in BHI. Cell numbers were determined from optical density at 600 nm (OD_600_) based on a growth curve of CFU versus OD_600_. Quantification cycle (*C*_*q*_) values (in triplicate) were analyzed using CFX Manager software. The efficiencies, slopes, and *r*^2^ values for standard curves ranged from 97 to 105%, −3.274 to −3.323, and 0.985 to 0.998, respectively. As controls, primers to SMu.292 failed to generate a product when tested against DNA from bacteria isolated from oral swabs of mice prior to administration of antibiotics and grown overnight in BHI plus 2% yeast extract under anaerobic conditions. Also, both primer sets failed to produce a product against 5 × 10^8^ *S. mitis* carrier cells after processing for DNA.

### Competition assays.

For competition assays, strains were cultured in either rich (BHI) or defined (FMC) medium. An inoculum of 1 × 10^6^ CFU/ml of the wild-type strain and an inoculum of each mutant strain to be tested were added to prewarmed medium and cultured for 20 h in a microaerophilic environment at 37°C. At both the start and end of the experiment, bacteria were serially diluted and plated onto BHI and BHI-kanamycin agar. Wild-type and mutant strains were enumerated (wild-type CFU were derived by subtracting mutant CFU from total CFU on BHI agar), and the competitive index was calculated using the following formula: (*t*_end_ mutant CFU/*t*_end_ wild-type CFU)/(*t*_start_ mutant CFU/*t*_start_ wild-type) where *t*_end_ mutant CFU is the number of mutant CFU at the end of the experiment and *t*_start_ mutant CFU is the number of mutant CFU at the beginning of the experiment.

## References

[1] Kassebaum NJ, Bernabé E, Dahiya M, Bhandari B, Murray CJL, Marcenes W 2015 Global burden of untreated caries: a systematic review and metaregression. J Dent Res 94:650–658. doi:10.1177/0022034515573272. 25740856

[2] Richards VP, Alvarez AJ, Luce AR, Bedenbaugh M, Mitchell ML, Burne RA, Nascimento MM 2017 Microbiomes of site-specific dental plaques from children with different caries status. Infect Immun 85:e00106-17. doi:10.1128/IAI.00106-17. 28507066 PMC5520424

[3] Gross EL, Beall CJ, Kutsch SR, Firestone ND, Leys EJ, Griffen AL 2012 Beyond *Streptococcus mutans*: dental caries onset linked to multiple species by 16S rRNA community analysis. PLoS One 7:e47722. doi:10.1371/journal.pone.0047722. 23091642 PMC3472979

[4] Johansson I, Witkowska E, Kaveh B, Lif Holgerson P, Tanner ACR 2016 The microbiome in populations with a low and high prevalence of caries. J Dent Res 95:80–86. doi:10.1177/0022034515609554. 26442950 PMC4700664

[5] Ajdić D, McShan WM, McLaughlin RE, Savić G, Chang J, Carson MB, Primeaux C, Tian R, Kenton S, Jia H, Lin S, Qian Y, Li S, Zhu H, Najar F, Lai H, White J, Roe BA, Ferretti JJ 2002 Genome sequence of *Streptococcus mutans* UA159, a cariogenic dental pathogen. Proc Natl Acad Sci U S A 99:14434–14439. doi:10.1073/pnas.172501299. 12397186 PMC137901

[6] Lemos JA, Burne RA 2008 A model of efficiency: stress tolerance by *Streptococcus mutans*. Microbiology 154:3247–3255. doi:10.1099/mic.0.2008/023770-0. 18957579 PMC2627771

[7] Nakano K, Nomura R, Ooshima T 2008 *Streptococcus mutans* and cardiovascular diseases. Jpn Dent Sci Rev 44:29–37. doi:10.1016/j.jdsr.2007.09.001.

[8] Nakano K, Hokamura K, Taniguchi N, Wada K, Kudo C, Nomura R, Kojima A, Naka S, Muranaka Y, Thura M, Nakajima A, Masuda K, Nakagawa I, Speziale P, Shimada N, Amano A, Kamisaki Y, Tanaka T, Umemura K, Ooshima T 2011 The collagen-binding protein of *Streptococcus mutans* is involved in haemorrhagic stroke. Nat Commun 2:485. doi:10.1038/ncomms1491. 21952219 PMC3220351

[9] Baba T, Ara T, Hasegawa M, Takai Y, Okumura Y, Baba M, Datsenko KA, Tomita M, Wanner BL, Mori H 2006 Construction of *Escherichia coli* K-12 in-frame, single-gene knockout mutants: the Keio collection. Mol Syst Biol 2:2006.0008. doi:10.1038/msb4100050. PMC1681482 16738554

[10] Xu P, Ge X, Chen L, Wang X, Dou Y, Xu JZ, Patel JR, Stone V, Trinh M, Evans K, Kitten T, Bonchev D, Buck GA 2011 Genome-wide essential gene identification in *Streptococcus sanguinis*. Sci Rep 1:125. doi:10.1038/srep00125. 22355642 PMC3216606

[11] van Opijnen T, Bodi KL, Camilli A 2009 Tn-seq: high-throughput parallel sequencing for fitness and genetic interaction studies in microorganisms. Nat Methods 6:767–772. doi:10.1038/nmeth.1377. 19767758 PMC2957483

[12] Forsyth RA, Haselbeck RJ, Ohlsen KL, Yamamoto RT, Xu H, Trawick JD, Wall D, Wang L, Brown-Driver V, Froelich JM, Kedar GC, King P, McCarthy M, Malone C, Misiner B, Robbins D, Tan Z, Zhu Z-Y, Carr G, Mosca DA, Zamudio C, Foulkes JG, Zyskind JW 2002 A genome-wide strategy for the identification of essential genes in *Staphylococcus aureus*. Mol Microbiol 43:1387–1400. doi:10.1046/j.1365-2958.2002.02832.x. 11952893

[13] Salama NR, Shepherd B, Falkow S 2004 Global transposon mutagenesis and essential gene analysis of *Helicobacter pylori*. J Bacteriol 186:7926–7935. doi:10.1128/JB.186.23.7926-7935.2004. 15547264 PMC529078

[14] Akerley BJ, Rubin EJ, Novick VL, Amaya K, Judson N, Mekalanos JJ 2002 A genome-scale analysis for identification of genes required for growth or survival of *Haemophilus influenzae*. Proc Natl Acad Sci U S A 99:966–971. doi:10.1073/pnas.012602299. 11805338 PMC117414

[15] Quivey RG, Grayhack EJ, Faustoferri RC, Hubbard CJ, Baldeck JD, Wolf AS, MacGilvray ME, Rosalen PL, Scott-Anne K, Santiago B, Gopal S, Payne J, Marquis RE 2015 Functional profiling in *Streptococcus mutans*: construction and examination of a genomic collection of gene deletion mutants. Mol Oral Microbiol 30:474–495. doi:10.1111/omi.12107. 25973955 PMC4636983

[16] Langridge GC, Phan MD, Turner DJ, Perkins TT, Parts L, Haase J, Charles I, Maskell DJ, Peters SE, Dougan G, Wain J, Parkhill J, Turner AK 2009 Simultaneous assay of every *Salmonella* Typhi gene using one million transposon mutants. Genome Res 19:2308–2316. doi:10.1101/gr.097097.109. 19826075 PMC2792183

[17] Le Breton Y, Belew AT, Valdes KM, Islam E, Curry P, Tettelin H, Shirtliff ME, El-Sayed NM, McIver KS 2015 Essential genes in the core genome of the human pathogen *Streptococcus pyogenes*. Sci Rep 5:9838. doi:10.1038/srep09838. 25996237 PMC4440532

[18] Hooven TA, Catomeris AJ, Akabas LH, Randis TM, Maskell DJ, Peters SE, Ott S, Santana-Cruz I, Tallon LJ, Tettelin H, Ratner AJ 2016 The essential genome of *Streptococcus agalactiae*. BMC Genomics 17:406. doi:10.1186/s12864-016-2741-z. 27229469 PMC4881062

[19] Shields RC, O’Brien G, Maricic N, Kesterson A, Grace M, Hagen SJ, Burne RA 2017 Genome-wide screens reveal new gene products that influence genetic competence in *Streptococcus mutans*. J Bacteriol 200:e00508-17. doi:10.1128/JB.00508-17. PMC5738738 29109185

[20] van Opijnen T, Camilli A 2010 Genome-wide fitness and genetic interactions determined by Tn-seq, a high-throughput massively parallel sequencing method for microorganisms. Curr Protoc Microbiol Chapter 1:Unit 1E.3. doi:10.1002/9780471729259.mc01e03s19. PMC3877651 21053251

[21] Chao MC, Abel S, Davis BM, Waldor MK 2016 The design and analysis of transposon insertion sequencing experiments. Nat Rev Microbiol 14:119–128. doi:10.1038/nrmicro.2015.7. 26775926 PMC5099075

[22] Valentino MD, Foulston L, Sadaka A, Kos VN, Villet RA, Santa Maria J, Lazinski DW, Camilli A, Walker S, Hooper DC, Gilmore MS 2014 Genes contributing to *Staphylococcus aureus* fitness in abscess- and infection-related ecologies. mBio 5:e01729-14. doi:10.1128/mBio.01729-14. 25182329 PMC4173792

[23] Klein BA, Tenorio EL, Lazinski DW, Camilli A, Duncan MJ, Hu LT 2012 Identification of essential genes of the periodontal pathogen *Porphyromonas gingivalis*. BMC Genomics 13:578. doi:10.1186/1471-2164-13-578. 23114059 PMC3547785

[24] McDonough E, Lazinski DW, Camilli A 2014 Identification of *in vivo* regulators of the *Vibrio cholerae* *xds* gene using a high-throughput genetic selection. Mol Microbiol 92:302–315. doi:10.1111/mmi.12557. 24673931 PMC4005888

[25] Lee SA, Gallagher LA, Thongdee M, Staudinger BJ, Lippman S, Singh PK, Manoil C 2015 General and condition-specific essential functions of *Pseudomonas aeruginosa*. Proc Natl Acad Sci U S A 112:5189–5194. doi:10.1073/pnas.1422186112. 25848053 PMC4413342

[26] Turner KH, Wessel AK, Palmer GC, Murray JL, Whiteley M 2015 Essential genome of *Pseudomonas aeruginosa* in cystic fibrosis sputum. Proc Natl Acad Sci U S A 112:4110–4115. doi:10.1073/pnas.1419677112. 25775563 PMC4386324

[27] Narayanan AM, Ramsey MM, Stacy A, Whiteley M 2017 Defining genetic fitness determinants and creating genomic resources for an oral pathogen. Appl Environ Microbiol 83:e00797-17. doi:10.1128/AEM.00797-17. 28476775 PMC5494627

[28] Wong Y-C, Abd El Ghany M, Naeem R, Lee K-W, Tan Y-C, Pain A, Nathan S 2016 Candidate essential genes in *Burkholderia cenocepacia* J2315 identified by genome-wide TraDIS. Front Microbiol 7:1288. doi:10.3389/fmicb.2016.01288. 27597847 PMC4993015

[29] Cornejo OE, Lefébure T, Bitar PDP, Lang P, Richards VP, Eilertson K, Do T, Beighton D, Zeng L, Ahn S-J, Burne RA, Siepel A, Bustamante CD, Stanhope MJ 2013 Evolutionary and population genomics of the cavity causing bacteria *Streptococcus mutans*. Mol Biol Evol 30:881–893. doi:10.1093/molbev/mss278. 23228887 PMC3603310

[30] Jordan IK, Rogozin IB, Wolf YI, Koonin EV 2002 Essential genes are more evolutionarily conserved than are nonessential genes in bacteria. Genome Res 12:962–968. doi:10.1101/gr.87702. 12045149 PMC1383730

[31] Tatusov RL, Galperin MY, Natale DA, Koonin EV 2000 The COG database: a tool for genome-scale analysis of protein functions and evolution. Nucleic Acids Res 28:33–36. doi:10.1093/nar/28.1.33. 10592175 PMC102395

[32] Kanehisa M, Goto S, Sato Y, Furumichi M, Tanabe M 2012 KEGG for integration and interpretation of large-scale molecular data sets. Nucleic Acids Res 40:D109–D114. doi:10.1093/nar/gkr988. 22080510 PMC3245020

[33] Vlasblom J, Zuberi K, Rodriguez H, Arnold R, Gagarinova A, Deineko V, Kumar A, Leung E, Rizzolo K, Samanfar B, Chang L, Phanse S, Golshani A, Greenblatt JF, Houry WA, Emili A, Morris Q, Bader G, Babu M 2015 Novel function discovery with GeneMANIA: a new integrated resource for gene function prediction in *Escherichia coli*. Bioinformatics 31:306–310. doi:10.1093/bioinformatics/btu671. 25316676 PMC4308668

[34] Mobegi FM, van Hijum SAFT, Burghout P, Bootsma HJ, de Vries SPW, van der Gaast-de Jongh CE, Simonetti E, Langereis JD, Hermans PWM, de Jonge MI, Zomer A 2014 From microbial gene essentiality to novel antimicrobial drug targets. BMC Genomics 15:958. doi:10.1186/1471-2164-15-958. 25373505 PMC4233050

[35] Luo H, Lin Y, Gao F, Zhang C-T, Zhang R 2014 DEG 10, an update of the database of essential genes that includes both protein-coding genes and noncoding genomic elements. Nucleic Acids Res 42:D574–D580. doi:10.1093/nar/gkt1131. 24243843 PMC3965060

[36] Parish T, Stoker NG 2002 The common aromatic amino acid biosynthesis pathway is essential in *Mycobacterium tuberculosis*. Microbiology 148:3069–3077. doi:10.1099/00221287-148-10-3069. 12368440

[37] Reichau S, Jiao W, Walker SR, Hutton RD, Baker EN, Parker EJ 2011 Potent inhibitors of a shikimate pathway enzyme from *Mycobacterium tuberculosis*: combining mechanism- and modeling-based design. J Biol Chem 286:16197–16207. doi:10.1074/jbc.M110.211649. 21454647 PMC3093739

[38] Hillman JD, Chen A, Duncan M, Lee SW 1994 Evidence that l-(+)-lactate dehydrogenase deficiency is lethal in *Streptococcus mutans*. Infect Immun 62:60–64. 8262650 10.1128/iai.62.1.60-64.1994 PMC186067

[39] Gaspar P, Al-Bayati FAY, Andrew PW, Neves AR, Yesilkaya H 2014 Lactate dehydrogenase is the key enzyme for pneumococcal pyruvate metabolism and pneumococcal survival in blood. Infect Immun 82:5099–5109. doi:10.1128/IAI.02005-14. 25245810 PMC4249287

[40] Terleckyj B, Willett NP, Shockman GD 1975 Growth of several cariogenic strains of oral streptococci in a chemically defined medium. Infect Immun 11:649–655. 1091546 10.1128/iai.11.4.649-655.1975 PMC415117

[41] Hayakawa M, Aoki H, Kuramitsu HK 1986 Isolation and characterization of the sucrose 6-phosphate hydrolase gene from *Streptococcus mutans*. Infect Immun 53:582–586. 3017864 10.1128/iai.53.3.582-586.1986 PMC260830

[42] Zeng L, Burne RA 2013 Comprehensive mutational analysis of sucrose-metabolizing pathways in *Streptococcus mutans* reveals novel roles for the sucrose phosphotransferase system permease. J Bacteriol 195:833–843. doi:10.1128/JB.02042-12. 23222725 PMC3562097

[43] Abranches J, Chen Y-YM, Burne RA 2003 Characterization of *Streptococcus mutans* strains deficient in EIIAB Man of the sugar phosphotransferase system. Appl Environ Microbiol 69:4760–4769. doi:10.1128/AEM.69.8.4760-4769.2003. 12902269 PMC169087

[44] Massidda O, Nováková L, Vollmer W 2013 From models to pathogens: how much have we learned about *Streptococcus pneumoniae* cell division? Environ Microbiol 15:3133–3157. doi:10.1111/1462-2920.12189. 23848140

[45] Tavares AC, Fernandes PB, Carballido-López R, Pinho MG 2015 MreC and MreD proteins are not required for growth of *Staphylococcus aureus*. PLoS One 10:e0140523. doi:10.1371/journal.pone.0140523. 26470021 PMC4607420

[46] Land AD, Winkler ME 2011 The requirement for pneumococcal MreC and MreD is relieved by inactivation of the gene encoding PBP1a. J Bacteriol 193:4166–4179. doi:10.1128/JB.05245-11. 21685290 PMC3147673

[47] van Opijnen T, Dedrick S, Bento J 2016 Strain dependent genetic networks for antibiotic-sensitivity in a bacterial pathogen with a large pan-genome. PLoS Pathog 12:e1005869. doi:10.1371/journal.ppat.1005869. 27607357 PMC5015961

[48] Culp DJ, Robinson B, Cash MN, Bhattacharyya I, Stewart C, Cuadra-Saenz G 2015 Salivary mucin 19 glycoproteins: innate immune functions in *Streptococcus mutans*-induced caries in mice and evidence for expression in human saliva. J Biol Chem 290:2993–3008. doi:10.1074/jbc.M114.597906. 25512380 PMC4317041

[49] Skurnik D, Roux D, Aschard H, Cattoir V, Yoder-Himes D, Lory S, Pier GB 2013 A comprehensive analysis of *in vitro* and *in vivo* genetic fitness of *Pseudomonas aeruginosa* using high-throughput sequencing of transposon libraries. PLoS Pathog 9:e1003582. doi:10.1371/journal.ppat.1003582. 24039572 PMC3764216

[50] Gao B, Vorwerk H, Huber C, Lara-Tejero M, Mohr J, Goodman AL, Eisenreich W, Galán JE, Hofreuter D 2017 Metabolic and fitness determinants for in vitro growth and intestinal colonization of the bacterial pathogen *Campylobacter jejuni*. PLoS Biol 15:e2001390. doi:10.1371/journal.pbio.2001390. 28542173 PMC5438104

[51] Seaton K, Ahn S-J, Sagstetter AM, Burne RA 2011 A transcriptional regulator and ABC transporters link stress tolerance, (p)ppGpp, and genetic competence in *Streptococcus mutans*. J Bacteriol 193:862–874. doi:10.1128/JB.01257-10. 21148727 PMC3028664

[52] Li Y-H, Tang N, Aspiras MB, Lau PCY, Lee JH, Ellen RP, Cvitkovitch DG 2002 A quorum-sensing signaling system essential for genetic competence in *Streptococcus mutans* is involved in biofilm formation. J Bacteriol 184:2699–2708. doi:10.1128/JB.184.10.2699-2708.2002. 11976299 PMC135014

[53] Fontaine L, Goffin P, Dubout H, Delplace B, Baulard A, Lecat-Guillet N, Chambellon E, Gardan R, Hols P 2013 Mechanism of competence activation by the ComRS signalling system in streptococci. Mol Microbiol 87:1113–1132. doi:10.1111/mmi.12157. 23323845

[54] Mashburn-Warren L, Morrison DA, Federle MJ 2010 A novel double-tryptophan peptide pheromone controls competence in *Streptococcus* spp. via an Rgg regulator. Mol Microbiol 78:589–606. doi:10.1111/j.1365-2958.2010.07361.x. 20969646 PMC3058796

[55] Niu G, Okinaga T, Zhu L, Banas J, Qi F, Merritt J 2008 Characterization of *irvR*, a novel regulator of the *irvA*-dependent pathway required for genetic competence and dextran-dependent aggregation in *Streptococcus mutans*. J Bacteriol 190:7268–7274. doi:10.1128/JB.00967-08. 18757533 PMC2580701

[56] Zhu L, Lin J, Kuang Z, Vidal JE, Lau GW 2015 Deletion analysis of *Streptococcus pneumoniae* late competence genes distinguishes virulence determinants that are dependent or independent of competence induction. Mol Microbiol 97:151–165. doi:10.1111/mmi.13016. 25846124 PMC4536566

[57] Wenderska IB, Lukenda N, Cordova M, Magarvey N, Cvitkovitch DG, Senadheera DB 2012 A novel function for the competence inducing peptide, XIP, as a cell death effector of *Streptococcus mutans*. FEMS Microbiol Lett 336:104–112. doi:10.1111/j.1574-6968.2012.02660.x. 22900705 PMC4669055

[58] Palmer SR, Miller JH, Abranches J, Zeng L, Lefebure T, Richards VP, Lemos JA, Stanhope MJ, Burne RA 2013 Phenotypic heterogeneity of genomically-diverse isolates of *Streptococcus mutans*. PLoS One 8:e61358. doi:10.1371/journal.pone.0061358. 23613838 PMC3628994

[59] Yamashita Y, Bowen WH, Burne RA, Kuramitsu HK 1993 Role of the *Streptococcus mutans* *gtf* genes in caries induction in the specific-pathogen-free rat model. Infect Immun 61:3811–3817. 8359902 10.1128/iai.61.9.3811-3817.1993 PMC281081

[60] Culp DJ, Quivey RQ, Bowen WH, Fallon MA, Pearson SK, Faustoferri R 2005 A mouse caries model and evaluation of aqp5−/− knockout mice. Caries Res 39:448–454. doi:10.1159/000088179. 16251788

[61] Tanzer JM, Freedman ML, Fitzgerald RJ, Larson RH 1974 Diminished virulence of glucan synthesis-defective mutants of *Streptococcus mutans*. Infect Immun 10:197–203. 4842127 10.1128/iai.10.1.197-203.1974 PMC414977

[62] Abel S, Abel zur Wiesch P, Davis BM, Waldor MK 2015 Analysis of bottlenecks in experimental models of infection. PLoS Pathog 11:e1004823. doi:10.1371/journal.ppat.1004823. 26066486 PMC4465827

[63] Fujishima K, Kawada-Matsuo M, Oogai Y, Tokuda M, Torii M, Komatsuzawa H 2013 *dpr* and *sod* in *Streptococcus mutans* are involved in coexistence with *S. sanguinis*, and PerR is associated with resistance to H_2_O_2_. Appl Environ Microbiol 79:1436–1443. doi:10.1128/AEM.03306-12. 23263955 PMC3591952

[64] Durand A, Sinha AK, Dard-Dascot C, Michel B 2016 Mutations affecting potassium import restore the viability of the *Escherichia coli* DNA polymerase III *holD* mutant. PLoS Genet 12:e1006114. doi:10.1371/journal.pgen.1006114. 27280472 PMC4900610

[65] Langmead B, Salzberg SL 2012 Fast gapped-read alignment with Bowtie 2. Nat Methods 9:357–359. doi:10.1038/nmeth.1923. 22388286 PMC3322381

[66] Anders S, Pyl PT, Huber W 2015 HTSeq—a Python framework to work with high-throughput sequencing data. Bioinformatics 31:166–169. doi:10.1093/bioinformatics/btu638. 25260700 PMC4287950

[67] Sambrook J, Russell D 2001 Molecular cloning: a laboratory manual. Cold Spring Harbor Laboratory Press, Cold Spring Harbor, NY.

[68] Lau PC, Sung CK, Lee JH, Morrison DA, Cvitkovitch DG 2002 PCR ligation mutagenesis in transformable streptococci: application and efficiency. J Microbiol Methods 49:193–205. doi:10.1016/S0167-7012(01)00369-4. 11830305

[69] Ahn S-J, Burne RA 2006 The *atlA* operon of *Streptococcus mutans*: role in autolysin maturation and cell surface biogenesis. J Bacteriol 188:6877–6888. doi:10.1128/JB.00536-06. 16980491 PMC1595523

[70] Lang JM, Darling AE, Eisen JA 2013 Phylogeny of bacterial and archaeal genomes using conserved genes: supertrees and supermatrices. PLoS One 8:e62510. doi:10.1371/journal.pone.0062510. 23638103 PMC3636077

