# Supplementary figures and images for: Genomewide Identification of Essential Genes and Fitness Determinants of Streptococcus mutans UA159
Source: mSphere. 2018 Feb 7;3(1):e00031-18. doi: 10.1128/mSphere.00031-18 (PMC5806208; doi:10.1128/mSphere.00031-18)

**A**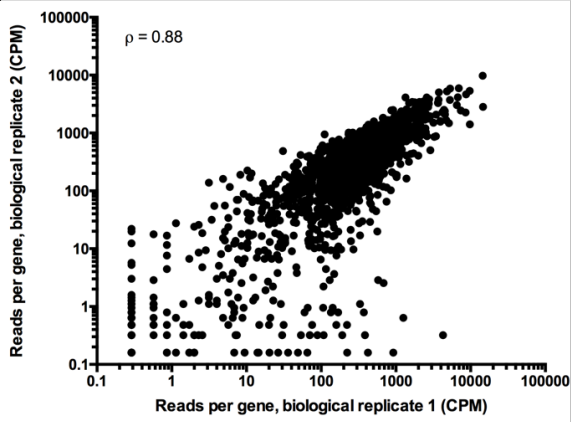**B**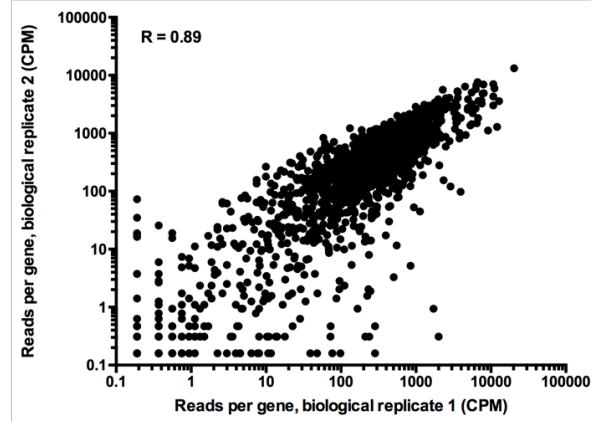**C**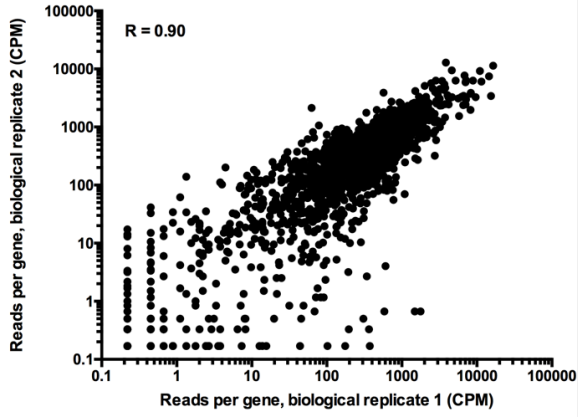**FIG S1**

Supplement: FIG S1 [file sph001182474sf1.pdf]

**A**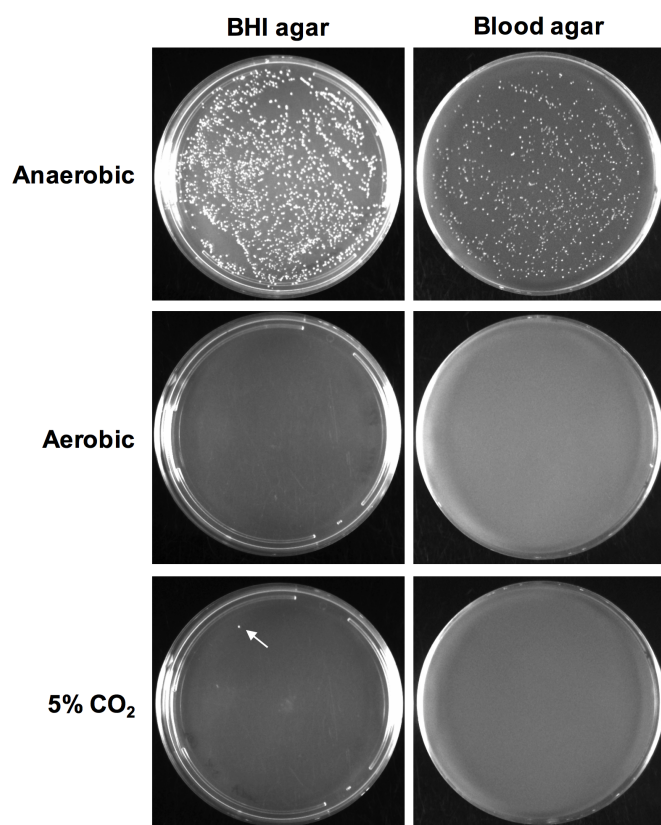**B**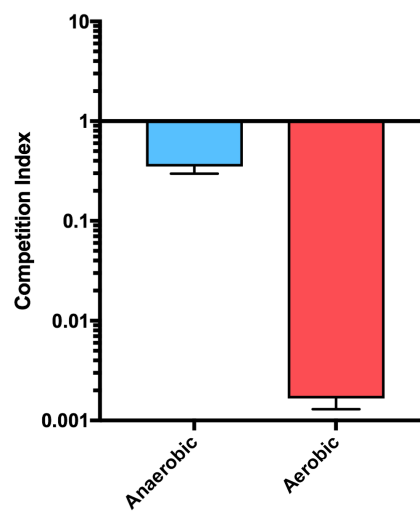**FIG S2**

Supplement: FIG S2 [file sph001182474sf2.pdf]
