# Supplementary material for: Genomewide Identification of Essential Genes and Fitness Determinants of Streptococcus mutans UA159
Source: mSphere. 2018 Feb 7;3(1):e00031-18. doi: 10.1128/mSphere.00031-18 (PMC5806208; doi:10.1128/mSphere.00031-18)
Supplement: TABLE S1 [file sph001182474st1.pdf]

Table S1

Locus	Description
Duplicated genes	
**SMU_1024c**	putative transposase fragment
**SMU_106c**	putative transposase fragment
**SMU_1347c**	conserved hypothetical protein; possible permease
**SMU_1348c**	putative ABC transporter, ATP-binding protein
**SMU_1365c**	hypothetical protein; possible permease
**SMU_1366c**	putative ABC transporter; ATP-binding protein
**SMU_1370c**	putative transposase, IS150-like
**SMU_1379**	Transposase and inactivated derivatives
**SMU_1380**	No description
**SMU_1407c**	putative transposase, ISSmu1
**SMU_1408c**	conserved hypothetical protein
**SMU_1505c**	hypothetical protein
**SMU_175**	hypothetical protein
**SMU_1893c**	putative transposase, ISSmu1
**SMU_1894c**	Transposase and inactivated derivatives
**SMU_1899**	putative ABC transporter, ATP-binding and permease protein (fragment)
**SMU_2003**	Ribosomal protein S13
**SMU_285**	hypothetical protein
**SMU_436c**	putative transposase, ISSmu1
**SMU_437c**	No description
**SMU_565c**	putative transposase, ISSmu1
**SMU_566c**	No description
**SMU_767**	putative transposase, ISSmu1
**SMU_875c**	putative transposase, IS150-like
**SMU_r07**	rRNA
**SMU_r08**	rRNA
**SMU_r09**	rRNA
**SMU_r10**	rRNA
**SMU_r11**	rRNA
**SMU_r12**	rRNA
**SMU_r13**	rRNA
**SMU_r14**	rRNA
**SMU_r15**	rRNA
**SMU_t43**	tRNA
**SMU_t45**	tRNA
**SMU_t47**	tRNA

**SMU_t49**	tRNA
**SMU_t53**	tRNA
**SMU_t55**	tRNA
**SMU_t57**	tRNA
**SMU_t59**	tRNA
Small genes	
**SMU_1231c**	hypothetical protein
**SMU_1808c**	putative integrase fragment
**SMU_2053c**	hypothetical protein
**SMU_68**	hypothetical protein
**SMU_t01**	tRNA
**SMU_t02**	tRNA
**SMU_t03**	tRNA
**SMU_t04**	tRNA
**SMU_t05**	tRNA
**SMU_t06**	tRNA
**SMU_t07**	tRNA
**SMU_t08**	tRNA
**SMU_t09**	tRNA
**SMU_t10**	tRNA
**SMU_t11**	tRNA
**SMU_t12**	tRNA
**SMU_t13**	tRNA
**SMU_t14**	tRNA
**SMU_t15**	tRNA
**SMU_t16**	tRNA
**SMU_t17**	tRNA
**SMU_t18**	tRNA
**SMU_t19**	tRNA
**SMU_t20**	tRNA
**SMU_t21**	tRNA
**SMU_t22**	tRNA
**SMU_t23**	tRNA
**SMU_t24**	tRNA
**SMU_t25**	tRNA
**SMU_t26**	tRNA
**SMU_t27**	tRNA
**SMU_t28**	tRNA
**SMU_t29**	tRNA
**SMU_t30**	tRNA

**SMU_t31**	tRNA
**SMU_t32**	tRNA
**SMU_t33**	tRNA
**SMU_t34**	tRNA
**SMU_t35**	tRNA
**SMU_t36**	tRNA
**SMU_t37**	tRNA
**SMU_t38**	tRNA
**SMU_t39**	tRNA
**SMU_t40**	tRNA
**SMU_t41**	tRNA
**SMU_t42**	tRNA
**SMU_t43**	tRNA
**SMU_t44**	tRNA
**SMU_t45**	tRNA
**SMU_t46**	tRNA
**SMU_t47**	tRNA
**SMU_t48**	tRNA
**SMU_t49**	tRNA
**SMU_t50**	tRNA
**SMU_t51**	tRNA
**SMU_t52**	tRNA
**SMU_t53**	tRNA
**SMU_t54**	tRNA
**SMU_t55**	tRNA
**SMU_t56**	tRNA
**SMU_t57**	tRNA
**SMU_t58**	tRNA
**SMU_t59**	tRNA
**SMU_t60**	tRNA
**SMU_t61**	tRNA
**SMU_t62**	tRNA
**SMU_t63**	tRNA
**SMU_t64**	tRNA
**SMU_t65**	tRNA
