# Supplementary material for: Genomewide Identification of Essential Genes and Fitness Determinants of Streptococcus mutans UA159
Source: mSphere. 2018 Feb 7;3(1):e00031-18. doi: 10.1128/mSphere.00031-18 (PMC5806208; doi:10.1128/mSphere.00031-18)
Supplement: TABLE S2 [file sph001182474st2.pdf]

Table S2

Locus	Gene	Product	Initial Ratio	BHI Ratio	FMC Ratio	Mouse Ratio
**SMU_01**	*dnaA*	chromosomal replication initiator protein DnaA	0.001	0.001	0.001	0.013
**SMU_02**	*dnaN*	DNA polymerase III, beta subunit	0.000	0.001	0.001	0.003
**SMU_05**	-	conserved hypothetical protein	0.136	0.079	0.058	0.000
**SMU_06**	-	uncharacterized GTP-binding protein	0.243	0.021	0.061	0.001
**SMU_07**	*pth*	peptidyl-tRNA hydrolase	0.000	0.001	0.000	0.001
**SMU_08**	*trcF*	transcription-repair coupling factor	1.671	1.577	1.274	0.008
**SMU_09**	-	small RNA binding protein	1.164	0.920	0.995	0.001
**SMU_10**	*divIC*	cell-division protein DivIC	1.703	0.153	0.042	0.005
**SMU_100**	*sorD*	sorbose PTS system, IIB component	1.800	0.828	1.276	0.002
**SMU_1000**	-	hypothetical protein	0.012	0.010	0.000	0.000
**SMU_1001**	*dprA*	DNA processing protein, Smf family	0.858	0.745	0.606	0.000
**SMU_1002**	*topA*	DNA topoisomerase I	0.035	0.052	0.041	0.001
**SMU_1003**	*gid*	glucose-inhibited division protein	0.424	0.312	0.419	0.000
**SMU_1004**	*gtfB*	glucosyltransferase-I	0.176	0.194	0.274	0.000
**SMU_1005**	*gtfC*	glucosyltransferase-SI	0.522	0.705	0.908	0.043
**SMU_1006**	*ysaC*	ABC transporter, ATP-binding protein	1.069	1.164	1.344	0.002
**SMU_1007**	*ysaB*	ABC transporter permease	1.819	2.018	2.158	0.066
**SMU_1008**	*llrG*	response regulator	1.312	1.322	1.326	0.014
**SMU_1009**	*kinG*	sensor histidine kinase	2.151	2.626	2.486	1.405
**SMU_101**	*sorC*	sorbose PTS system, IIC component	2.734	1.292	1.898	0.002
**SMU_1010**	*citC*	citrate lyase synthetase	1.140	1.234	1.154	0.125
**SMU_1011**	*citG*	citG protein	1.326	1.312	1.348	0.315
**SMU_1012c**	*cpsY*	transcriptional regulator	1.018	1.180	1.362	0.001
**SMU_1013c**	*yxiQ*	Mg2+/citrate complex transporter	1.819	1.918	2.193	7.316
**SMU_1014**	-	hypothetical protein	1.951	1.177	1.561	0.001
**SMU_1016**	*bcc*	biotin carboxyl carrier protein	0.441	0.841	0.561	0.000
**SMU_1017**	*oadB*	oxaloacetate decarboxylase, sodium ion pump subunit	1.090	1.139	0.806	0.001
**SMU_1018**	-	hypothetical protein	0.968	0.964	1.200	0.346
**SMU_1019**	*cilG*	citrate lyase, gamma subunit	0.263	0.212	0.200	0.000
**SMU_102**	*sorB*	PTS system, IID component	3.688	2.776	5.002	3.878
**SMU_1020**	*cilB*	citrate lyase, beta subunit	0.871	0.892	0.420	0.001
**SMU_1021**	*cilA*	citrate lyase alpha chain	0.721	0.714	0.336	0.000
**SMU_1022**	*citG2*	conserved hypothetical protein (possible CitXG protein)	0.463	0.425	0.282	0.001
**SMU_1023**	*pycB*	pyruvate carboxylase/oxaloacetate decarboxylase, alpha subunit	0.894	0.904	0.759	0.015
**SMU_1025**	*rmaA*	transcriptional regulator	0.633	0.576	0.486	0.036
**SMU_1026**	-	conserved hypothetical protein	2.686	3.053	2.669	0.001
**SMU_1027**	-	transcriptional regulator (TetR/AcrR family)	2.778	3.330	3.839	0.483

**SMU_1028**	-	hydrolase or acyltransferase	1.330	1.553	1.592	0.012
**SMU_1029**	-	hypothetical - transposon	0.085	0.059	0.093	0.000
**SMU_103**	*sorA*	PTS system, IIA component	6.562	5.449	5.922	0.001
**SMU_1030**	-	polyribonucleotide nucleotidyltransferase	0.485	0.579	0.435	0.000
**SMU_1031**	*xis*	transposon excisionase TN916 orf1-like	1.049	0.987	1.169	0.000
**SMU_1032**	*tnr5*	transposon/phage-related integrase	0.976	1.127	1.156	0.000
**SMU_1034 c**	*xerC*	conserved hypothetical protein (possible integrase/recombinase)	0.551	0.093	0.104	0.001
**SMU_1035**	*glrA*	ABC transporter ATP-binding protein homolog	2.142	1.861	1.612	0.004
**SMU_1036**	-	conserved hypothetical protein	2.644	2.932	2.757	0.002
**SMU_1037 c**	*phoR*	histidine kinase	1.021	1.209	1.187	0.003
**SMU_1038 c**	*ycbL*	response regulator	0.731	0.938	0.864	0.001
**SMU_1039 c**	*kdt*	UDP-glucose:(glucosyl) LPS alpha1,2-glucosyltransferase	1.325	1.643	1.874	0.001
**SMU_104**	*yicI*	glycosyl hydrolase, alpha-glucosidase	3.595	2.558	1.371	0.043
**SMU_1040 c**	*ydfG*	oxidoreductase, short-chain dehydrogenase/reductase	1.370	1.640	1.947	0.000
**SMU_1041**	*ecsA*	ABC transporter, ATP-binding protein	0.417	0.303	0.370	0.001
**SMU_1042**	-	conserved hypothetical protein; possible ABC transporter, permease	0.917	0.808	0.805	0.001
**SMU_1043 c**	*pta*	phosphate acetyltransferase	0.237	0.082	0.005	0.001
**SMU_1044 c**	*rluE*	ribosomal large subunit pseudouridine synthase	1.668	0.868	0.867	0.073
**SMU_1045 c**	*ppnK*	NAD(+) kinase (ATP-NAD kinase)	0.000	0.000	0.000	0.000
**SMU_1046 c**	*relA*	GTP pyrophosphokinase	0.220	0.111	0.226	0.000
**SMU_1047 c**	-	hypothetical protein	1.557	1.558	2.173	0.003
**SMU_1048**	-	conserved hypothetical protein	1.041	1.079	0.917	0.000
**SMU_105**	*scrR*	SCR operon transcriptional repressor	2.707	1.503	0.921	0.001
**SMU_1050**	*krpS*	phosphoribosyl pyrophosphate synthetase	0.139	0.029	0.010	0.000
**SMU_1051**	*nifS*	aminotransferase (class V); possible iron-sulfur cofactor synthesis protein;pyridoxal-phosphate dependent aminotransferase	0.000	0.001	0.001	0.008
**SMU_1052**	-	conserved hypothetical protein	0.242	0.278	0.297	0.000
**SMU_1053**	*rex*	possible redox-sensing transcriptional repressor Rex	0.371	0.217	0.082	0.008
**SMU_1054**	*guaA*	glutamine amidotransferase	0.521	0.265	0.512	0.001
**SMU_1055**	*radC*	DNA repair protein RadC	0.723	0.674	0.994	0.000
**SMU_1056**	*radC*	DNA repair protein RadC	2.579	2.442	2.894	0.000

**SMU_1057**	*satE*	acid tolerance protein	1.457	1.312	1.655	0.003
**SMU_1058**	*satD*	acid tolerance protein	0.406	0.206	0.213	0.000
**SMU_1059**	*satC*	acid tolerance protein	3.843	2.204	1.217	0.001
**SMU_1060**	*ffh*	signal recognition particle	0.122	0.003	0.007	0.001
**SMU_1061**	*ylxM*	DNA-binding protein	1.151	0.358	0.042	0.042
**SMU_1062**	*opuAb*	glycine-betaine binding ABC transporter permease	0.576	0.358	0.350	0.001
**SMU_1063**	*opuAa*	amino acid ABC transporter, ATP-binding protein	0.652	0.573	0.725	0.330
**SMU_1064c**	*busR*	transcriptional regulator	0.977	0.595	0.437	0.001
**SMU_1065c**	*rgrB*	transcriptional regulator, GntR family	1.526	0.983	0.671	0.000
**SMU_1066**	*guaA*	GMP synthase	0.072	0.001	0.060	0.001
**SMU_1067c**	-	ABC transporter, permease protein	2.141	1.924	2.259	0.006
**SMU_1068c**	-	ABC transporter, ATP-binding protein	1.233	1.380	1.263	0.002
**SMU_1069c**	-	hypothetical protein	2.419	1.347	2.821	0.009
**SMU_107**	-	hypothetical protein	1.880	2.365	3.892	0.022
**SMU_1070c**	-	conserved hypothetical protein	0.579	0.737	0.933	0.000
**SMU_1071c**	-	conserved hypothetical protein	1.662	1.649	1.496	0.003
**SMU_1072c**	*bar*	acyltransferase	0.761	0.941	0.972	0.001
**SMU_1073**	*fthS*	formate--tetrahydrofolate ligase	0.368	0.133	0.000	0.000
**SMU_1074**	*dfpB*	flavoprotein	0.034	0.017	0.026	0.000
**SMU_1075**	*dfp*	DNA/pantothenate metabolism flavoprotein	0.001	0.000	0.000	0.000
**SMU_1076**	-	conserved hypothetical protein (possible membrane protein)	1.156	1.685	0.028	0.002
**SMU_1077**	*pgm*	phosphoglucomutase	2.338	0.001	0.007	0.001
**SMU_1078c**	-	multidrug ABC transporter, ATP-binding and permease protein	0.777	1.025	0.654	0.019
**SMU_1079c**	-	ABC transporter, ATP-binding / permease protein	0.702	1.152	0.327	0.001
**SMU_108**	-	hypothetical protein	6.312	5.005	9.509	0.001
**SMU_1080c**	-	conserved hypothetical protein	1.789	1.214	0.040	0.001
**SMU_1081c**	-	conserved hypothetical protein	0.413	0.121	0.105	0.004
**SMU_1082**	*glyA*	serine hydroxymethyltransferase	0.536	0.300	0.143	0.007
**SMU_1083c**	-	conserved hypothetical protein	0.037	0.000	0.000	0.000
**SMU_1084**	*hemK*	protoporphyrinogen oxidase	0.132	0.016	0.001	0.001
**SMU_1085**	*rf1*	peptide chain release factor 1	0.000	0.000	0.000	0.002
**SMU_1086**	*kitH*	thymidine kinase	0.176	0.143	0.143	0.000
**SMU_1087**	*xylH*	4-oxalocrotonate tautomerase	1.198	1.415	2.063	0.001
**SMU_1088**	*apbE*	thiamine biosynthesis lipoprotein	0.251	0.253	0.217	0.002
**SMU_1089**	-	conserved hypothetical protein	0.598	0.448	0.603	0.096
**SMU_109**	-	lantibiotic related antibiotic efflux protein/macrolide permease	5.679	7.863	10.357	19.644
**SMU_1090**	-	conserved hypothetical protein	0.581	0.537	0.564	0.000

**SMU_1091**	*wapE*	hypothetical protein (possible cell wall protein)	0.817	0.722	0.777	0.003
**SMU_1093**	*vex3*	ABC transporter permease protein	0.856	0.890	0.901	0.002
**SMU_1094**	*vex2*	ABC transporter, ATP-binding protein	0.668	0.648	0.771	0.000
**SMU_1095**	*opuBc*	proline/glycine betaine ABC permease and solute binding protein	0.644	0.598	0.650	0.001
**SMU_1096**	*opuBa*	ABC transporter, ATP-binding protein	0.551	0.591	0.531	0.000
**SMU_1097c**	-	transcriptional regulator	0.575	0.612	0.665	0.003
**SMU_1098c**	*yogA*	oxidoreductase	1.194	1.155	1.425	0.002
**SMU_11**	-	Streptococcus-specific protein	0.087	0.126	0.144	0.007
**SMU_110**	*mutR*	transcriptional regulator	6.142	7.638	9.753	0.496
**SMU_1100c**	-	permease	0.936	1.300	1.409	0.001
**SMU_1102**	*ascB*	6-phospho-beta-glucosidase, required for arbutin uptake and metabolism	1.350	1.472	1.615	0.000
**SMU_1104c**	*gpm*	phosphoglycerate mutase-like protein	1.417	1.606	1.660	0.000
**SMU_1105c**	*gpm*	phosphoglycerate mutase-like protein	0.386	0.618	0.483	0.005
**SMU_1106c**	*gpm*	phosphoglycerate mutase-like protein	1.038	1.133	1.487	0.001
**SMU_1107c**	-	conserved hypothetical protein	1.836	1.740	1.854	0.406
**SMU_1108c**	-	hydrolase	0.654	0.862	0.838	0.000
**SMU_1109c**	-	conserved hypothetical protein	1.056	1.412	1.976	0.931
**SMU_1111c**	-	conserved hypothetical protein	1.012	0.747	0.817	0.001
**SMU_1112c**	-	conserved hypothetical protein	0.366	0.747	0.439	0.000
**SMU_1113**	*srtA*	sortase	0.812	1.563	2.833	0.000
**SMU_1114**	*gyrA*	DNA gyrase A subunit	0.003	0.000	0.001	0.003
**SMU_1115**	*ldh*	L-lactate dehydrogenase	0.000	0.001	0.001	0.011
**SMU_1116c**	-	hypothetical protein	1.046	1.044	0.996	0.014
**SMU_1117**	*nox*	H2O-forming NADH Oxidase	0.533	0.428	0.491	0.001
**SMU_1118c**	*rnsD*	ribonucleoside ABC transporter permease	1.206	0.958	0.788	0.002
**SMU_1119c**	*rnsC*	ribonucleoside ABC transporter permease	0.861	0.804	0.534	0.000
**SMU_1120**	*rnsA*	ribonucleoside ABC transporter, ATP-binding protein	0.922	0.739	0.604	0.002
**SMU_1121c**	*rnsB*	ribonucleoside ABC transporter, solute-binding protein	0.640	0.694	0.424	0.000
**SMU_1122**	*cdd*	cytidine deaminase	0.843	0.660	0.742	0.001
**SMU_1123**	*deoC*	deoxyribose-phosphate aldolase	0.957	0.557	0.398	0.000
**SMU_1124**	*pdp*	pyrimidine-nucleoside phosphorylase	1.017	0.839	0.762	0.002

**SMU_1125c**	-	conserved hypothetical protein	1.030	0.918	0.936	0.002
**SMU_1126**	*coaA*	pantothenate kinase	0.003	0.001	0.000	0.004
**SMU_1127**	*rs20*	30S ribosomal protein S20	2.062	2.142	2.815	0.000
**SMU_1128**	*ciaH*	two-component sensor histidine kinase	0.884	1.083	0.457	0.001
**SMU_1129**	*ciaR*	two-component response regulator	0.384	0.366	0.324	0.001
**SMU_112c**	-	transcriptional regulator, RpiR family	2.382	2.501	3.080	0.001
**SMU_113**	*pfk*	fructose-1-phosphate kinase	1.607	1.745	2.031	0.001
**SMU_1131c**	-	hypothetical protein	0.485	0.811	0.708	0.000
**SMU_1132**	*pepN*	lysyl-aminopeptidase	0.307	0.228	0.308	0.002
**SMU_1133**	*phoU*	phosphate uptake regulatory protein	0.230	0.321	0.048	0.001
**SMU_1134c**	*pstB*	phosphate ABC transporter, ATP-binding protein	0.011	0.001	0.001	0.000
**SMU_1135**	*pstB*	phosphate ABC transporter, ATP-binding protein	0.030	0.002	0.005	0.002
**SMU_1136**	*pstC*	phosphate ABC transporter, permease	0.001	0.003	0.000	0.002
**SMU_1137**	*pstC1*	phosphate ABC transporter, permease	0.007	0.002	0.001	0.002
**SMU_1138**	*pstS*	phosphate ABC transporter, periplasmic phosphate-binding protein	0.001	0.001	0.000	0.000
**SMU_1139c**	*yebU*	rRNA methyltransferase, NOL1/NOP2/sun family	0.593	0.245	0.323	0.009
**SMU_114**	-	PTS system, fructose-specific IIBC component	1.957	2.383	2.472	0.001
**SMU_1140c**	-	conserved hypothetical protein (possible myo-inositol-1(or 4)-monophosphatase)	0.850	0.425	0.533	0.000
**SMU_1141c**	-	conserved hypothetical protein	0.345	0.066	0.093	0.033
**SMU_1142c**	-	conserved hypothetical protein (possible arsenate reductase)	0.184	0.036	0.026	0.006
**SMU_1143c**	*mreA*	bifunctional protein: riboflavin kinase/flavin adenine dinucleotide (FAD) synthase	0.009	0.014	0.016	0.003
**SMU_1144**	*truB*	tRNA pseudouridine synthase B	0.486	0.123	0.149	0.001
**SMU_1145c**	*covS*	histidine kinase related to CovS	1.005	1.106	0.900	0.001
**SMU_1146c**	*covR*	response regulator related to CovR	1.705	1.460	1.371	0.006
**SMU_1147c**	-	hypothetical protein	0.884	0.925	1.039	0.002
**SMU_1148**	*lctF*	ABC transporter, ATPase component	0.666	0.803	0.796	0.000
**SMU_1149**	*lctE*	ABC transporter, membrane spanning	1.190	1.181	1.298	0.000
**SMU_115**	-	PTS system, fructose-specific IIA component	2.853	3.318	3.368	0.000

**SMU_1150**	*lctG*	ABC transporter, membrane spanning	1.032	0.870	0.797	16.724
**SMU_1151c**	-	conserved hypothetical protein	0.899	0.934	0.866	0.001
**SMU_1152c**	-	conserved hypothetical protein	0.660	2.464	1.675	0.000
**SMU_1153c**	-	conserved hypothetical protein	1.879	1.902	2.050	0.001
**SMU_1154c**	-	conserved hypothetical protein	1.492	0.822	0.663	0.000
**SMU_1155**	-	hypothetical protein	2.051	1.520	1.520	0.001
**SMU_1156c**	-	hypothetical protein	4.442	2.860	2.447	0.001
**SMU_1157c**	-	conserved hypothetical protein	3.864	3.694	4.233	0.002
**SMU_1158c**	-	conserved hypothetical protein	1.195	0.936	0.649	0.002
**SMU_1159c**	-	hypothetical protein	0.410	0.560	0.397	0.000
**SMU_116**	*lacD*	tagatose 1,6-aldolase	3.361	3.371	3.535	0.001
**SMU_1160c**	-	hypothetical protein	3.035	2.798	3.029	0.002
**SMU_1161c**	-	conserved hypothetical protein	2.590	1.706	1.470	0.001
**SMU_1163c**	-	ABC transporter, ATPase component	1.079	1.191	1.267	0.001
**SMU_1164c**	-	ABC transporter, ATPase component	1.478	1.474	1.370	0.001
**SMU_1165c**	*acrR*	transcriptional regulator (TetR/AcrR family)	0.871	0.949	0.844	0.000
**SMU_1166c**	*psaB*	ABC transporter permease	1.009	1.162	1.239	0.002
**SMU_1167c**	*psaA*	ABC transporter ATP-binding protein	0.935	1.217	2.776	0.001
**SMU_1168**	*psaR*	transcriptional regulator (TetR/AcrR family)	2.054	2.301	1.994	0.008
**SMU_1169c**	-	thioredoxin family protein	1.186	0.936	0.882	0.001
**SMU_1170**	*ccdA*	cytochrome c-type biogenesis protein	0.768	0.907	0.947	0.001
**SMU_1171c**	-	conserved hypothetical protein	1.083	1.014	0.758	0.000
**SMU_1172c**	-	conserved hypothetical protein	1.090	0.713	0.678	0.001
**SMU_1173**	*cysD*	O-acetylhomoserine sulfhydrylase	0.898	0.462	0.543	0.009
**SMU_1174**	*pcrA*	ATP-dependent DNA helicase	0.493	0.046	0.012	0.000
**SMU_1175**	*dagA*	sodium:alanine (or glycine) symporter	0.748	0.446	0.838	0.002
**SMU_1176**	*ydaE*	cation efflux protein	1.636	0.890	0.136	0.497
**SMU_1177c**	*aatB*	amino acid ABC transporter, amino acid-binding protein	0.360	0.033	0.354	0.001
**SMU_1178c**	*msmK*	amino acid ABC transporter, ATP-binding protein	0.760	0.059	0.388	0.001
**SMU_1179c**	*glnP*	amino acid ABC transporter, permease	0.926	0.190	0.780	0.006
**SMU_117c**	-	conserved hypothetical protein	4.029	4.600	4.757	1.644

**SMU_1180**	*phnA*	alkylphosphonate uptake protein	0.453	0.769	2.085	0.002
**SMU_1182**	*mtlD*	mannitol 1-phosphate 5-dehydrogenase	0.415	0.234	0.576	0.001
**SMU_1183**	*mtlA2*	phosphotransferase system enzyme II	0.576	0.272	0.244	0.001
**SMU_1184c**	-	transcriptional regulator	0.728	0.994	1.028	0.001
**SMU_1185**	*mtlA1*	mannitol PTS EII	0.609	1.327	0.983	0.007
**SMU_1187**	*glmS*	L-glutamine-D-fructose-6-phosphate amidotransferase	0.000	0.000	0.001	0.002
**SMU_1188**	*lepB*	signal peptidase I	1.379	1.004	1.720	0.001
**SMU_1189c**	-	conserved hypothetical protein	1.419	1.631	1.817	0.008
**SMU_118c**	-	esterase	3.076	3.843	4.097	0.001
**SMU_119**	*adh*	alcohol dehydrogenase class III	3.045	3.104	2.648	0.328
**SMU_1190**	*pykF*	pyruvate kinase	0.004	0.001	0.002	0.005
**SMU_1191**	*pfk*	6-phosphofructokinase	0.002	0.000	0.000	0.000
**SMU_1192**	*dnaE*	DNA-polymerase III subunit alpha	0.002	0.002	0.003	0.005
**SMU_1193**	*yhcF*	transcriptional regulator, GntR family	0.407	0.045	0.047	0.618
**SMU_1194**	*yurY*	ABC transporter, ATP-binding protein	0.798	0.775	1.161	0.002
**SMU_1195**	-	ABC transporter permease protein	1.311	1.186	1.994	0.152
**SMU_1196c**	-	conserved hypothetical protein	0.937	0.731	0.948	0.000
**SMU_1197**	-	conserved hypothetical protein	0.646	0.537	0.962	0.081
**SMU_12**	-	conserved hypothetical protein	2.204	3.620	3.910	0.003
**SMU_120**	*rl28*	50S ribosomal protein L28	0.487	0.337	0.499	0.009
**SMU_1200**	*rs1*	30S ribosomal protein S1	0.039	0.010	0.040	0.001
**SMU_1201c**	-	conserved hypothetical protein	0.000	0.000	0.000	0.000
**SMU_1203**	*ilvE*	branched-chain amino acid aminotransferase	0.457	0.009	0.000	0.001
**SMU_1204**	*parC*	DNA topoisomerase IV subunit A	0.001	0.001	0.001	0.014
**SMU_1205c**	-	hypothetical protein	2.227	2.298	2.638	0.000
**SMU_1206c**	-	conserved hypothetical protein	0.198	0.187	0.137	0.002
**SMU_1207**	*fic*	cell filamentation / mobilization protein	0.579	0.232	0.215	0.000
**SMU_1208c**	-	hypothetical protein	0.233	0.162	0.183	0.109
**SMU_1209c**	-	hypothetical protein	0.677	0.454	0.651	0.001
**SMU_121**	*dinF*	MATE efflux family , DinF	1.984	2.011	1.798	0.001
**SMU_1210**	*parE*	DNA topoisomerase IV subunit B	0.001	0.000	0.000	0.001
**SMU_1211**	-	conserved hypothetical protein	0.008	0.001	0.001	0.002
**SMU_1213c**	*cpdB*	5'-nucleotidase/2',3'-cyclic-nucleotide 2'-phosphodiesterase	1.152	0.890	0.632	0.021
**SMU_1214**	*pyrC*	dihydroorotase	0.942	0.648	0.058	0.005
**SMU_1215**	*ung*	uracil-DNA glycosylase	0.726	0.530	0.197	0.002
**SMU_1216c**	-	ABC transporter, amino acid permease	1.843	1.564	1.395	1.285

**SMU_1217c**	*atmA*	amino acid ABC transporter, substrate binding protein	0.495	0.438	0.432	0.004
**SMU_1218**	*nylA*	Gln-dependent amidotransferase, subunit A	1.322	1.005	0.736	0.144
**SMU_1219c**	-	conserved hypothetical protein	1.181	1.230	0.537	0.880
**SMU_1220c**	-	conserved hypothetical protein	1.730	1.695	0.904	0.000
**SMU_1221**	*pyrE*	orotate phosphoribosyltransferase	0.913	0.896	0.035	0.019
**SMU_1222**	*pyrF*	orotidine 5'-phosphate decarboxylase	1.351	0.777	0.015	0.000
**SMU_1223**	*pyrDB*	dihydroorotate dehydrogenase	1.144	0.892	0.069	0.000
**SMU_1224**	*pyrK*	dihydroorotate dehydrogenase electron transfer subunit	1.488	1.337	0.123	0.000
**SMU_1225**	*cpsY*	transcriptional regulator, LysR family; probable metCcysK operon transcriptional activator	1.294	1.331	0.894	0.002
**SMU_1226c**	-	histidine protein kinase	1.074	0.761	0.892	0.001
**SMU_1227**	*deoD*	purine-nucleotide phosphorylase	0.690	0.477	0.565	0.003
**SMU_1228c**	-	glutamine amidotransferase	1.211	1.325	1.231	0.001
**SMU_1229**	*punA*	purine nucleoside phosphorylase	0.741	0.673	0.614	0.001
**SMU_123**	*polC*	DNA polymerase III, alpha subunit	0.006	0.004	0.002	0.001
**SMU_1230c**	-	conserved hypothetical protein	2.561	1.841	2.446	0.002
**SMU_1232c**	-	conserved hypothetical protein	0.981	0.554	0.389	0.001
**SMU_1233**	*deoB*	phosphopentomutase	1.487	0.362	0.952	0.001
**SMU_1234**	*rpiA*	ribose 5-phosphate isomerase A	0.098	0.030	0.014	0.001
**SMU_1235**	*thdF*	thiophene and furan oxidation protein; tRNA modification GTPase	0.244	0.014	0.010	0.000
**SMU_1236c**	-	conserved hypothetical protein (possible dihydroorotase family)	0.781	0.653	0.880	0.001
**SMU_1237c**	-	hypothetical protein	1.111	1.674	2.192	0.000
**SMU_1238c**	-	conserved hypothetical protein	0.803	1.549	0.472	0.001
**SMU_1239**	*pepV*	dipeptidase	0.565	0.387	0.629	0.000
**SMU_124**	*ykoM*	transcriptional regulator, MarR family	1.352	0.458	1.091	0.002
**SMU_1240c**	*nrd*	nitroreductase family protein	0.523	0.382	0.515	0.000
**SMU_1241**	*uvrC*	excinuclease ABC, subunit C	0.860	0.478	0.460	0.000
**SMU_1243**	*ltrA*	conserved hypothetical protein (possible low temperature requirement protein A)	1.784	1.406	1.331	0.001
**SMU_1245c**	-	hydrolase, haloacid dehalogenase-like family	0.776	0.535	0.788	0.000
**SMU_1246c**	-	transcriptional regulator (TetR/AcrR family)	0.837	0.667	0.769	0.001
**SMU_1247**	*eno*	enolase	0.000	0.001	0.000	0.000

**SMU_1249c**	-	hypothetical protein	1.765	1.690	1.242	0.001
**SMU_125**	-	conserved hypothetical protein	3.892	2.291	1.735	0.007
**SMU_1250c**	-	hypothetical protein	1.403	1.604	1.229	0.731
**SMU_1251**	-	conserved hypothetical protein	0.935	1.253	0.995	0.001
**SMU_1252**	*cgk*	glycerate kinase	1.271	1.248	1.329	0.043
**SMU_1253c**	-	hypothetical protein	1.520	1.526	1.188	0.003
**SMU_1254**	-	phosphatase	0.801	0.669	0.757	0.000
**SMU_1255c**	-	hypothetical protein	0.997	1.119	1.306	0.000
**SMU_1256c**	-	hypothetical protein	2.174	0.554	0.397	0.000
**SMU_1257c**	-	conserved hypothetical protein	0.978	1.215	1.002	0.000
**SMU_1258c**	-	type II restriction endonuclease subunit	1.566	1.480	1.829	0.182
**SMU_1259**	-	restriction endonuclease	3.446	3.919	3.337	0.000
**SMU_1260c**	-	conserved hypothetical protein	1.536	1.146	1.428	3.568
**SMU_1261c**	*hisE*	phosphoribosyl-AMP cyclohydrolase/phosphoribosyl-ATP pyrophosphohydrolase	1.135	0.663	0.820	0.000
**SMU_1262c**	-	hypothetical protein	8.286	7.221	8.554	0.002
**SMU_1263**	*hisI*	phosphoribosyl-AMP cyclohydrolase/phosphoribosyl-ATP pyrophosphatase	2.154	0.690	1.023	2.273
**SMU_1264**	*hisF*	cyclase HisF/ imidazoleglycerol-phosphate synthase	1.269	0.434	0.509	0.000
**SMU_1265**	*hisA*	phosphoribosylformimino-5-aminoimidazole carboxamide ribotide isomerase	1.135	0.325	0.412	0.000
**SMU_1266**	*hisH*	amidotransferase; possible imidazoleglycerol-phosphate synthase	0.325	0.074	0.096	0.002
**SMU_1267c**	-	hypothetical protein	1.230	0.463	0.279	0.004
**SMU_1268**	*hisB*	imidazoleglycerol-phosphate dehydratase	0.545	0.153	0.224	0.177
**SMU_1269**	*serB*	phosphoserine phosphatase	0.272	0.042	0.030	0.000
**SMU_127**	*adhA*	acetoin dehydrogenase (TPP-dependent) E1 component alpha subunit	2.021	0.540	1.703	0.000
**SMU_1270**	*hisD*	histidinol dehydrogenase	0.467	0.068	0.086	0.000
**SMU_1271**	*hisG*	ATP phosphoribosyltransferase	0.674	0.125	0.163	0.000
**SMU_1272**	*hisZ*	histidyl-tRNA synthetase	0.981	0.226	0.299	0.000
**SMU_1273**	*hisC*	histidinol-phosphate aminotransferase	0.886	0.130	0.264	0.000
**SMU_1276c**	*ezrA*	cell division regulator	0.138	0.017	0.022	0.001
**SMU_1277**	*gyrB*	DNA gyrase subunit B	0.001	0.000	0.000	0.000
**SMU_1278c**	*gph*	phosphoglycolate phosphatase	0.199	0.050	0.074	0.001
**SMU_1279c**	*rodA*	cell division protein; shape-determining protein	2.516	7.086	15.449	68.086

**SMU_128**	*adhB*	acetoin dehydrogenase (TPP-dependent) E1 component beta subunit	1.067	0.423	1.230	0.002
**SMU_1280c**	-	conserved hypothetical protein (possible alpha/beta superfamily hydrolase)	1.077	0.786	0.711	0.001
**SMU_1282**	*psaR*	transcriptional regulator, AcrR family	2.046	1.870	1.974	0.153
**SMU_1284c**	-	conserved hypothetical protein	1.076	0.823	0.830	0.000
**SMU_1286c**	*blt*	multidrug resistance permease	2.340	1.750	1.931	0.002
**SMU_1287**	*tnrA*	transcriptional regulator	1.715	1.181	1.179	0.002
**SMU_1288**	*rl19*	50S ribosomal protein L19	0.138	0.026	0.055	0.000
**SMU_1289c**	-	voltage-gated chloride channel family	1.055	0.651	1.072	0.001
**SMU_129**	*adhC*	dihydrolipoamide S-acetyltransferase	1.105	0.281	0.621	0.000
**SMU_1290c**	-	conserved hypothetical protein	1.226	0.966	1.229	0.002
**SMU_1291c**	*pheA*	chorismate mutase	0.046	0.050	0.126	0.001
**SMU_1292c**	-	conserved hypothetical protein	2.108	1.168	1.737	0.000
**SMU_1293c**	-	conserved hypothetical protein	0.808	0.530	0.729	0.006
**SMU_1294**	*flaW*	flavodoxin	0.000	0.002	0.001	0.001
**SMU_1295**	*add*	adenosine deaminase	0.463	0.031	0.500	0.000
**SMU_1296**	*yghU*	glutathione S-transferase	0.862	1.167	1.080	0.000
**SMU_1297**	-	conserved hypothetical protein, DHH family	0.589	0.022	0.000	0.000
**SMU_1298**	*rl31*	50S ribosomal protein L31	0.028	0.000	0.000	0.000
**SMU_1299c**	-	methyltransferase, possible acetate kinase	0.768	0.863	0.755	0.000
**SMU_13**	-	cell-cycle protein, PP-loop superfamily	0.002	0.012	0.013	0.003
**SMU_130**	*adhD*	dihydrolipoamide dehydrogenase	1.119	0.391	0.892	0.001
**SMU_1300c**	-	conserved hypothetical protein	0.630	0.779	0.748	0.010
**SMU_1301c**	-	methyltransferase related segment	1.420	2.041	1.787	0.001
**SMU_1302**	*adcA*	zinc ABC transporter, substrate-binding protein	0.642	0.598	0.783	0.001
**SMU_1303c**	*pepD*	dipeptidase	1.274	0.722	0.541	0.000
**SMU_1304c**	-	conserved hypothetical protein	1.303	0.514	0.492	0.000
**SMU_1305c**	-	conserved hypothetical protein	1.662	1.971	1.276	0.000
**SMU_1306c**	-	conserved hypothetical protein, ATP-binding domain	0.474	0.187	0.182	0.008
**SMU_1307c**	-	conserved hypothetical protein	0.821	0.614	0.786	0.001
**SMU_1308**	*aldR*	AldR-related regulatory protein: translation initiation inhibitor	0.650	0.412	0.519	0.001
**SMU_1309c**	*gldA*	glycerol dehydrogenase	0.684	0.368	0.407	0.000

**SMU_131**	*lplA*	lipoate-protein ligase	2.391	0.555	1.349	0.000
**SMU_1310**	-	hypothetical protein	1.370	0.560	1.164	0.001
**SMU_1311**	*asnS*	asparaginyl-tRNA synthetase	0.654	0.457	0.502	0.001
**SMU_1312**	*aspB*	aspartate aminotransferase	0.213	0.016	0.015	0.002
**SMU_1313c**	*dinG*	ATP-dependent DNA helicase	0.999	0.842	0.806	0.003
**SMU_1314**	-	conserved hypothetical protein	2.685	1.735	1.480	15.461
**SMU_1315c**	-	ABC transporter, ATP-binding protein	2.202	2.138	1.916	0.001
**SMU_1316c**	-	hypothetical protein	2.070	1.939	2.673	0.003
**SMU_1317c**	-	hypothetical protein	3.857	3.806	4.416	0.026
**SMU_1319c**	-	conserved hypothetical protein	0.718	0.892	0.776	0.005
**SMU_132**	*hipO*	amino acid amidohydrolase (hippurate amidohydrolase)	1.072	0.936	1.091	0.835
**SMU_1321c**	-	conserved hypothetical protein	1.557	1.955	1.713	2.747
**SMU_1322**	*budC*	acetoin reductase	0.765	0.794	0.667	0.000
**SMU_1323**	-	conserved hypothetical protein (possible hydrolase)	0.986	0.921	0.941	0.000
**SMU_1324**	*ftsX*	cell division ABC transporter, permease	0.193	0.001	0.001	0.001
**SMU_1325**	*ftsE*	cell-division ABC transporter, ATP-binding protein	0.165	0.000	0.001	0.000
**SMU_1326**	*rf2*	peptide chain release factor	0.001	0.000	0.001	0.001
**SMU_1327c**	-	conserved hypothetical protein, 4Fe-4S binding domain	0.941	0.952	1.071	0.001
**SMU_1329c**	*paaB*	transposase fragment	1.652	1.036	0.995	0.000
**SMU_1330c**	*tpn*	transposase, IS1167, fragment	0.302	0.351	0.223	0.000
**SMU_1331c**	*tpn*	transposase	0.849	0.736	0.724	0.001
**SMU_1332c**	*tpn*	transposase	1.012	0.843	0.591	0.000
**SMU_1334**	*sfp*	phosphopantetheinyl transferase	4.529	4.344	4.590	0.001
**SMU_1335c**	*fabK*	enoyl-acyl carrier protein(ACP) reductase; dioxygenase related to 2-nitropropane dioxygenase	3.653	3.440	3.202	0.040
**SMU_1336**	*pksD*	conserved hypothetical protein	1.815	1.742	1.641	0.382
**SMU_1337c**	-	alpha/beta superfamily hydrolases	4.311	4.662	5.209	0.008
**SMU_1338c**	*mefE*	ABC transport macrolide permease	4.492	2.753	2.826	4.022
**SMU_1339**	*bacD*	bacitracin synthetase; surfactin synthetase	2.479	2.885	3.143	1.188
**SMU_133c**	-	carbohydrate permease; transmembrane efflux protein	1.788	2.210	2.287	0.001
**SMU_134**	-	transcriptional regulator, TetR/AcrR family	2.069	0.758	0.109	0.013
**SMU_1340**	*bacA2*	bacitracin synthetase 1/ tyrocidin synthetase III	3.083	4.117	4.056	0.194

**SMU_1341c**	*grs*	gramicidin S synthase/mycosubtilin synthetase chain mycB	3.948	4.555	3.968	0.234
**SMU_1342**	*bacA1*	bacitracin synthetase	3.360	4.654	3.853	3.965
**SMU_1343c**	*pksC*	polyketide synthase	2.266	3.002	2.139	0.002
**SMU_1344c**	*fabD*	malonyl CoA-acyl carrier protein transacylase	3.157	4.291	3.107	0.001
**SMU_1345c**	*ituA*	peptide synthetase similar to mycA	3.807	6.412	5.640	2.313
**SMU_1346**	*bacT*	thioesterase II-like protein	1.845	2.207	1.481	0.002
**SMU_1349**	-	conserved hypothetical protein	1.867	3.798	2.296	0.000
**SMU_135**	*mleR*	transcriptional regulator, MleR-related / LysR-related	2.122	2.536	2.575	0.001
**SMU_1351**	-	transposase fragment	1.293	0.801	0.590	0.001
**SMU_1352**	-	transposase	1.581	1.056	1.312	0.000
**SMU_1353**	-	transposase	0.789	1.133	1.038	0.000
**SMU_1354c**	-	transposase	2.966	3.411	2.677	0.501
**SMU_1355c**	*tpn*	transposase	0.627	0.641	0.562	0.000
**SMU_1356c**	*tpn*	transposase	1.549	2.006	1.795	0.003
**SMU_1357**	-	transposase fragment, IS1562 similarity	2.447	3.401	2.688	0.001
**SMU_1358**	-	transposase fragment	1.945	1.226	0.605	0.000
**SMU_1359**	-	hypothetical protein	2.635	4.027	5.109	0.002
**SMU_1360c**	-	hypothetical protein	0.517	0.652	0.677	0.000
**SMU_1361c**	*yjjB*	transcriptional regulator	0.808	0.315	0.309	0.001
**SMU_1363c**	*tpn*	transposase fragment (IS605/IS200-like)	0.495	1.031	0.618	0.000
**SMU_1367c**	-	conserved hypothetical protein	1.208	1.235	1.234	0.015
**SMU_1368**	-	hypothetical protein	0.042	0.038	0.015	0.001
**SMU_1369**	-	hypothetical protein	0.224	0.192	0.243	0.000
**SMU_136c**	-	conserved hypothetical protein; probable transcriptional regulator	0.686	1.183	1.074	0.014
**SMU_137**	*mleS*	malolactic enzyme	1.276	1.447	1.327	0.002
**SMU_1372c**	*tpn*	transposase, IS861, IS3 family	0.515	0.796	0.745	0.000
**SMU_1373c**	-	hypothetical protein	0.181	0.258	0.160	0.000
**SMU_1374**	-	IS30 transposase related protein	1.207	1.349	1.156	0.002
**SMU_1375c**	*yqkA*	conserved hypothetical protein	1.054	1.036	0.878	0.000
**SMU_1377c**	-	conserved hypothetical protein	0.410	0.446	0.338	0.000
**SMU_1378**	-	hypothetical protein	1.054	1.141	1.278	0.003
**SMU_138**	*mleP*	malate permease/auxin efflux carrier	1.539	1.552	1.817	0.001
**SMU_1381**	*leuD*	3-isopropylmalate dehydratase, small subunit	0.766	0.620	0.682	0.002
**SMU_1382**	*leuC*	alpha-isopropylmalate isomerase large subunit	0.949	1.256	1.389	0.076

**SMU_1383**	*leuB*	3-isopropylmalate dehydrogenase	0.562	0.684	0.546	0.001
**SMU_1384**	*leuA*	2-isopropylmalate synthase	1.030	1.151	1.242	0.001
**SMU_1386**	*urk*	uridine kinase	1.757	1.261	1.915	0.001
**SMU_1387**	*mocA*	oxidoreductase	1.053	1.271	1.044	0.001
**SMU_1388**	*deaD*	ATP-dependent RNA helicase, DEAD/DEAH family	0.817	0.379	0.371	0.000
**SMU_1389**	*pckA*	phosphoenolpyruvate carboxykinase (ATP)	1.210	1.445	1.693	0.396
**SMU_139**	*oxdC*	oxalate decarboxylase	1.534	1.574	1.581	0.004
**SMU_1390**	-	conserved hypothetical protein	3.069	3.029	3.156	0.183
**SMU_1391 c**	-	hypothetical protein	2.841	4.987	5.796	0.554
**SMU_1392 c**	-	conserved hypothetical protein; possible acetyltransferase	0.547	0.756	0.809	0.006
**SMU_1393 c**	-	conserved hypothetical protein	1.261	1.210	1.286	0.000
**SMU_1394**	*lepA*	GTP-binding protein LepA	0.784	0.382	0.613	0.031
**SMU_1395 c**	-	hypothetical protein	2.338	2.177	2.977	0.003
**SMU_1396**	*gbpC*	glucan-binding protein C	1.321	1.513	1.448	0.015
**SMU_1397 c**	-	possible transcriptional repressor	2.089	2.224	2.824	0.001
**SMU_1398**	*irvR*	repressor protein - phage associated	1.965	2.652	1.892	317.744
**SMU_1399**	-	hypothetical protein	0.445	0.693	0.529	0.003
**SMU_14**	*hprT*	hypoxanthine-guanine phosphoribosyltransferase	0.359	0.028	0.002	0.001
**SMU_140**	*gshR*	glutathione reductase	1.361	1.654	1.471	0.001
**SMU_1400 c**	-	conserved hypothetical protein	0.643	0.582	0.689	0.000
**SMU_1402 c**	-	conserved hypothetical protein	0.708	1.225	1.158	0.002
**SMU_1403 c**	-	conserved hypothetical protein	1.304	1.906	2.077	0.039
**SMU_1404 c**	-	conserved hypothetical protein	2.117	2.130	2.323	0.044
**SMU_1405 c**	*cas9*	conserved hypothetical protein	2.093	2.490	2.560	1.318
**SMU_1406 c**	-	conserved hypothetical protein (possible oxidoreductase)	0.573	0.557	0.557	0.008
**SMU_1409 c**	*bglC*	transcriptional regulator	1.294	1.290	1.329	0.000
**SMU_141**	-	conserved hypothetical protein	2.306	2.540	2.444	0.265
**SMU_1410**	*frdC*	fumarate reductase	1.640	1.519	1.298	0.025
**SMU_1411**	-	conserved hypothetical protein	1.349	1.797	1.866	7.515
**SMU_1412 c**	*yhcA*	ABC transporter, ATP-binding and permease fusion protein	1.414	1.525	1.603	0.075
**SMU_1414 c**	-	conserved hypothetical protein	0.777	0.612	0.254	0.004
**SMU_1415 c**	*nagD*	N-acetylglucosamine catabolic protein	1.714	0.301	0.883	0.001
**SMU_1416 c**	*mutT*	mutator protein MutT	1.206	1.342	1.198	0.000
**SMU_1417 c**	-	acyl-ACP thioesterase	1.606	2.173	0.930	0.001
**SMU_1418**	*hemN*	coproporphyrinogen III oxidase	1.137	0.534	0.658	0.001

**SMU_1419**	*acrR2*	transcriptional regulator	1.289	0.868	1.163	0.001
**SMU_1420**	*qr1*	NADPH-quinone reductase	0.769	0.900	0.910	0.001
**SMU_1421**	*pdhC*	dihydrolipoamide acetyltransferase (acetoin dehydrogenase E2 component)	1.468	1.846	1.720	0.133
**SMU_1422**	*pdhB*	acetoin dehydrogenase E1 component	1.320	1.807	1.614	0.009
**SMU_1423**	*pdhA*	acetoin dehydrogenase E1 component	1.187	1.144	1.387	0.000
**SMU_1424**	*pdhD*	dihydrolipoamide dehydrogenase	1.112	1.250	1.208	0.001
**SMU_1425**	*clpB*	ATP-dependent Clp protease, ATP-binding subunit ClpB	0.863	1.120	1.216	0.009
**SMU_1426 c**	*glmM*	phosphoglucosamine mutase (phosphoacetylglucosamine mutase)	0.001	0.001	0.000	0.000
**SMU_1427 c**	-	conserved hypothetical protein	3.225	2.935	1.895	0.001
**SMU_1428 c**	-	conserved hypothetical protein	0.034	0.007	0.017	0.002
**SMU_1429**	*murC2*	UDP-N-acetylmuramyl tripeptide synthetase	0.002	0.000	0.001	0.011
**SMU_1430**	*cobQ*	cobyric acid synthase	0.687	0.284	0.287	0.001
**SMU_1431 c**	-	ABC transporter, ATP-binding protein	2.328	2.826	2.030	17.776
**SMU_1432 c**	*bgc*	glycosyl hydrolases family 8 protein; possible beta-glucanase	4.900	10.070	6.501	0.001
**SMU_1434 c**	*icaA*	glycosyl transferase	5.328	8.477	8.246	0.938
**SMU_1435 c**	-	hypothetical protein	3.631	6.809	6.002	0.003
**SMU_1436 c**	-	conserved hypothetical protein	6.659	8.276	10.636	0.128
**SMU_1437**	*epsC*	UDP-N-acetylglucosamine 2-epimerase	2.821	4.219	3.140	0.030
**SMU_1438 c**	*prtB*	conserved hypothetical protein; possible protease	1.771	1.937	1.648	0.016
**SMU_143c**	*def*	polypeptide deformylase (PDF)	0.055	0.043	0.026	0.001
**SMU_1442 c**	-	conserved hypothetical protein	1.626	2.143	1.518	0.001
**SMU_1443 c**	*estA*	tributyrin esterase	1.531	1.493	2.468	0.014
**SMU_1444 c**	*yqgA*	metallo-beta-lactamase superfamily proteinhetical protein	0.098	0.159	0.200	0.001
**SMU_1445 c**	*livG*	ABC transporter, ATP-binding protein	1.133	1.440	1.740	0.000
**SMU_1446 c**	-	ABC transporter, permease	1.272	1.532	1.636	0.002
**SMU_1447 c**	-	ABC transporter, substrate-binding protein	1.044	1.475	1.113	11.871
**SMU_1449**	*fbp*	fibronectin-binding, fibrinogen-binding protein-A	0.804	0.444	0.429	0.012
**SMU_144c**	-	possible transcriptional regulator	1.836	2.371	3.161	0.001
**SMU_145**	-	major facilitator superfamily transporter, efflux protein	2.660	1.957	2.629	0.001
**SMU_1450**	*aapA*	amino acid permease	0.744	0.633	1.313	0.000

**SMU_1451**	*aldB*	alpha-acetolactate decarboxylase	1.431	1.296	0.292	0.001
**SMU_1452**	*alsS*	alpha-acetolactate synthase	1.468	1.375	0.134	2.689
**SMU_1453c**	-	conserved hypothetical protein, tetratricopeptide repeat family	2.526	2.475	0.966	0.000
**SMU_1454c**	*yljI*	conserved hypothetical protein, possible permease	1.692	1.905	0.966	0.002
**SMU_1455**	*mutX*	mutator mutX protein	1.062	1.050	0.207	0.000
**SMU_1456c**	-	hypothetical protein	0.246	0.446	0.353	0.002
**SMU_1457**	*rmlB*	dTDP-glucose-4,6-dehydratase	0.000	0.000	0.000	0.021
**SMU_1459c**	-	conserved hypothetical protein	0.836	0.514	0.613	0.000
**SMU_1460**	*rmlC*	dTDP-4-dehydrorhamnose 3,5-epimerase	0.000	0.003	0.001	0.042
**SMU_1461**	*rmlA*	glucose-1-phosphate thymidyltransferase	0.000	0.001	0.001	0.000
**SMU_1462c**	-	oxidoreductase, DadA family protein/D-amino acid oxidase	0.915	0.616	0.670	0.001
**SMU_1463c**	-	conserved hypothetical protein, NIF3-related	0.612	0.464	0.550	0.004
**SMU_1464c**	*orf3*	conserved hypothetical protein	0.045	0.000	0.000	0.000
**SMU_1465c**	*dnaD*	DNA replication protein DnaD	0.000	0.000	0.000	0.008
**SMU_1466**	*metA*	homoserine O-succinyltransferase	0.540	0.456	0.857	0.000
**SMU_1467**	*apt*	adenine phosphoribosyltransferase	1.366	0.357	0.003	0.000
**SMU_1470c**	-	conserved hypothetical protein	1.728	1.636	1.545	0.000
**SMU_1471c**	-	conserved hypothetical protein	1.921	1.757	1.736	0.003
**SMU_1472**	*recJ*	single-strand DNA-specific exonuclease	1.117	0.639	0.845	0.001
**SMU_1473c**	-	oxidoreductase, short chain dehydrogenase/reductase family	1.424	1.264	1.127	0.001
**SMU_1474c**	*rnz*	ribonuclease Z	0.001	0.001	0.000	0.001
**SMU_1475c**	-	conserved hypothetical protein	0.389	0.062	0.040	0.001
**SMU_1476c**	*hflX*	GTP-binding protein	0.751	0.037	0.059	0.000
**SMU_1477**	*miaA*	tRNA isopentenylpyrophosphate transferase	0.328	0.072	0.060	0.001
**SMU_1479**	-	conserved hypothetical protein	0.633	0.579	0.938	0.061
**SMU_148**	*adhE*	alcohol-acetaldehyde dehydrogenase	1.541	1.369	0.212	0.003
**SMU_1480**	-	hypothetical protein	2.312	2.685	2.447	0.001
**SMU_1482c**	-	conserved hypothetical protein	0.820	1.238	1.292	0.000
**SMU_1483c**	-	conserved hypothetical protein (possible acetyltransferase)	1.630	2.252	2.495	0.002
**SMU_1484c**	-	conserved hypothetical protein	1.103	1.401	1.642	0.021
**SMU_1485c**	-	conserved hypothetical protein (possible endonuclease)	1.626	2.099	2.401	0.002

**SMU_1486c**	-	histidinol phosphatase	1.440	0.936	1.290	0.001
**SMU_1487**	-	conserved hypothetical protein	0.908	1.101	1.234	0.000
**SMU_1488c**	-	conserved hypothetical protein	2.294	2.611	1.999	0.000
**SMU_1489**	*lacX*	aldose 1-epimerase	0.880	1.035	1.343	0.000
**SMU_149**	-	transposase fragment (IS605/IS200-like)	3.785	4.209	4.685	0.002
**SMU_1490**	*lacG*	phospho-beta-D-galactosidase	1.254	1.478	1.549	1.540
**SMU_1491**	*lacE*	PTS system, lactose-specific component IIBC	1.029	1.269	2.147	0.000
**SMU_1492**	*lacF*	PTS system, cellobiose-specific IIA component	2.002	2.103	2.302	0.000
**SMU_1493**	*lacD*	tagatose-1,6-bisphosphate aldolase	0.701	0.958	1.528	0.000
**SMU_1494**	*lacC*	tagatose-6-phosphate kinase	0.552	0.394	0.436	0.010
**SMU_1495**	*lacB*	galactose-6-phosphate isomerase	2.718	2.067	3.192	0.000
**SMU_1496**	*lacA*	galactose-6-phosphate isomerase	0.867	0.215	1.655	0.001
**SMU_1498**	*lacR*	lactose phosphotransferase system repressor	0.632	0.113	0.539	0.006
**SMU_1499**	*rexA*	ATP-dependent exonuclease subunit A	0.711	0.017	0.023	0.001
**SMU_15**	*ftsH*	cell division protein FtsH	0.003	0.001	0.001	0.001
**SMU_150**	*nlmA*	non-lantibiotic mutacin IV A	1.874	1.620	1.229	0.001
**SMU_1500**	*rexB*	ATP-dependent exonuclease subunit B	0.569	0.004	0.001	0.001
**SMU_1502c**	-	conserved hypothetical protein	4.385	6.335	6.528	0.000
**SMU_1504c**	-	hypothetical protein	1.465	1.476	2.075	0.002
**SMU_1506c**	-	conserved hypothetical protein	1.792	2.181	2.495	0.371
**SMU_1507c**	-	hypothetical protein	9.314	11.744	15.025	3.289
**SMU_1508c**	-	coenzyme PQQ synthesis protein /possible oxidoreductase	2.729	3.605	4.056	0.648
**SMU_1509**	*rgg*	transcriptional regulator	1.915	2.231	2.304	0.204
**SMU_151**	*nlmB*	non-lantibiotic mutacin IV B	4.196	3.585	4.183	0.001
**SMU_1510**	*syfB*	phenylalanyl-tRNA synthetase, beta subunit	0.001	0.001	0.000	0.001
**SMU_1511c**	-	acetyltransferase; possible transcriptional repressor	0.253	0.382	0.407	0.003
**SMU_1512**	*pheS*	phenylalanyl-tRNA synthetase, alpha subunit	0.000	0.000	0.001	0.351
**SMU_1513**	*smc*	chromosome segregation SMC protein	0.961	0.178	0.067	0.000
**SMU_1514**	*rnc*	ribonuclease III	0.067	0.001	0.006	0.002
**SMU_1515**	*covX*	gtfB/C regulator, metallo-beta-lactamase superfamily	1.052	0.447	0.536	0.000
**SMU_1516**	*covS*	two-component sensor histidine kinase	0.964	0.024	0.245	0.001
**SMU_1517**	*covR*	two-component response regulator	0.256	0.188	0.130	0.001

**SMU_1519**	*glnQ*	glutamine ABC transport, ATP-binding protein	1.364	0.241	0.849	0.000
**SMU_152**	-	hypothetical protein	3.378	3.124	3.932	0.043
**SMU_1520**	*glnH*	amino acid ABC transporter, substrate-binding protein	1.554	0.225	0.914	0.001
**SMU_1521**	*glnM*	amino acid (glutamine) ABC transporter permease	2.358	0.400	1.071	0.004
**SMU_1522**	*glnP*	amino acid (glutamine) ABC transporter permease	1.217	0.190	0.490	0.003
**SMU_1523**	*endA*	competence associated membrane nuclease (DNA-entry nuclease)	2.093	1.029	1.207	0.001
**SMU_1524c**	*epuA*	conserved hypothetical protein	0.682	0.003	0.030	0.001
**SMU_1525**	*murA*	UDP-N-acetylglucosamine 1-carboxyvinyltransferase	1.232	0.766	0.694	0.000
**SMU_1526c**	-	conserved hypothetical protein	1.603	0.596	0.996	0.005
**SMU_1527**	*atpA*	proton-translocating ATPase epsilon subunit	0.141	0.007	0.009	0.000
**SMU_1528**	*atpB*	ATPase beta subunit	0.000	0.000	0.000	0.000
**SMU_1529**	*atpC*	ATPase, gamma subunit	0.007	0.002	0.001	0.000
**SMU_153**	-	hypothetical protein	2.010	3.050	4.120	0.003
**SMU_1530**	*atpD*	ATP synthase alpha subunit	0.002	0.000	0.001	0.001
**SMU_1531**	*atpE*	ATPase delta subunit	0.019	0.024	0.006	0.001
**SMU_1532**	*atpF*	ATPase, b subunit	0.014	0.000	0.001	0.000
**SMU_1533**	*atpG*	ATP synthase subunit A	0.211	0.096	0.077	0.001
**SMU_1534**	*atpH*	ATPase, c subunit	0.005	0.000	0.000	0.001
**SMU_1535**	*phsG*	glycogen phosphorylase	1.900	2.145	2.179	0.078
**SMU_1536**	*glgA*	glycogen synthase	1.467	1.358	1.434	0.001
**SMU_1537**	*glgD*	glycogen biosynthesis protein	2.304	2.863	3.014	0.003
**SMU_1538**	*glgC*	glucose-1-phosphate adenylyltransferase	2.149	2.659	2.884	0.009
**SMU_1539**	*glgB*	1,4-alpha-glucan branching enzyme	1.866	2.123	2.346	0.001
**SMU_154**	*rpsO*	30S ribosomal protein S15	0.027	0.000	0.002	0.000
**SMU_1541**	*pulA*	pullulanase	3.025	2.595	1.988	0.191
**SMU_1542c**	-	conserved hypothetical protein, diacylglycerol kinase catalytic domain	0.136	0.004	0.003	0.003
**SMU_1543**	*ligA*	DNA ligase	0.000	0.001	0.000	0.001
**SMU_1545c**	-	conserved hypothetical protein	4.204	4.187	4.207	0.721
**SMU_1546**	-	conserved hypothetical protein	1.437	0.021	0.002	0.013
**SMU_1547c**	*rr11*	response regulator	2.329	2.163	1.706	0.000
**SMU_1548c**	*hk11*	sensor histidine kinase	1.362	1.460	1.018	0.001
**SMU_155**	*pnpA*	polyribonucleotide nucleotidyltransferase alpha subunit (PNPase)	0.519	0.090	0.164	0.021
**SMU_1550c**	-	ABC transporter, membrane spanning permease	3.600	3.983	3.300	2.852
**SMU_1551c**	*yvfR*	ABC transporter, ATP-binding protein	3.498	3.912	2.493	1.047

**SMU_1552c**	-	hypothetical protein	1.717	2.121	1.414	0.000
**SMU_1553c**	-	hypothetical protein	2.385	2.404	2.078	0.002
**SMU_1554c**	-	conserved hypothetical protein	3.501	4.022	2.419	0.001
**SMU_1555c**	-	ribonuclease BN-like family protein	2.261	2.353	1.402	0.003
**SMU_1556**	*map*	methionine aminopeptidase	0.088	0.026	0.014	0.000
**SMU_1557c**	-	conserved hypothetical protein, DRTGG and CBS domains	0.201	0.031	0.101	0.000
**SMU_1558c**	*yhdC*	acetyltransferase, GNAT family	0.641	0.400	0.479	0.000
**SMU_156**	-	conserved hypothetical protein	0.738	0.677	0.365	0.001
**SMU_1560**	-	conserved hypothetical protein	0.812	0.660	1.534	0.018
**SMU_1561**	*trkB*	potassium uptake protein B	1.485	1.261	1.764	0.256
**SMU_1562**	*trk*	potassium uptake protein A	1.182	0.968	1.029	0.000
**SMU_1563**	*pacL*	cation-transporting P-ATPase	1.170	1.021	1.038	0.008
**SMU_1564**	*glgP*	glycogen phosphorylase	1.554	1.678	1.180	0.002
**SMU_1565**	*malQ*	4-alpha-glucanotransferase	0.283	0.092	0.025	0.000
**SMU_1566**	*malR*	maltose operon transcriptional repressor	1.424	0.047	0.025	0.001
**SMU_1568**	*malX*	maltose / maltodextrin-binding protein	1.641	1.914	1.604	0.124
**SMU_1569**	*malF*	maltodextrin ABC transport system permease	1.371	1.931	1.607	0.001
**SMU_157**	*cysE*	serine O-acetyltransferase	0.057	0.010	0.011	0.000
**SMU_1570**	*malG*	maltose / maltodextrin ABC transport system (permease)	2.200	3.270	2.872	0.004
**SMU_1571**	*msmK*	ABC-type transport system ATP-binding protein (maltose)	1.204	1.542	1.654	0.001
**SMU_1572**	*murZ*	UDP-N-acetylglucosamine 1-carboxyvinyltransferase	1.808	3.175	1.132	0.001
**SMU_1573**	*metK*	S-adenosylmethionine synthetase	0.000	0.000	0.000	0.000
**SMU_1574c**	-	conserved hypothetical protein	2.601	3.837	2.819	4.556
**SMU_1575c**	-	hypothetical protein	2.039	3.326	3.356	1.010
**SMU_1576c**	-	hypothetical protein	4.849	7.855	6.329	0.009
**SMU_1577c**	-	conserved hypothetical protein	4.545	5.156	4.572	0.074
**SMU_1578**	*birA*	biotin--[acetyl-CoA-carboxylase] ligase	0.132	0.029	0.051	0.003
**SMU_1579**	-	hypothetical protein	2.444	2.613	2.814	0.000
**SMU_158**	*cysS*	cysteinyl-tRNA synthetase	0.007	0.002	0.001	0.001
**SMU_1581**	*dnaX*	DNA polymerase III subunits gamma and tau	0.000	0.000	0.000	0.002
**SMU_1582c**	-	conserved hypothetical protein	0.995	1.165	1.380	0.000
**SMU_1584c**	*mycA*	67 kDa myosin-crossreactive streptococcal antigen	1.175	0.916	1.159	0.040
**SMU_1585c**	-	transcriptional regulator	1.129	1.278	1.251	0.021
**SMU_1586**	*thrS*	threonyl-tRNA synthetase	0.026	0.024	0.022	0.013

**SMU_1587c**	-	hypothetical protein	0.684	0.461	0.556	0.001
**SMU_1588c**	*rfaG*	glucosyl transferase, family 1	0.017	0.003	0.003	0.008
**SMU_1589c**	*cpoA*	glycosyl transferase, family 1	0.087	0.014	0.012	0.001
**SMU_159**	-	conserved hypothetical protein	1.636	0.618	0.840	0.001
**SMU_1590**	*amyA*	intracellular a-amylase	1.379	1.487	1.925	1.643
**SMU_1591**	*ccpA*	catabolite control protein A	0.663	0.024	0.588	0.001
**SMU_1592**	*pepQ*	proline dipeptidase	0.232	0.124	0.144	0.000
**SMU_1593c**	-	conserved hypothetical protein (possible CDP-diglyceride synthetase)	0.830	0.689	0.835	0.001
**SMU_1595**	*cah*	carbonic anhydrase (carbonate dehydratase)	0.910	1.446	1.272	0.001
**SMU_1596**	*celB*	PTS system, cellobiose-specific IIC component	2.262	2.927	3.704	0.007
**SMU_1597c**	-	conserved hypothetical protein	2.645	3.106	3.694	0.001
**SMU_1598**	*celC*	PTS system IIA component, required for cellobiose uptake and metabolism	1.394	1.238	0.718	0.001
**SMU_1599**	*celR*	transcriptional regulator, required for cellobiose uptake and metabolism	2.040	2.392	2.341	0.098
**SMU_16**	-	amino acid permease	1.231	1.484	1.593	0.002
**SMU_160**	-	conserved hypothetical protein	1.441	1.009	0.890	0.164
**SMU_1600**	*celB*	PTS system IIB component, required for cellobiose uptake and metabolism	1.658	1.203	0.993	0.000
**SMU_1601**	*bgl*	6-phospho-beta-glucosidase, required for cellobiose uptake and metabolism	2.006	2.346	2.705	0.000
**SMU_1602**	*frp*	NAD(P)H-flavin oxidoreductase	0.997	1.196	1.647	0.001
**SMU_1603**	*lguL*	lactoylglutathione lyase	1.883	1.749	1.337	0.000
**SMU_1604c**	-	conserved hypothetical protein	1.969	0.825	0.591	0.002
**SMU_1605**	-	MDR-type permease;transmembrane efflux protein	2.645	2.293	2.221	0.190
**SMU_1606**	*smpB*	SsrA-binding protein (small protein B)	0.579	0.005	0.051	0.000
**SMU_1607**	*vacB*	exoribonuclease R	1.337	0.503	0.687	0.000
**SMU_1609c**	*secG*	preprotein translocase, SecG subunit	0.004	0.006	0.005	0.002
**SMU_161**	-	transcriptional regulator (MerR family?)	1.379	1.498	0.934	0.001
**SMU_1610**	*rpmG*	50S ribosomal protein L33	1.605	1.464	2.213	0.000
**SMU_1611c**	*pmrA*	multi-drug resistance efflux pump, major facilitator superfamily	1.809	1.383	1.767	0.002
**SMU_1612c**	-	conserved hypothetical protein	2.595	1.015	1.468	0.000
**SMU_1613c**	-	dephospho-CoA kinase	0.001	0.000	0.000	0.000

**SMU_1614**	*fpg*	formamidopyrimidine-DNA glycosylase	0.498	0.384	0.424	0.216
**SMU_1615 c**	-	conserved hypothetical protein	1.356	2.054	1.775	0.001
**SMU_1616 c**	-	phosphohydrolase MutT/nudix family protein	0.265	0.295	0.363	0.002
**SMU_1617**	*era*	GTP-binding protein era homolog.	0.022	0.023	0.052	0.004
**SMU_1618**	*dgk*	diacylglycerol kinase	0.005	0.004	0.001	0.001
**SMU_1619 c**	-	conserved hypothetical protein	0.286	0.383	0.379	0.008
**SMU_1620**	*phoH*	phosphate starvation-induced protein	1.208	1.524	1.221	0.221
**SMU_1621 c**	-	conserved hypothetical protein	1.306	1.516	0.747	0.001
**SMU_1622**	*msrA*	peptide methionine sulfoxide reductase A	1.737	2.717	2.314	0.000
**SMU_1623 c**	-	conserved hypothetical protein	1.211	0.721	0.692	0.001
**SMU_1624**	*rrf1*	ribosome recycling factor	0.037	0.000	0.002	0.000
**SMU_1625**	*pyrH*	uridylate kinase (UMP-kinase)	0.000	0.001	0.000	0.000
**SMU_1626**	*rplA*	50S ribosomal protein L1	0.000	0.001	0.000	0.000
**SMU_1627**	*rplK*	50S ribosomal protein L11	0.000	0.000	0.000	0.000
**SMU_1628**	-	conserved hypothetical protein	3.183	4.736	4.914	0.001
**SMU_1629 c**	-	cell division protein	0.058	0.007	0.001	0.000
**SMU_162c**	-	conserved hypothetical protein	1.736	2.743	1.850	0.036
**SMU_1631**	*ppiA*	peptidyl-prolyl cis-trans isomerase; cyclophilin	2.482	1.884	2.955	0.002
**SMU_1632**	*mtnN*	MTA/SAH nucleosidase	0.024	0.000	0.001	0.004
**SMU_1633 c**	-	conserved hypothetical protein	0.488	0.870	0.274	0.000
**SMU_1634 c**	-	conserved hypothetical protein, MutT/nudix family	0.167	0.253	0.388	0.004
**SMU_1635**	*glmU*	UDP-N-acetylglucosamine pyrophosphorylase	0.017	0.029	0.012	0.002
**SMU_1636 c**	-	conserved hypothetical protein	0.589	1.138	1.044	0.000
**SMU_1637 c**	-	hypothetical protein	0.444	0.415	0.466	0.001
**SMU_1638 c**	-	hypothetical protein	0.247	0.466	0.452	0.000
**SMU_1639**	*metS*	methionyl-tRNA synthetase	0.013	0.006	0.006	0.000
**SMU_163c**	-	hypothetical protein	1.213	1.344	1.043	0.001
**SMU_164**	*spoU*	tRNA/rRNA methyltransferase	1.980	1.508	0.954	0.002
**SMU_1641 c**	-	conserved hypothetical protein	1.538	2.042	2.014	0.001
**SMU_1642 c**	-	conserved hypothetical protein	0.974	1.115	0.940	0.000
**SMU_1643 c**	-	conserved hypothetical protein	2.531	3.294	3.171	3.271
**SMU_1644 c**	-	conserved hypothetical protein	3.862	5.662	4.181	1.821
**SMU_1645**	*tehB*	tellurite resistance protein	3.043	3.117	3.261	0.600
**SMU_1646 c**	-	conserved hypothetical protein (possible hemolysis inducing protein)	2.600	3.215	2.880	69.900

**SMU_1647c**	-	transcriptional regulator	1.208	1.166	0.911	0.000
**SMU_1648c**	-	hypothetical protein	1.787	1.970	2.173	0.002
**SMU_1649**	*exoA*	exodeoxyribonuclease III/ Smx nuclease	0.642	0.196	0.143	0.001
**SMU_165**	-	fatty acid-binding protein, DegV family	0.224	0.008	0.358	0.001
**SMU_1650**	*end3*	endonuclease III (DNA repair)	0.840	0.818	0.400	0.000
**SMU_1651**	*arsC*	arsenate reductase	1.671	0.972	0.565	0.091
**SMU_1652**	*ogt*	methylated-DNA--protein-cysteine S-methyltransferase	0.687	0.718	0.428	0.000
**SMU_1653**	*serA*	D-3-phosphoglycerate dehydrogenase	1.185	0.145	0.002	0.001
**SMU_1654c**	-	acetyltransferase	1.004	0.724	0.560	0.001
**SMU_1655c**	-	hypothetical protein	0.115	0.120	0.043	0.003
**SMU_1656**	*serC*	phosphoserine aminotransferase	1.294	0.167	0.050	0.000
**SMU_1657c**	*glnB*	nitrogen regulatory protein PII	0.976	1.022	2.273	0.000
**SMU_1658**	*nrgA*	ammonium transporter, NrgA protein	2.275	2.424	2.362	0.001
**SMU_1659c**	-	conserved hypothetical protein, tetrapyrrole methylase family	1.581	0.735	0.863	0.001
**SMU_166**	-	hypothetical protein	2.075	1.968	2.486	1.995
**SMU_1660c**	-	conserved hypothetical protein	1.498	0.391	0.645	0.000
**SMU_1661c**	*lspA*	signal peptidase II	1.005	0.610	0.227	0.000
**SMU_1662**	*holB*	DNA polymerase III delta prime subunit	0.085	0.100	0.090	0.000
**SMU_1663**	*kthY*	thymidylate kinase	0.005	0.003	0.009	0.001
**SMU_1664c**	*acoB*	acetoin utilization protein, acetoin dehydrogenase	1.220	1.161	0.900	0.001
**SMU_1665**	*livF*	branched chain amino acid ABC transporter, ATP-binding protein	0.388	0.540	0.059	0.002
**SMU_1666**	*livG*	branched chain amino acid ABC transporter, ATP-binding protein	0.955	0.963	0.082	0.000
**SMU_1667**	*livM*	branched chain amino acid ABC transporter, permease	1.294	0.933	0.125	0.001
**SMU_1668**	*livH*	branched chain amino acid ABC transporter, permease	2.282	1.788	0.200	0.001
**SMU_1669**	*livK*	branched-chain amino acid ABC transporter, substrate-binding protein	2.363	2.072	0.192	0.941
**SMU_167**	-	hypothetical protein	3.343	2.872	2.945	0.002
**SMU_1670c**	-	conserved hypothetical protein	5.108	4.038	7.062	0.002
**SMU_1671c**	-	conserved hypothetical protein	1.350	1.218	0.869	0.001
**SMU_1672**	*clpP*	ATP-dependent Clp protease, proteolytic subunit	0.402	0.009	0.010	0.000
**SMU_1673**	*upp*	uracil phosphoribosyltransferase	2.221	1.660	1.485	0.000
**SMU_1674**	*patB*	aminotransferase class II	1.893	5.381	0.883	0.001
**SMU_1675**	*metB*	cystathionine gamma-synthase	1.974	1.365	0.663	0.000

**SMU_1676c**	-	polysaccharide transporter	0.126	0.096	0.049	0.002
**SMU_1677**	*murE*	UDP-N-acetylmuramoylananine-D-glutamate-2,6-diaminopimelate ligase; UDP-MurNac-tripeptide synthetase	0.070	0.005	0.002	0.001
**SMU_1678**	-	conserved hypothetical protein	3.077	1.513	1.530	0.000
**SMU_1679c**	-	conserved hypothetical protein	2.353	0.764	0.889	0.000
**SMU_168**	-	transcriptional regulator	0.668	0.410	0.113	0.000
**SMU_1680c**	-	hypothetical protein	1.681	0.859	0.800	0.000
**SMU_1681c**	-	conserved hypothetical protein	0.622	0.454	0.538	1.004
**SMU_1682c**	-	conserved hypothetical protein (possible intracellular protease)	2.007	0.363	0.708	0.008
**SMU_1683c**	-	conserved hypothetical protein	1.460	1.056	1.084	0.005
**SMU_1685c**	*orf11*	conserved hypothetical protein	2.053	0.718	0.664	0.001
**SMU_1687**	*ppaC*	manganese-dependent inorganic pyrophosphatase (type II PPase)	0.001	0.005	0.007	0.000
**SMU_1688**	*dltD*	integral membrane protein	3.290	4.951	0.059	0.000
**SMU_1689**	*dltC*	D-alanyl carrier protein	4.522	19.069	0.002	0.001
**SMU_169**	-	50S ribosomal protein L13	0.001	0.000	0.000	0.000
**SMU_1690**	*dltB*	integral membrane protein, D-alanyl transfer	4.200	9.176	0.021	0.099
**SMU_1691**	*dltA*	D-alanine-D-alanyl carrier protein ligase	4.805	9.732	0.004	0.213
**SMU_1692**	*pflA*	pyruvate-formate lyase activating enzyme	2.012	1.748	1.924	0.000
**SMU_1693**	*hlyX*	uncharacterized hemolysin	1.346	1.222	2.817	0.072
**SMU_1694c**	-	permease (possible ABC transporter permease)	2.557	2.885	2.983	0.003
**SMU_1695**	-	ABC transporter, ATP-binding protein; possible molybdenum transport system	1.779	1.436	1.113	0.000
**SMU_1697c**	-	possible rRNA methylase, SAM-dependent methyltransferase superfamily	1.731	2.050	1.888	0.001
**SMU_1699c**	-	conserved hypothetical protein, radical SAM superfamily	1.452	1.089	1.000	0.000
**SMU_170**	*rpsI*	30S ribosomal protein S9	0.014	0.001	0.000	0.004
**SMU_1700c**	-	LrgB-like protein; possible murein hydrolase regulator	1.885	1.668	1.594	0.002
**SMU_1701c**	-	conserved hypothetical protein	1.240	1.143	1.174	0.002
**SMU_1702c**	-	uncharacterized phosphatase; possible PAP2 family protein	2.736	3.626	2.223	0.002
**SMU_1703c**	-	conserved hypothetical protein	0.092	0.200	0.222	0.003
**SMU_1704**	-	conserved hypothetical protein	3.632	4.446	3.644	0.025
**SMU_1705**	-	conserved hypothetical protein	0.757	0.769	0.744	0.015
**SMU_1706**	-	conserved hypothetical protein	2.068	2.855	2.914	0.188
**SMU_1707c**	*trmH*	spoU-related rRNA/tRNA methylase	2.492	3.620	3.241	0.001

**SMU_1708**	*trkA*	potassium uptake system protein	0.448	0.026	0.008	0.010
**SMU_1709**	*trkH*	potassium uptake protein TrkH	0.622	0.020	0.002	0.002
**SMU_1710 c**	-	conserved hypothetical protein	0.864	0.889	0.689	0.000
**SMU_1711**	*rluB*	ribosomal large subunit pseudouridine synthase B	1.007	0.242	0.240	0.000
**SMU_1712 c**	*scpB*	segregation and condensation protein B	1.100	0.161	0.057	0.000
**SMU_1713 c**	*scpA*	segregation and condensation protein A	1.642	0.290	0.081	0.001
**SMU_1714 c**	*xerD*	tyrosine recombinase	0.812	0.338	0.376	0.001
**SMU_1715 c**	-	CBS domain protein	1.047	1.203	1.105	0.000
**SMU_1716 c**	-	possible phosphoesterase	0.678	0.467	0.403	0.001
**SMU_1717 c**	*rdgB*	HAM1-like protein	0.389	0.297	0.328	0.000
**SMU_1718**	*murI*	glutamate racemase	0.015	0.069	0.071	0.000
**SMU_1719 c**	-	conserved hypothetical protein	1.701	1.173	1.313	0.023
**SMU_172**	-	conserved hypothetical protein (possible cell growth regulatory protein)	1.603	1.258	0.781	0.000
**SMU_1721 c**	*lysA*	diaminopimelate decarboxylase	1.313	0.657	0.456	0.000
**SMU_1722 c**	-	conserved hypothetical protein, membrane protein	2.033	1.685	2.309	0.147
**SMU_1723 c**	-	conserved hypothetical protein	3.430	2.588	2.788	0.000
**SMU_1724 c**	*cspR*	rRNA methyltransferase	1.545	1.265	1.275	0.001
**SMU_1725**	-	acylphosphatase	2.055	1.690	1.827	0.011
**SMU_1727**	*yidC2*	OxaA-like protein; inner membrane protein	0.233	0.257	0.060	0.000
**SMU_1728**	*greA*	transcription elongation factor GreA	0.114	0.003	0.002	0.001
**SMU_1729 c**	*pabC*	aminodeoxychorismate lyase-like protein (fragment)	1.055	0.293	0.079	0.001
**SMU_173**	-	conserved hypothetical protein (possible ppGpp-regulated growth inhibitor)	2.131	2.398	2.773	0.005
**SMU_1730 c**	-	arylalkylamine n-acetyltransferase	0.530	0.753	1.398	0.000
**SMU_1731**	*murC*	UDP-N-acetylmuramate--L-alanine ligase	0.022	0.094	0.072	0.000
**SMU_1732 c**	-	conserved hypothetical protein	1.352	0.898	1.531	0.001
**SMU_1733 c**	*snf*	SNF helicase	1.655	1.348	0.996	0.181
**SMU_1734**	*accA*	acetyl-CoA carboxylase alpha subunit	0.001	0.001	0.000	0.001
**SMU_1735**	*accD*	acetyl-CoA carboxylase beta subunit	0.001	0.001	0.001	0.000
**SMU_1736**	*accC*	acetyl-CoA carboxylase biotin carboxylase subunit	0.002	0.001	0.001	0.001

**SMU_1737**	*fabZ*	(3R)-hydroxymyristoyl-(acyl carrier protein) dehydratase	0.007	0.001	0.001	0.000
**SMU_1738**	*bccP*	biotin carboxyl carrier protein of acetyl-CoA carboxylase	0.000	0.000	0.001	0.000
**SMU_1739**	*fabF*	3-oxoacyl-(acyl-carrier-protein) synthase	0.029	0.000	0.000	0.002
**SMU_1740**	*fabG*	3-oxoacyl-acyl-carrier-protein reductase / 3-ketoacyl-acyl carrier protein reductase	0.007	0.001	0.001	0.002
**SMU_1741**	*fabD*	[acyl-carrier-protein] S-malonyltransferase	0.262	0.032	0.043	0.001
**SMU_1742c**	-	trans-2-enoyl-ACP reductase II	0.002	0.000	0.000	0.000
**SMU_1743**	*acp*	acyl carrier protein	0.007	0.015	0.051	0.000
**SMU_1744**	*fabH*	3-oxoacyl-[acyl-carrier-protein] synthase III	0.082	0.001	0.001	0.001
**SMU_1745c**	-	transcriptional regulator, MarR family	0.547	0.162	0.058	0.000
**SMU_1746c**	*fabM*	enoyl-CoA hydratase/trans-2, cis-3-decenoyl-ACP isomerase	0.662	0.000	0.000	0.001
**SMU_1747c**	-	possible phosphatase	1.367	1.401	1.741	0.000
**SMU_1748**	*lysC*	aspartate kinase / homoserine dehydrogenase	0.002	0.001	0.000	0.001
**SMU_174c**	-	conserved hypothetical protein	4.976	6.061	6.021	0.541
**SMU_1750c**	-	hypothetical protein	0.670	0.829	0.980	0.000
**SMU_1752c**	-	hypothetical protein	1.004	0.898	0.882	0.027
**SMU_1753c**	-	conserved hypothetical protein	0.312	0.311	0.360	0.003
**SMU_1754c**	-	conserved hypothetical protein	3.976	4.088	3.421	0.000
**SMU_1755c**	-	conserved hypothetical protein	0.465	0.619	0.585	0.001
**SMU_1757c**	-	conserved hypothetical protein	2.284	2.270	2.328	0.002
**SMU_1758c**	-	conserved hypothetical protein	2.127	2.001	1.970	0.000
**SMU_176**	-	hypothetical protein	3.001	2.651	3.015	0.008
**SMU_1760c**	-	conserved hypothetical protein	1.951	1.796	1.716	0.028
**SMU_1761c**	-	conserved hypothetical protein	2.215	1.777	1.560	0.451
**SMU_1762c**	-	conserved hypothetical protein	4.351	2.856	3.074	0.001
**SMU_1763c**	-	conserved hypothetical protein	1.921	1.698	1.246	0.000
**SMU_1764c**	-	conserved hypothetical protein	2.121	1.600	1.410	0.003
**SMU_1765c**	-	conserved hypothetical protein	1.589	0.779	0.743	0.004
**SMU_1766c**	-	hypothetical protein	0.220	0.168	0.104	1.035
**SMU_1767c**	-	hypothetical protein	0.779	1.051	1.054	0.002
**SMU_1768c**	-	hypothetical protein	0.615	1.049	0.869	0.000

**SMU_177**	-	PTS cellobiose-specific IIC component)	2.649	3.531	4.303	3.263
**SMU_1770**	*syv*	valyl-tRNA synthetase	0.065	0.051	0.042	0.000
**SMU_1771c**	-	hypothetical protein	1.980	1.716	3.076	0.003
**SMU_1772c**	-	hypothetical protein	3.955	4.610	4.955	0.000
**SMU_1773c**	-	conserved hypothetical protein	2.191	1.710	2.429	0.001
**SMU_1774c**	-	conserved hypothetical protein	1.900	1.687	3.987	1.956
**SMU_1775c**	-	hypothetical protein	3.333	3.394	4.631	0.001
**SMU_1776c**	-	conserved hypothetical protein	2.319	3.046	2.593	0.001
**SMU_1777**	*nrdI*	ribonucleotide reductase protein, Nrd	0.471	0.440	0.561	0.000
**SMU_1779c**	*trmA*	RNA methyltransferase, TrmA family	1.086	0.667	0.981	0.004
**SMU_178**	-	conserved hypothetical protein	1.927	3.987	4.327	0.001
**SMU_1780**	*recX*	RecA regulator RecX	1.221	1.365	1.126	0.000
**SMU_1781**	-	conserved hypothetical protein	5.108	0.320	0.265	0.001
**SMU_1782**	-	conserved hypothetical protein	0.987	1.068	1.374	0.000
**SMU_1783**	*proS*	prolyl-tRNA synthetase	0.001	0.001	0.000	0.001
**SMU_1784c**	*eep*	conserved hypothetical protein, peptidase family M50	0.365	0.281	0.156	0.003
**SMU_1785**	*cdsA*	phosphatidate cytidylyltransferase (CDP-diglyceride synthase)	0.002	0.002	0.002	0.002
**SMU_1786**	*uppS*	undecaprenyl pyrophosphate synthetase	0.019	0.007	0.011	0.001
**SMU_1787c**	-	preprotein translocase, YajC subunit	0.305	0.362	0.292	0.001
**SMU_1788c**	*bta*	bacterocin transport accessory protein	1.143	0.834	0.506	0.000
**SMU_1789c**	-	conserved hypothetical protein	0.761	0.356	0.660	0.000
**SMU_179**	-	conserved hypothetical protein (possible oxidoreductase)	1.111	1.917	2.553	0.059
**SMU_1790c**	-	transcriptional regulator, probable MerR family	1.717	1.119	1.557	0.002
**SMU_1791c**	-	conserved hypothetical protein	0.532	0.412	0.296	0.035
**SMU_1792c**	-	hypothetical protein	5.789	3.131	1.921	0.000
**SMU_1794c**	-	hypothetical protein	3.326	2.102	1.713	0.001
**SMU_1795c**	-	conserved hypothetical protein; possible methyltransferase	2.258	1.110	0.799	0.015
**SMU_1797c**	-	conserved hypothetical protein	1.337	2.096	1.441	0.000
**SMU_1798c**	-	conserved hypothetical protein	1.221	0.026	0.028	0.000
**SMU_1799**	*nadD*	nicotinate mononucleotide adenylyltransferase (nicotinate-nucleotide adenylyltransferase)	0.001	0.002	0.001	0.001
**SMU_18**	-	hypothetical protein	0.000	0.000	0.000	0.002

**SMU_180**	-	oxidoreductase, possible fumarate reductase	1.459	1.920	2.070	0.003
**SMU_1800c**	-	conserved hypothetical protein	0.517	0.264	0.139	0.001
**SMU_1801c**	-	conserved hypothetical protein, GTP-binding	0.001	0.001	0.001	0.000
**SMU_1802c**	-	conserved hypothetical protein	0.004	0.001	0.001	0.009
**SMU_1803c**	-	conserved hypothetical protein	2.477	3.875	3.658	0.001
**SMU_1804c**	-	hypothetical protein	3.600	3.245	3.757	0.000
**SMU_1805**	-	transcriptional regulator	0.536	0.703	0.887	0.000
**SMU_1806**	-	macrolide-related glycosyltransferase	2.333	2.309	2.430	0.004
**SMU_1807c**	-	permease	2.734	3.061	2.957	0.793
**SMU_1809**	*scnG*	cdd2-related, bacteriocin related ABC element	3.417	4.302	4.277	8.773
**SMU_181**	*mvaK*	mevalonate kinase	0.186	0.284	0.000	0.005
**SMU_1810**	*scnE*	ABC transporter, membrane spanning permease	5.798	6.679	6.561	0.001
**SMU_1811**	*scnF*	cdd4-like bacteriocin component, ScnF homolog	1.983	1.637	1.447	0.001
**SMU_1812**	*tpn*	transposase, IS1193n-related	2.091	1.855	2.063	0.000
**SMU_1813**	-	hypothetical protein, probable transposase fragment	2.122	1.566	1.334	0.000
**SMU_1814**	*scnK*	histidine kinase, ScnK homolog	2.914	2.615	2.500	0.001
**SMU_1815**	*scnR*	response regulator, ScnR homolog	2.085	1.887	2.021	0.000
**SMU_1816c**	-	transposon-related, maturase-related protein fragment	2.094	1.097	1.104	0.000
**SMU_1817c**	-	transposon-related, maturase-related protein fragment	2.245	1.258	0.947	0.000
**SMU_1818c**	-	hypothetical protein	0.997	0.313	0.412	0.000
**SMU_1819**	*gatB*	glutamyl-tRNA (Gln) amidotransferase subunit B	0.008	0.001	0.000	0.001
**SMU_182**	*sloA*	ABC transporter, ATP-binding protein, iron and/or manganese	1.350	1.595	0.705	0.104
**SMU_1820c**	*gatA*	glutamyl-tRNA(Gln) amidotransferase subunit A	0.003	0.000	0.002	0.000
**SMU_1821c**	*gatC*	glutamyl-tRNA (Gln) amidotransferase subunit C	0.000	0.000	0.001	0.001
**SMU_1822**	*gatA*	aspartyl-tRNA synthetase	0.126	0.024	0.000	0.001
**SMU_1823**	*pncA*	pyrazinamidase/nicotinamidase	1.728	1.291	1.592	0.000
**SMU_1824c**	-	GTP-sensing transcriptional repressor	2.100	0.134	0.335	0.000
**SMU_1826**	*yfbQ*	aminotransferase	1.126	1.346	0.266	0.004
**SMU_1827**	*bioB*	biotin biosynthesis protein	2.118	0.110	0.053	0.001
**SMU_1828**	*uspA*	universal stress protein family	1.410	1.086	0.772	0.089
**SMU_183**	*sloB*	manganese ABC transporter permease element	1.810	2.051	1.910	0.108
**SMU_1830c**	-	conserved hypothetical protein	2.374	1.604	1.862	0.000

**SMU_1831**	*aspG*	L-asparaginase	2.038	1.862	2.545	0.000
**SMU_1832**	-	hypothetical protein	2.024	2.167	2.762	0.002
**SMU_1833**	*recG*	ATP-dependent DNA helicase	1.129	0.255	0.226	0.001
**SMU_1834**	*alr*	alanine racemase	0.001	0.001	0.000	0.001
**SMU_1835**	*acpS*	acyl carrier protein synthase	0.253	0.155	0.202	0.001
**SMU_1836**	*aroG*	phospho-2-dehydro-3-deoxyheptonate aldolase (DAHP synthase)	0.000	0.000	0.000	0.001
**SMU_1837**	*aroH*	3-deoxy-7-phosphoheptulonate synthase	0.000	0.000	0.000	0.000
**SMU_1838**	*secA*	preprotein translocase subunit SecA	0.011	0.009	0.008	0.001
**SMU_1839**	*manA*	mannose-6-phosphate isomerase	0.919	0.266	1.253	0.000
**SMU_184**	*sloC*	ABC transporter element, iron predicted binding protein	2.776	3.411	3.676	0.323
**SMU_1840**	*scrK*	fructokinase	1.580	1.224	1.475	0.000
**SMU_1841**	*scrA*	PTS system, sucrose-specific IIABC component	1.960	2.164	2.391	0.002
**SMU_1843**	*scrB*	sucrose-6-phosphate hydrolase	1.423	0.052	1.669	0.002
**SMU_1844**	*scrR*	sucrose operon repressor	1.572	1.007	2.179	0.001
**SMU_1845**	*nusB*	transcription antitermination factor	0.088	0.000	0.001	0.004
**SMU_1846 c**	-	conserved hypothetical protein	0.144	0.011	0.000	0.001
**SMU_1847**	*efp*	translation elongation factor P	0.777	0.073	0.050	0.000
**SMU_1848**	-	hypothetical protein (similar to eukaryotic potassium channel proteins)	4.380	4.824	5.534	0.001
**SMU_1849**	*comE B*	deoxycytidylate deaminase; late competence protein	3.292	2.091	2.574	0.000
**SMU_185**	-	hypothetical protein	3.592	4.451	4.998	0.001
**SMU_1850**	*pepP*	aminopeptidase P (XAA-Pro aminopeptidase)	2.553	1.747	1.747	0.001
**SMU_1851**	*uvrA*	excinuclease ABC subunit A	1.556	0.898	0.862	0.009
**SMU_1852**	-	cation transporter, CorA family	0.000	0.001	0.000	0.002
**SMU_1853**	-	conserved hypothetical protein	0.544	0.380	0.207	0.001
**SMU_1854**	*hdrR*	high density responsive regulator, HdrR	1.176	1.414	1.598	0.441
**SMU_1855**	*hdrM*	high density responsive membrane protein, HdrM	0.562	0.162	0.168	0.000
**SMU_1856 c**	-	conserved hypothetical protein	2.877	3.391	3.395	0.006
**SMU_1858**	*rpsR*	30S ribosomal protein S18	0.035	0.000	0.000	0.000
**SMU_1859**	*ssb*	single-stranded DNA-binding protein	0.000	0.000	0.000	0.000
**SMU_186**	*sloR*	metal-dependent transcriptional regulator (probable DtxR homolog)	1.197	0.589	0.039	0.000
**SMU_1860**	*rpsF*	30S ribosomal protein S6	0.000	0.000	0.000	0.000
**SMU_1861 c**	-	hypothetical protein	2.723	2.951	2.625	0.000
**SMU_1862**	-	hypothetical protein	3.799	4.455	3.132	40.223
**SMU_1865**	*mutY*	A/G-specific adenine glycosylase	2.611	2.742	1.876	0.057

**SMU_1867c**	*adhB*	alcohol dehydrogenase	2.018	1.493	0.721	0.000
**SMU_1869**	*trxA*	thioredoxin	2.212	0.410	0.372	0.000
**SMU_1870**	*mutS2*	DNA mismatch repair protein	1.613	0.798	0.509	0.001
**SMU_1871c**	-	conserved hypothetical protein	1.715	0.066	0.171	0.004
**SMU_1872c**	-	Streptococcus-specific protein	3.882	0.361	0.089	0.000
**SMU_1873**	*rnh3*	ribonuclease HIII	0.340	0.207	0.015	0.001
**SMU_1874**	*lepC*	signal peptidase I	0.083	0.113	0.017	0.001
**SMU_1875**	*recD*	exodeoxyribonuclease V alpha subunit	1.187	0.562	0.396	0.002
**SMU_1876**	-	conserved hypothetical protein	3.081	2.346	4.563	0.003
**SMU_1877**	*manL*	mannose PTS system component IIAB	1.011	0.332	0.002	0.001
**SMU_1878**	*manM*	mannose PTS system component IIC	1.557	0.578	0.000	0.003
**SMU_1879**	*manN*	mannose PTS system component IID	3.347	1.347	0.002	0.066
**SMU_187c**	*dusA*	probable tRNA-dihydrouridine synthase	0.818	0.748	1.025	0.000
**SMU_1881c**	*comA*	competence factor transporting ATP-binding / permease protein	2.363	2.759	3.292	0.045
**SMU_1882c**	-	hypothetical protein	7.330	9.209	11.166	0.001
**SMU_1883**	-	conserved hypothetical protein	1.475	0.549	0.074	0.000
**SMU_1884c**	-	conserved hypothetical protein	2.420	3.653	3.510	0.279
**SMU_1886**	*sys*	seryl-tRNA synthetase	0.000	0.000	0.000	0.001
**SMU_1888**	-	transposase fragment	0.838	1.351	1.906	1.192
**SMU_1889c**	-	hypothetical protein (possible relation to bacteriocin BlpU)	2.768	2.215	3.388	2.195
**SMU_188c**	*hslO*	Hsp33-like chaperonin	0.490	0.454	0.501	0.000
**SMU_189**	-	hypothetical protein	0.560	0.873	0.856	0.000
**SMU_1891c**	-	hypothetical protein	1.772	2.065	4.515	0.008
**SMU_1892c**	-	hypothetical protein	1.933	2.808	2.640	0.000
**SMU_1895c**	-	hypothetical protein	6.135	4.711	3.982	0.001
**SMU_1896c**	-	hypothetical protein	2.238	2.394	3.028	0.000
**SMU_1897**	-	ABC transporter, ATP-binding protein; similar to BlpA	2.298	2.093	2.320	0.000
**SMU_1898**	-	ABC transporter, ATP-binding and permease element	1.406	1.483	1.465	0.520
**SMU_1900**	*comB*	ABC transporter	0.668	0.758	0.940	0.012
**SMU_1902c**	-	hypothetical protein	0.833	1.200	1.697	0.001
**SMU_1903c**	-	hypothetical protein	6.828	10.036	11.250	0.004
**SMU_1904c**	-	hypothetical protein	1.835	2.573	2.833	0.004
**SMU_1905c**	-	hypothetical protein	0.866	1.097	2.064	0.000
**SMU_1906c**	-	bacteriocin-related protein	1.302	1.084	1.264	0.000

**SMU_1907**	-	hypothetical protein	0.426	0.394	0.732	0.000
**SMU_1908c**	-	hypothetical protein	5.630	6.844	8.017	0.001
**SMU_1909c**	-	hypothetical protein	4.421	5.183	9.977	0.001
**SMU_1910c**	-	hypothetical protein	2.358	3.302	4.730	0.391
**SMU_1912c**	-	hypothetical protein	0.563	1.335	1.224	0.001
**SMU_1913c**	-	hypothetical protein; immunity protein, BLpL-like	0.718	1.097	1.975	0.001
**SMU_1914c**	*cipB*	bacteriocin protein, BlpO-like	7.591	6.511	5.328	0.000
**SMU_1915**	*comC*	S. mutans specific competence stimulating peptide, precursor	1.463	3.331	1.904	0.000
**SMU_1916**	*comD*	histidine kinase	2.763	1.779	1.802	0.000
**SMU_1917**	*comE*	response regulator	1.157	0.465	0.579	0.000
**SMU_1918**	*dedA*	membrane-associated protein DedA	0.616	0.405	0.116	0.001
**SMU_1919**	*sapR2*	sakacin A production response regulator	0.858	1.153	5.529	0.001
**SMU_191c**	-	phage-related integrase	3.996	4.677	4.557	0.001
**SMU_1920**	*pgdA*	phosphoglycerate dehydrogenase	0.007	0.012	0.014	0.004
**SMU_1921**	*dnaI*	DNA replication protein; primosome component	0.004	0.000	0.000	0.000
**SMU_1922**	*dnaB*	chromosome replication initiation and membrane attachment protein	0.000	0.001	0.001	0.000
**SMU_1923c**	-	conserved hypothetical protein	0.309	0.106	0.072	0.001
**SMU_1924**	*gcrR*	response regulator for glucan-binding protein	0.397	0.132	0.705	0.001
**SMU_1925c**	-	conserved hypothetical protein	2.075	0.640	0.760	0.003
**SMU_1926**	*psaR*	transcriptional regulator PsaR	1.253	1.173	1.422	0.000
**SMU_1927**	*psaA*	PsaA protein/ ABC transporter, ATP-binding protein	1.561	1.886	1.624	0.000
**SMU_1928**	*psaB*	protein secretion ABC transport permease	1.468	1.808	1.912	0.011
**SMU_1929**	*htpX*	protease HtpX-like protein; heat shock protein	1.245	1.635	1.554	0.001
**SMU_1930**	*lemA*	LemA-like protein	1.268	1.609	2.012	0.000
**SMU_1931**	*gidB*	glucose-inhibited division protein	1.035	0.803	1.273	0.000
**SMU_1933c**	*sdcBB*	cobalt ABC transporter permease	1.772	2.935	3.191	0.012
**SMU_1934c**	*sdcBA*	cation ABC transport ATP-binding protein	1.527	2.289	1.878	3.201
**SMU_1935c**	-	conserved hypothetical protein	2.718	2.300	2.024	0.003
**SMU_1936c**	-	conserved hypothetical protein	1.554	2.492	1.505	0.020
**SMU_1937**	*cnhA*	hydrolase, carbon-nitrogen family	0.827	0.688	0.788	0.000
**SMU_1938c**	*atmE*	amino acid ABC transport permease	0.753	0.791	0.436	0.001

**SMU_1939c**	*atmD*	amino acid ABC transport ATP-binding protein	0.782	0.703	0.242	0.000
**SMU_193c**	-	conserved hypothetical protein	0.529	1.302	1.187	0.000
**SMU_1940c**	*atmC*	peptidase, possible succinyl-diaminopimelic descuccinlyase	1.495	0.907	1.137	0.001
**SMU_1941**	*atmB*	ABC transporter solute-binding protein	1.530	0.203	0.579	0.000
**SMU_1942c**	*atmA*	amino acid ABC transporter substrate-binding protein	1.016	0.439	1.089	0.001
**SMU_1943**	*leuRS*	leucyl-tRNA synthetase	0.004	0.001	0.000	0.000
**SMU_1945**	-	conserved hypothetical protein	1.739	1.404	1.220	0.001
**SMU_1946**	-	conserved hypothetical protein	3.691	4.134	4.006	3.793
**SMU_1947**	*nusG*	transcription antitermination factor	0.240	0.010	0.021	0.001
**SMU_1948**	*secE*	preprotein translocase, SecE subunit	0.000	0.001	0.001	0.001
**SMU_1949**	*pbp2a*	penicillin-binding protein 2a	1.009	0.535	0.149	0.000
**SMU_194c**	-	conserved hypothetical protein, phage-related	1.338	0.843	1.393	0.001
**SMU_1950**	*rluD*	ribosomal large subunit pseudouridine synthase D	1.993	1.703	1.591	0.001
**SMU_1951c**	-	conserved hypothetical protein	2.242	1.924	2.229	0.288
**SMU_1954**	*groEL*	chaperonin GroEL	0.000	0.001	0.001	0.001
**SMU_1955**	*groES*	co-chaperonin 10kDa	0.114	0.032	0.042	0.002
**SMU_1956c**	-	conserved hypothetical protein	1.508	1.998	2.029	0.001
**SMU_1957**	*levG*	fructose-specific Enzyme IID component	1.010	0.894	0.949	0.000
**SMU_1958c**	*levF*	fructose-specific Enzyme IIC component	2.176	2.174	1.775	0.001
**SMU_195c**	-	hypothetical protein	1.980	3.183	1.909	0.000
**SMU_1960c**	*levE*	fructose-specific Enzyme IIB component	0.639	0.717	0.539	0.001
**SMU_1961c**	*levD*	fructose-specific Enzyme IIA component	1.164	0.845	0.568	0.001
**SMU_1963c**	*levQ*	sugar-binding periplasmic protein	1.655	3.080	2.649	0.001
**SMU_1964c**	*levR*	two-component response regulator	0.944	1.900	1.408	1.515
**SMU_1965c**	*levS*	histidine kinase	1.518	1.780	1.551	0.001
**SMU_1966c**	*rbsB*	ABC transport ribose-binding protein, periplasmic	1.452	1.756	1.668	0.059
**SMU_1967**	*ssbA*	single-stranded DNA-binding protein	0.717	1.028	0.999	0.000
**SMU_1968c**	-	conserved hypothetical protein	1.023	1.556	1.344	0.000
**SMU_1969c**	-	probable transcriptional regulator	1.259	1.417	1.287	0.001
**SMU_196c**	-	immunogenic secreted protein (transfer protein)	1.544	1.415	1.631	0.072
**SMU_1970c**	*pheT*	phenylalanyl-tRNA synthetase beta subunit	1.039	1.421	0.803	0.001

**SMU_1971c**	*trxH*	thioredoxin family protein	0.383	0.348	0.337	0.001
**SMU_1972c**	-	conserved hypothetical protein	0.918	0.855	0.713	0.001
**SMU_1973**	*pepA*	glutamyl aminopeptidase	1.206	0.436	1.298	0.001
**SMU_1974**	*proC*	pyrroline-5-carboxylate reductase	1.168	0.005	1.231	0.000
**SMU_1975c**	-	conserved hypothetical protein	0.661	0.556	0.455	0.003
**SMU_1976c**	-	conserved hypothetical protein	0.926	1.431	1.295	0.002
**SMU_1977c**	-	transcriptional regulator	0.267	0.177	0.100	0.000
**SMU_1978**	*ackA*	acetate kinase	0.044	0.015	0.008	0.001
**SMU_1979c**	*ythI*	conserved hypothetical protein, methyltransferase domain	0.349	0.273	0.214	0.001
**SMU_197c**	-	hypothetical protein	1.881	2.226	3.729	0.128
**SMU_1980c**	-	conserved hypothetical protein	0.598	0.706	0.559	0.000
**SMU_1981c**	*comG*	competence protein G	0.356	0.613	0.386	0.002
**SMU_1982c**	-	conserved hypothetical protein	0.294	0.296	0.275	0.000
**SMU_1983**	*comYD*	competence protein ComYD	1.092	1.332	1.185	0.001
**SMU_1984**	*comYC*	competence protein ComYC	0.477	0.595	0.467	0.002
**SMU_1985**	*comYB*	competence protein; general (type II) secretory pathway protein	0.299	0.255	0.238	0.037
**SMU_1987**	*comYA*	late competence protein; type II secretion system protein E	0.589	0.356	0.367	0.001
**SMU_1988c**	-	probable DNA binding protein	0.388	0.565	0.081	0.001
**SMU_1989**	*rpoC*	DNA-directed RNA polymerase, beta' subunit	0.028	0.015	0.026	0.005
**SMU_198c**	*tpn*	conjugative transposon protein	3.995	5.740	8.983	0.006
**SMU_1990**	*rpoB*	DNA-directed RNA polymerase, beta subunit	0.035	0.011	0.023	0.003
**SMU_1991**	*pbp1b*	penicillin-binding protein 1b	1.868	0.061	0.228	0.005
**SMU_1992**	*tyrS*	tyrosyl-tRNA synthetase	0.079	0.026	0.033	0.000
**SMU_1993**	*adcB*	zinc ABC transporter, permease	0.489	0.061	0.002	0.003
**SMU_1994**	*adcC*	zinc ABC transport, ATP-binding protein	0.341	0.063	0.003	0.000
**SMU_1995c**	*zitR*	zinc transport transcriptional repressor	0.270	0.102	0.021	0.000
**SMU_1996**	*ipk*	isopentenyl monophosphate kinase (4-diphosphocytidyl-2-C-methyl-D-erythritol kinase)	2.804	2.752	2.752	0.472
**SMU_1997**	*comX1*	competence-specific sigma factor	1.521	1.584	1.963	45.863
**SMU_1999c**	-	glutamate--cysteine ligase	0.730	0.854	0.384	0.003
**SMU_199c**	-	hypothetical protein	5.406	6.616	11.323	0.003
**SMU_20**	*mreC*	cell shape-determining protein MreC	1.145	8.024	12.768	0.003
**SMU_2000**	*rl17*	50S ribosomal protein L17	0.002	0.001	0.001	0.001

**SMU_2001**	*rpoA*	DNA-directed RNA polymerase, alpha subunit	0.000	0.001	0.000	0.000
**SMU_2002**	*rs11*	30S ribosomal protein S11	0.046	0.022	0.041	0.001
**SMU_2003**	*rs13*	50S ribosomal protein L36	0.044	0.002	0.000	0.000
**SMU_2004**	*if1*	translation initiation factor IF-1	0.001	0.000	0.000	0.000
**SMU_2005**	*adk*	adenylate kinase (ATP-AMP transphosphorylase) (superoxide-inducible protein 16)	0.001	0.001	0.000	0.000
**SMU_2006**	*secY*	preprotein translocase	0.005	0.007	0.016	0.008
**SMU_2007**	*rl15*	50S ribosomal protein L15, N-terminal fragment	0.004	0.000	0.000	0.000
**SMU_2008**	*rl30*	50S ribosomal protein L30	0.001	0.000	0.000	0.046
**SMU_2009**	*rs5*	30S ribosomal protein S5	0.017	0.000	0.000	0.000
**SMU_200c**	-	hypothetical protein	2.259	2.852	3.716	0.001
**SMU_2010**	*rl18*	50S ribosomal protein L18	0.001	0.000	0.000	0.000
**SMU_2011**	*rl6*	50S ribosomal protein L6 (BL10)	0.012	0.003	0.008	0.000
**SMU_2012**	*rs8*	30S ribosomal protein S8	0.000	0.001	0.000	0.000
**SMU_2014**	*rs14*	30S ribosomal protein S14	0.000	0.000	0.000	0.000
**SMU_2015**	*rl5*	50S ribosomal protein L5	0.000	0.000	0.000	0.002
**SMU_2016**	*rl24*	50S ribosomal protein L24	0.000	0.001	0.000	0.000
**SMU_2017**	*rl14*	50S ribosomal protein L14	0.001	0.000	0.000	0.000
**SMU_2018**	*rs17*	30S ribosomal protein S17	0.000	0.000	0.000	0.000
**SMU_2019**	*rl29*	50s ribosomal protein L29	0.001	0.000	0.000	0.000
**SMU_201c**	-	conserved hypothetical protein	2.414	2.264	3.024	0.001
**SMU_2020**	*rl16*	50S ribosomal protein L16	0.000	0.000	0.000	0.000
**SMU_2021**	*rs3*	30S ribosomal protein S3	0.001	0.000	0.000	0.003
**SMU_2022**	*rl22*	50S ribosomal protein L22	0.001	0.000	0.001	0.000
**SMU_2023 c**	*rps19*	30S ribosomal protein S19, C-terminal fragment	0.000	0.001	0.000	0.000
**SMU_2024 c**	*rl4*	50S ribosomal protein L4, N-terminal fragment	0.001	0.001	0.000	0.000
**SMU_2025**	*rl3*	50S ribosomal protein L3	0.000	0.000	0.000	0.000
**SMU_2026 c**	*rps1NA*	30S ribosomal protein S10 fragment	0.126	0.006	0.010	0.003
**SMU_2027**	-	transcriptional regulator/repressor	1.874	2.934	2.907	0.001
**SMU_2028**	*sacB*	fructosyltransferase	2.086	3.044	3.155	0.023
**SMU_2029**	*clpC*	ATP-dependent Clp protease, ATP-binding subunit (Class III stress response-related ATPase)	1.352	1.600	1.122	0.006
**SMU_202c**	-	conserved hypothetical protein/Streptococcus-specific protein	1.476	1.409	1.834	0.000
**SMU_2030**	*ctsR*	transcriptional regulator of class III stress genes, CtsR	0.603	0.468	0.190	0.000
**SMU_2031**	*eftS*	translation elongation factor Ts	0.000	0.000	0.000	0.000
**SMU_2032**	*rs2*	30S ribosomal protein S2	0.002	0.000	0.000	0.001
**SMU_2033 c**	-	conserved hypothetical protein	6.796	7.342	8.271	0.018
**SMU_2035**	*mccF*	possible bacteriocin self-immunity protein	2.163	2.999	2.915	0.002
**SMU_2036**	*pepO*	endopeptidase O	0.725	0.575	0.663	0.001
**SMU_2037**	*treA*	trehalose-6-phosphate hydrolase	0.019	0.002	0.024	0.000

**SMU_2038**	*treB*	phosphotransferase system, trehalose-specific IIBC component (EIIBC-tre)	1.666	1.705	2.257	0.029
**SMU_2040**	*treR*	trehalose operon transcriptional repressor (transcriptional regulator, GntR family)	1.880	2.120	2.280	0.002
**SMU_2042**	*dexT*	dextranase (1,6-alpha-glucanhydrolase)	1.829	2.202	1.894	0.006
**SMU_2043 c**	*dtd*	D-tyrosyl-tRNA deacylase	1.136	1.125	1.377	0.001
**SMU_2044**	*relA*	GTP pyrophosphokinase	0.415	0.018	0.002	0.001
**SMU_2046 c**	-	conserved hypothetical protein	1.413	1.289	1.321	0.000
**SMU_2047**	*ptsG*	PTS system, enzyme II, A component	2.571	3.479	2.922	0.001
**SMU_2048**	-	hypothetical protein	0.365	0.487	0.417	0.002
**SMU_2049 c**	-	conserved hypothetical protein	1.282	1.225	0.946	0.001
**SMU_204c**	-	hypothetical protein	1.293	1.205	1.150	0.000
**SMU_2050 c**	*prmA*	ribosomal protein L11 methylase	2.803	2.446	2.141	0.001
**SMU_2052 c**	-	hypothetical protein	1.449	1.731	1.766	0.001
**SMU_2054 c**	-	conserved hypothetical protein	1.338	1.126	1.229	0.001
**SMU_2055**	-	acetyltransferase	1.427	1.501	1.344	0.000
**SMU_2056**	*cshA*	chromosome segregation helicase (ATPase related to the helicase subunit of the Holliday junction resolvase)	1.290	1.346	1.358	0.000
**SMU_2057 c**	*cadA*	cadmium-efflux ATPase, E1-E2 (heavy metal-transporting ATPase)	2.094	1.239	1.399	0.003
**SMU_2058**	-	transcriptional regulator	1.490	1.706	1.866	0.000
**SMU_2059 c**	-	conserved hypothetical protein; possible membrane protein	1.798	2.303	2.023	0.003
**SMU_205c**	-	conserved hypothetical protein	3.926	4.965	6.474	0.312
**SMU_2060**	*rscR*	transcriptional regulator, LysR family (possible RUBISCO transcriptional regulator, RscR)	2.031	1.962	1.647	6.323
**SMU_2061**	-	hypothetical protein	2.970	2.707	2.493	0.002
**SMU_2063**	*hemZ*	ferrochelatase (heme synthetase) (protoheme ferro-lyase)	2.046	2.327	2.530	0.000
**SMU_2064 c**	-	hypothetical protein	2.807	3.518	3.196	0.054
**SMU_2065**	*galE*	UDP-glucose 4-epimerase	1.657	2.086	2.182	0.001
**SMU_2066 c**	-	conserved hypothetical protein (possible integral membrane protein, ABC transporter)	2.560	4.855	3.379	0.041
**SMU_2067**	*csbB*	stress response protein (glycosyltransferase) (sugar transferase)	1.998	2.186	2.171	0.284

**SMU_2069**	-	conserved hypothetical protein (probable integral membrane protein)	2.248	2.567	2.834	0.004
**SMU_206c**	-	hypothetical protein	1.647	1.399	1.324	0.001
**SMU_2070**	-	conserved hypothetical protein	1.501	1.981	1.934	0.019
**SMU_2071**	*nrdG*	anaerobic ribonucleoside-triphosphate reductase activating protein (NrdD activating enzyme, generating glycyl radical)	1.629	0.893	1.856	0.001
**SMU_2072c**	-	possible acetyltransferase, GNAT family	1.466	1.263	2.006	0.005
**SMU_2073c**	-	conserved hypothetical protein	0.712	0.862	0.664	0.000
**SMU_2074**	*nrdD*	anaerobic ribonucleoside-triphosphate reductase	1.285	0.404	0.992	0.001
**SMU_2075c**	-	conserved hypothetical protein (possible competence-induced protein)	1.535	1.840	2.870	0.002
**SMU_2076c**	-	hypothetical protein	0.783	0.632	0.902	0.000
**SMU_2077c**	-	conserved hypothetical protein	0.470	0.034	0.062	0.001
**SMU_2078c**	-	conserved hypothetical protein; possible Holliday junction resolvase-like protein	0.077	0.002	0.036	0.000
**SMU_2079c**	-	conserved hypothetical protein	0.119	0.091	0.215	0.001
**SMU_207c**	-	transcriptional regulator	1.913	2.081	2.089	0.096
**SMU_2080**	-	conserved hypothetical protein	2.217	1.843	2.111	0.001
**SMU_2081**	-	hypothetical protein	1.885	1.239	2.001	0.003
**SMU_2083c**	-	hypothetical protein	0.962	0.952	1.320	0.009
**SMU_2084c**	-	conserved hypothetical protein (possible arsenate reductase)	0.704	0.176	0.251	0.020
**SMU_2085**	*recA*	recombinase A	0.648	0.014	0.001	0.001
**SMU_2086**	*cinA*	competence damage-inducible protein A	0.855	0.696	0.340	0.000
**SMU_2087**	*tagI*	3-methyladenine DNA glycosylase I	0.703	0.795	0.742	0.002
**SMU_2088**	*ruvA*	Holliday junction DNA helicase	0.432	0.013	0.024	0.001
**SMU_2089**	*hexB*	DNA mismatch repair protein	1.178	1.472	1.717	0.022
**SMU_208c**	-	conserved hypothetical protein, FtsK/SpoIIIE family	1.814	1.866	1.741	0.000
**SMU_2090c**	-	hypothetical protein	0.333	0.119	0.518	0.001
**SMU_2091c**	*hexA*	DNA mismatch repair protein, HexA	1.708	1.465	1.303	0.001
**SMU_2092c**	-	conserved hypothetical protein	1.201	0.777	0.782	0.008
**SMU_2093**	*argR*	arginine repressor	0.006	0.000	0.000	0.001
**SMU_2094c**	*arcE*	transcriptional regulator	4.311	4.389	5.153	1.645
**SMU_2096c**	-	conserved hypothetical protein	1.875	1.301	3.920	0.001

**SMU_2097**	*argR*	arginine transcriptional repressor (arginine hydroxymate resistance protein)	1.695	1.407	1.619	0.794
**SMU_2098**	*argS*	arginyl-tRNA synthetase	0.016	0.035	0.031	0.000
**SMU_2099c**	-	conserved hypothetical protein	1.613	1.149	2.609	0.020
**SMU_209c**	-	hypothetical protein	2.732	3.714	3.431	0.000
**SMU_21**	*mreD*	cell shape-determining protein MreD	6.791	36.699	49.159	0.053
**SMU_2100c**	-	conserved hypothetical protein	3.673	2.768	3.133	221.069
**SMU_2101**	*aspS*	aspartyl-tRNA synthetase	0.640	0.007	0.328	0.000
**SMU_2102**	*hisS*	histidine-tRNA synthetase	0.001	0.001	0.001	0.001
**SMU_2104**	*potE*	aminoacid specific permease	2.089	2.294	1.666	0.000
**SMU_2104a**	*rl32*	50S ribosomal protein L32	0.696	0.049	0.057	0.000
**SMU_2105**	-	hypothetical protein	0.553	0.660	0.730	0.000
**SMU_2106c**	-	hypothetical protein	3.560	5.124	4.908	0.005
**SMU_2107c**	-	hypothetical protein	1.027	0.939	0.937	0.000
**SMU_2108c**	*mepR*	transcriptional regulator, MepR protein (possible regulator of MepA)	2.078	2.900	2.694	0.001
**SMU_2109**	*mepA*	possible multiple drug efflux pump MepA	2.137	2.524	3.009	0.088
**SMU_210c**	-	hypothetical protein	0.563	0.507	0.319	0.000
**SMU_2111c**	*orf1*	hypothetical protein	2.978	4.193	3.663	0.001
**SMU_2112**	*gbpA*	glucan-binding protein	1.081	1.434	1.383	0.054
**SMU_2113c**	-	conserved hypothetical protein	3.860	4.628	5.109	0.002
**SMU_2114c**	*rmeD*	probable transcriptional regulator, MerR family	1.698	2.076	2.260	0.002
**SMU_2115**	-	probable oxidoreductase (probable short-chain dehydrogenase)	2.146	2.831	2.666	0.000
**SMU_2116**	*opuCa*	glycine betaine / carnitine / choline ABC transporter, ATP-binding protein, opuCA	1.902	2.018	1.955	0.155
**SMU_2117**	*opuCb*	glycine betaine / carnitine / choline ABC transporter permease	2.275	2.543	2.331	0.001
**SMU_2118**	*opuCc*	glycine betaine/carnitine/choline ABC transporter, substrate-binding protein	3.328	3.627	4.333	0.000
**SMU_2119**	*opuCd*	ABC transport betaine/carnitine/choline permease	3.360	4.488	4.840	0.002
**SMU_211c**	-	hypothetical protein	6.332	6.836	8.791	0.226
**SMU_2120c**	*3mgh*	probable 3-methyladenine DNA glycosylase	1.955	1.917	2.106	0.000
**SMU_2121c**	-	conserved hypothetical protein	3.624	4.097	4.635	0.000
**SMU_2123**	-	hypothetical protein	5.056	5.763	6.550	0.000
**SMU_2124**	-	hypothetical protein	0.474	1.260	1.763	0.000

**SMU_2125**	-	conserved hypothetical protein (possible transcriptional regulator)	3.149	3.373	3.669	0.000
**SMU_2126c**	-	possible phosphorylase	0.955	1.159	1.167	0.000
**SMU_2127**	*gabD*	succinic semialdehyde dehydrogenase (NAD-dependent aldehyde dehydrogenase)	1.336	1.483	1.523	0.000
**SMU_2128**	*dad*	dihydroxy-acid dehydratase	1.317	0.040	0.000	0.090
**SMU_2129c**	-	conserved hypothetical protein	1.967	1.619	0.562	0.000
**SMU_212c**	-	hypothetical protein	1.267	1.577	1.441	0.000
**SMU_2130**	*shetA*	exfoliative toxin A	1.786	2.054	2.223	0.001
**SMU_2131**	-	hypothetical protein	1.862	2.027	2.271	0.009
**SMU_2133c**	-	conserved hypothetical protein (possible transmembrane protein)	1.582	1.739	1.991	0.083
**SMU_2134**	-	transcriptional regulator, TetR/AcrR family	2.198	3.263	4.962	0.009
**SMU_2135c**	*rpsD*	30S Ribosomal protein S4	0.004	0.001	0.000	0.000
**SMU_2136c**	-	hypothetical protein	0.561	0.468	0.655	0.000
**SMU_2137c**	-	conserved hypothetical protein	1.608	0.015	0.028	0.002
**SMU_2138**	*dnaC*	replicative DNA helicase	0.030	0.063	0.163	0.003
**SMU_2139c**	*rl9*	50S ribosomal protein L9	0.345	0.556	0.587	0.000
**SMU_213c**	-	hypothetical protein	5.728	6.520	8.098	0.002
**SMU_2142**	*rpiA*	sugar-phosphate isomerase (ribose 5-phosphate isomerase)	0.173	0.140	0.231	0.002
**SMU_2143c**	*trmU*	tRNA (5-methylaminomethyl-2-thiouridylate)-methyltransferase	0.003	0.003	0.001	0.001
**SMU_2146c**	-	conserved hypothetical protein	0.876	0.302	0.559	0.002
**SMU_2147c**	-	conserved hypothetical protein	0.739	1.001	0.552	0.001
**SMU_2148c**	-	ABC transporter, membrane-spanning permease	0.037	0.029	0.002	0.007
**SMU_2149c**	-	ABC transporter, ATP-binding protein	0.003	0.000	0.001	0.006
**SMU_214c**	-	hypothetical protein	1.498	2.842	3.979	0.002
**SMU_2150c**	-	ABC transporter, ATP-binding protein	0.015	0.018	0.004	0.000
**SMU_2151**	*pgsA*	CDP-diacylglycerol--glycerol-3-phosphate 3-phosphatidyltransferase	0.000	0.000	0.000	0.001
**SMU_2152c**	*rodZ*	conserved hypothetical protein	1.093	2.377	8.430	0.001
**SMU_2153c**	-	peptidase, M16 family	5.683	5.252	7.097	0.001
**SMU_2154c**	-	peptidase, M16 family	2.772	2.100	2.791	0.002
**SMU_2155**	-	conserved hypothetical protein	0.670	0.373	0.552	0.002
**SMU_2156**	*recF*	recombination protein F (DNA replication and repair ATPase)	0.633	0.061	0.102	0.001

**SMU_2157**	*guaB*	inosine-5'- monophosphate dehydrogenase	0.346	0.036	0.134	0.002
**SMU_2158c**	*trsA*	tryptophanyl-tRNA synthetase	0.004	0.007	0.018	0.001
**SMU_2159**	-	ABC transporter, ATP-binding protein	2.234	1.728	2.089	0.011
**SMU_215c**	-	hypothetical protein	0.593	0.814	0.646	0.000
**SMU_2160**	-	possible permease, ABC transporter protein	4.825	5.531	6.264	0.010
**SMU_2161c**	-	conserved hypothetical protein	12.958	14.802	14.508	0.051
**SMU_2162c**	-	conserved hypothetical protein	0.561	0.383	0.268	0.000
**SMU_2164**	*htrA*	serine protease	1.725	1.745	1.635	0.002
**SMU_2165**	*smc*	chromosome segregation protein, ParB-like nuclease domain	2.073	0.326	0.630	0.000
**SMU_2166**	*rplW*	Ribosomal protein L23	0.000	0.000	0.000	0.000
**SMU_2167**	*rplB*	Ribosomal protein L2	0.012	0.000	0.000	0.001
**SMU_216c**	-	hypothetical protein	2.942	5.148	4.982	0.001
**SMU_217c**	-	conserved hypothetical protein; Streptococcus-specific protein	3.294	4.024	4.112	0.000
**SMU_218**	-	transcriptional regulator	0.066	0.001	0.003	0.017
**SMU_219**	-	hypothetical protein	3.228	3.660	2.340	0.001
**SMU_22**	*gbpB*	glucan-binding protein B	0.000	0.000	0.000	0.000
**SMU_220c**	-	hypothetical protein	7.753	7.561	12.199	0.002
**SMU_221c**	-	phage-related integrase, truncated	5.786	6.264	5.992	0.002
**SMU_222c**	-	hypothetical protein (possible integrase fragment)	1.574	1.912	2.147	0.000
**SMU_223c**	-	hypothetical protein	2.037	2.127	1.594	0.000
**SMU_224c**	-	hypothetical protein	3.685	3.431	4.174	0.002
**SMU_225c**	-	hypothetical protein	1.029	2.096	3.096	0.002
**SMU_226c**	*tpn*	transposase, IS1216, also reported on plasmid, truncated	1.189	1.432	0.997	0.000
**SMU_227c**	-	conserved hypothetical protein	8.658	13.425	8.470	0.011
**SMU_228**	-	alkaline-shock protein homolog	0.125	0.010	0.037	0.021
**SMU_229**	-	conserved hypothetical protein (possible kinase)	0.262	0.001	0.232	0.000
**SMU_23**	*prs*	phosphoribosyl pyrophosphate synthetase (PRPP synthetase)	0.002	0.000	0.001	0.001
**SMU_231**	*ilvB*	acetolactate synthase, large subunit (AHAS)	0.972	0.053	0.018	0.001
**SMU_232**	*ilvH*	acetolactate synthase, small subunit	1.509	0.589	0.954	0.000
**SMU_233**	*ilvC*	ketol-acid reductoisomerase	0.933	0.042	0.000	0.000
**SMU_234**	*ilvA*	threonine dehydratase	1.576	1.678	0.969	0.000
**SMU_235**	-	conserved hypothetical protein, SPFH domain/band 7 family	1.947	2.423	1.185	0.002
**SMU_236c**	*ycdC*	transcriptional regulator	0.908	0.409	0.405	0.000
**SMU_237c**	*drrB*	ABC transporter multidrug permease protein	3.160	2.737	2.708	0.003
**SMU_238c**	-	ABC transporter, ATP-binding protein	1.721	1.288	1.477	0.002

**SMU_239c**	-	conserved hypothetical protein	2.586	2.690	3.648	0.278
**SMU_24**	*aspC*	aspartate or aromatic amino acid aminotransferase	0.010	0.031	0.069	0.038
**SMU_241c**	-	amino acid ABC transporter, ATP-binding protein	0.688	0.097	0.303	0.000
**SMU_242c**	-	glutamine ABC transporter, solute binding protein	3.979	0.468	1.475	0.002
**SMU_243**	-	conserved hypothetical protein	0.814	0.631	0.496	0.124
**SMU_244**	*bacA*	undecaprenyl-diphosphatase; bacitracin resistance protein	0.080	0.036	0.037	0.001
**SMU_245**	*mecA*	negative regulator of genetic competence	0.426	4.722	0.084	0.005
**SMU_246**	*rgpG*	polysaccharide biosynthesis protein, glycosyl transferase family 4	0.050	0.277	0.001	0.002
**SMU_247**	*sufC*	ABC transporter, ATP-binding protein	0.788	0.003	0.000	0.000
**SMU_248**	-	ABC transporter permease	1.148	0.005	0.000	0.000
**SMU_249**	*nifS*	class-V aminotransferase, NifS protein homolog,	1.433	0.007	0.002	0.000
**SMU_25**	*recO*	DNA repair protein RecO (recombination protein O)	1.012	0.021	0.013	0.000
**SMU_250**	*nifU*	nitrogen fixation-like protein, NifU	0.804	0.004	0.000	0.010
**SMU_251**	-	ABC transporter permease	1.026	0.008	0.020	0.000
**SMU_252**	-	hypothetical protein	1.907	1.884	1.637	0.000
**SMU_253**	*dacA*	D-alanyl-D-alanine carboxypeptidase; penicillin-binding protein	1.667	2.242	3.244	0.001
**SMU_255**	*oppA*	oligopeptide ABC transporter, OppA	1.888	1.462	1.964	145.914
**SMU_256**	*oppB*	oligopeptide ABC transporter, permease	1.487	0.794	1.219	2.527
**SMU_257**	*oppC*	oligopeptide ABC transporter, permease	1.663	1.136	2.012	253.083
**SMU_258**	*oppD*	oligopeptide ABC transporter, ATP-binding	1.285	0.915	1.404	105.258
**SMU_259**	*oppF*	oligopeptide ABC transporter, ATP-binding	1.181	1.047	1.013	57.069
**SMU_26**	*plsX*	fatty acid/phospholipid synthesis protein	0.194	0.005	0.003	0.001
**SMU_260**	-	conserved hypothetical protein	1.559	2.104	2.858	5.102
**SMU_261c**	*aguR*	transcriptional regulator	2.635	2.582	3.112	0.001
**SMU_262**	*otcA*	ornithine carbamoyltransferase	1.727	1.453	1.748	0.427
**SMU_263**	*aguD*	amino acid permease /putrescine antiporter	1.477	1.715	1.910	0.001
**SMU_264**	*aguA*	agmatine deiminase	1.647	1.559	1.711	0.000
**SMU_265**	*arcC*	carbamate kinase	1.370	1.258	1.324	0.000
**SMU_267c**	*gcl*	glutamate--cysteine ligase (gamma ECS)	2.047	2.640	1.837	0.069
**SMU_268**	*purA*	adenylosuccinate synthetase	0.921	0.144	0.680	0.004
**SMU_27**	*acpP*	acyl carrier protein	0.107	0.021	0.106	0.000
**SMU_270**	*sgaT*	ribulose monophosphate PTS pathway enzyme IIC	1.907	1.474	1.893	0.001

**SMU_271**	*ptxB*	PTS system, enzyme IIB component	2.443	3.026	4.997	0.000
**SMU_272**	*ptxA*	PTS system, enzyme IIA component	1.333	1.168	1.402	0.012
**SMU_273**	*rmpD*	hexulose-6-phosphate synthase	1.459	1.229	1.994	0.001
**SMU_274**	*rmpE*	hexulose-6-phosphate isomerase	1.604	2.710	2.395	0.001
**SMU_275**	*rmpF*	L-ribulose 5-phosphate 4-epimerase	1.660	2.090	2.122	0.000
**SMU_276c**	-	hypothetical protein	3.558	3.518	4.338	0.011
**SMU_277**	-	hypothetical protein	6.000	5.888	7.272	0.481
**SMU_278**	-	hypothetical protein	5.216	5.496	4.377	0.000
**SMU_279**	-	hypothetical protein	25.453	28.141	29.435	0.001
**SMU_28**	*comA*	ABC transporter, ATP-binding protein	1.270	1.173	1.102	0.000
**SMU_281**	-	hypothetical protein	1.770	1.571	1.298	0.000
**SMU_283**	-	hypothetical protein	2.859	4.061	4.298	0.000
**SMU_284**	-	hypothetical protein	3.107	3.747	4.468	0.009
**SMU_286**	*comA*	competence factor transporting ATP-binding/permease protein	1.874	2.566	4.778	0.110
**SMU_287**	*comB*	ABC transport protein ComB, accessory factor for ComA	0.793	1.194	1.206	0.213
**SMU_289**	-	transcriptional antiterminator	3.305	3.805	5.518	0.001
**SMU_29**	*purC*	phosphoribosylaminoimidazole-succinocarboxamide synthase	1.003	0.540	0.659	0.037
**SMU_290**	-	conserved hypothetical protein	2.439	2.693	3.038	0.000
**SMU_291**	*tkt*	transketolase	0.012	0.000	0.000	0.000
**SMU_292**	-	transcriptional regulator, probable AraC family	1.440	0.954	0.817	0.002
**SMU_293**	-	hypothetical protein	2.208	1.657	1.333	0.000
**SMU_294**	-	conserved hypothetical protein	2.003	2.587	2.383	0.000
**SMU_295**	-	hypothetical protein	1.396	1.213	1.385	0.002
**SMU_296**	-	conserved hypothetical protein	2.599	1.957	2.011	0.001
**SMU_297**	*polI*	DNA polymerase I	0.159	0.063	0.046	0.000
**SMU_298**	-	conserved hypothetical protein	0.403	0.322	0.447	0.000
**SMU_299c**	*ip*	bacteriocin peptide precursor	2.855	4.712	3.563	0.000
**SMU_30**	*purL*	phosphoribosylformylglycinamidine synthase	1.218	0.793	0.665	0.012
**SMU_300**	*tgt*	tRNA-guanine transglycosylase; queuine tRNA-ribosyltransferase	1.146	1.050	0.887	0.113
**SMU_301**	-	conserved hypothetical protein	1.046	1.109	1.008	0.001
**SMU_302**	-	conserved hypothetical protein	2.716	2.741	3.294	1.379
**SMU_303**	-	conserved hypothetical protein	1.021	0.633	0.848	0.189
**SMU_304**	*cdd*	cytidine/ deoxycytidylate deaminase	0.698	0.002	0.000	0.000
**SMU_305**	-	conserved hypothetical protein	1.704	1.776	1.512	19.892
**SMU_307**	*pgi*	glucose-6-phosphate isomerase	0.000	0.000	0.000	0.001
**SMU_308**	*srlD*	sorbitol-6-phosphate 2-dehydrogenase	0.823	0.457	0.378	0.001
**SMU_309**	*srlR*	regulator of sorbitol operon	1.460	0.827	1.173	0.000
**SMU_31**	-	conserved hypothetical protein	1.558	1.586	1.552	0.000
**SMU_310**	*srlM*	sorbitol operon activator	2.960	1.729	3.089	0.001
**SMU_311**	*srlA*	PTS system, sorbitol (glucitol) phosphotransferase enzyme IIC2	1.702	1.779	1.826	8.563

**SMU_312**	*srlE*	PTS system, sorbitol phosphotransferase enzyme IIBC	1.099	1.074	1.016	0.001
**SMU_313**	*srlB*	PTS system, sorbitol-specific enzyme IIA	2.561	2.131	1.771	0.000
**SMU_314**	-	hypothetical protein	2.736	2.857	2.888	0.001
**SMU_317**	*dapD*	tetrahydrodipicolinate succinylase	0.264	0.001	0.000	0.000
**SMU_318**	*hipO*	peptidase/aminoacylase/hippurate hydrolase	1.692	0.945	0.528	0.003
**SMU_32**	*purF*	amidophosphoribosyltransferase	1.339	1.412	1.331	0.107
**SMU_320**	-	probable 5-formyltetrahydrofolate cyclo-ligase	0.996	1.418	0.999	0.000
**SMU_321**	-	conserved hypothetical protein, Rhomboid family	0.570	0.487	0.486	0.001
**SMU_322c**	*galU*	glucose-1-phosphate uridylyltransferase	0.000	0.000	0.000	0.001
**SMU_323**	*gpsA*	glycerol-3-phosphate dehydrogenase (NAD(P)+)	0.000	0.000	0.000	0.002
**SMU_325**	*dutA*	dUTP diphosphatase (dUTPase)	0.387	0.029	0.002	0.001
**SMU_326**	-	conserved hypothetical protein	2.044	2.279	1.555	0.001
**SMU_327**	*radA*	DNA repair protein	1.135	0.978	0.657	0.004
**SMU_328**	-	conserved hypothetical protein; possible carbonic anhydrase	2.507	0.002	1.160	0.001
**SMU_329**	-	conserved hypothetical protein	1.063	0.495	1.086	2.477
**SMU_33**	-	hypothetical protein	1.112	1.713	1.998	0.000
**SMU_330**	*gltX*	glutamyl-tRNA synthetase	0.000	0.000	0.000	0.000
**SMU_331**	-	transcriptional regulator	0.210	0.171	0.159	0.000
**SMU_332**	-	conserved hypothetical protein	1.383	1.012	0.974	0.011
**SMU_333**	-	conserved hypothetical protein	1.943	0.958	1.350	0.003
**SMU_334**	*argG*	argininosuccinate synthase	1.938	1.880	1.820	0.000
**SMU_335**	*argH*	argininosuccinate lyase	2.125	2.166	2.401	0.139
**SMU_336**	*rnpA*	ribonuclease P protein component	0.529	1.777	0.586	0.001
**SMU_337**	*yidC1*	inner membrane protein	1.299	0.850	0.216	0.001
**SMU_338**	*jag*	conserved hypothetical protein, Jag protein	1.059	0.084	0.111	0.001
**SMU_339**	-	hypothetical protein	2.676	1.920	1.615	0.002
**SMU_34**	*purM*	phosphoribosylformylglycinamide cyclo-ligase (AIRS)	1.088	0.802	0.716	0.000
**SMU_340**	*rpmH*	50S ribosomal protein L34	0.092	0.000	0.000	0.000
**SMU_341**	*yabD*	conserved hypothetical protein (possible deoxyribonuclease)	2.208	1.325	1.241	0.203
**SMU_342**	-	possible primase-related protein	0.985	0.100	0.138	0.001
**SMU_343**	-	conserved hypothetical protein	1.200	0.852	0.907	0.002
**SMU_344**	-	hypothetical protein	0.781	0.215	0.105	0.004
**SMU_345c**	-	conserved hypothetical protein (possible transcription regulator)	0.524	0.708	0.435	0.000
**SMU_346**	*nrd*	NADH dehydrogenase; NAD(P)H nitroreductase	0.993	0.551	0.549	0.003
**SMU_348**	*pht*	histidine triad (HIT) hydrolase	0.453	0.168	0.169	0.000
**SMU_349**	*ksgA*	dimethyladenosine transferase	0.648	0.223	0.114	0.001
**SMU_35**	*purN*	phosphoribosylglycinamide formyltransferase	0.994	0.896	0.876	0.001

**SMU_350**	-	hypothetical protein	0.594	0.403	0.466	0.001
**SMU_351**	-	conserved hypothetical protein, possible GTPase	1.368	0.056	0.130	0.002
**SMU_352**	*rpe*	ribulose-phosphate-3-epimerase	0.016	0.001	0.000	0.001
**SMU_353**	-	thiamine pyrophosphokinase	0.022	0.000	0.000	0.001
**SMU_354**	-	conserved hypothetical protein	0.721	0.421	0.608	0.001
**SMU_355**	*cbf1*	CMP-binding factor 1	1.022	0.709	0.584	1.966
**SMU_356**	*purR*	purine operon repressor/xanthine phosphoribosyltransferase	0.607	0.286	0.196	0.000
**SMU_357**	*rpsL*	30S ribosomal protein S12	0.000	0.000	0.000	0.000
**SMU_358**	*rpsG*	30S ribosomal protein S7	0.000	0.000	0.000	0.000
**SMU_359**	*efg*	translation elongation factor G/EF-2	0.001	0.000	0.000	0.000
**SMU_36**	-	conserved hypothetical protein (eukaryotic-like)	2.178	2.184	2.232	0.000
**SMU_360**	*gapC*	glyceraldehyde-3-phosphate dehydrogenase; plasmin receptor	0.000	0.000	0.000	0.002
**SMU_361**	*pgk*	phosphoglycerate kinase	0.001	0.001	0.000	0.000
**SMU_362**	-	Streptococcus-specific protein	3.312	2.150	1.557	0.012
**SMU_363**	*glnR*	transcriptional regulator; glutamine synthetase repressor	0.882	0.233	0.093	0.001
**SMU_364**	*glnA*	glutamine synthetase type 1	4.083	0.001	0.001	0.000
**SMU_365**	*gltA*	glutamate synthase, large subunit	1.195	1.291	1.352	0.040
**SMU_366**	*gltB*	glutamate synthase, small subunit	1.391	1.247	0.985	0.008
**SMU_367**	-	Streptococcus-specific protein; similar to glucan-binding protein	1.915	2.018	1.987	0.001
**SMU_368c**	-	metallo-beta-lactamase superfamily protein	0.002	0.000	0.002	0.002
**SMU_369c**	-	conserved hypothetical protein	0.037	0.000	0.002	0.000
**SMU_37**	*purH*	phosphoribosylaminoimidazolecarboxamide formyltransferase / IMP cyclohydrolase	1.273	1.349	0.926	0.000
**SMU_370**	-	ABC transporter, ATP-binding protein	2.709	1.699	1.435	0.002
**SMU_371**	-	Streptococcus-specific protein; possible ABC transporter, membrane spanning permease	2.329	2.391	2.821	0.001
**SMU_372**	-	hypothetical protein	3.390	4.377	5.079	0.005
**SMU_373**	-	hypothetical protein	2.155	2.575	3.259	7.073
**SMU_374**	-	oxidoreductase/ dehydrogenase	2.533	2.546	2.604	0.001
**SMU_375**	-	hypothetical protein	5.595	6.942	6.910	113.365
**SMU_376**	-	aminotransferase	1.702	2.115	2.545	0.000
**SMU_378**	-	hypothetical protein	5.637	5.487	6.161	0.001
**SMU_379**	-	hypothetical protein	0.882	0.508	0.428	0.000
**SMU_381c**	-	transcriptional regulator	0.894	1.198	1.289	0.001
**SMU_382c**	-	conserved hypothetical protein (possible oxidoreductase)	0.964	1.345	1.052	0.000
**SMU_383c**	-	undetermined reductase or epimerase	2.232	2.251	1.998	2.212
**SMU_384**	-	hypothetical protein (possible transcriptional regulator)	0.956	0.834	0.655	0.001

**SMU_385**	-	probable glycoprotein endopeptidase	0.091	0.000	0.000	0.000
**SMU_386**	*rimI*	ribosomal-protein-alanine transferase	0.785	0.767	0.696	0.001
**SMU_387**	*gcp*	O-sialoglycoprotein endopeptidase	0.275	0.025	0.073	0.001
**SMU_388**	-	possible branched-chain amino acid permease	1.010	0.985	1.034	0.002
**SMU_389**	-	conserved hypothetical protein	0.523	0.677	0.413	0.000
**SMU_38c**	-	AcrR/TetR related transcriptional regulator	3.347	3.428	4.309	0.001
**SMU_39**	-	conserved hypothetical protein	5.413	6.226	8.472	0.002
**SMU_390**	-	hypothetical protein	0.241	0.704	1.026	0.000
**SMU_391c**	-	conserved hypothetical protein	1.937	1.238	1.126	0.001
**SMU_392c**	-	conserved hypothetical protein, HI0933-like	1.160	1.155	1.409	0.001
**SMU_393**	-	conserved hypothetical protein	0.006	0.000	0.000	0.000
**SMU_394c**	-	conserved hypothetical protein	1.104	1.271	1.420	0.000
**SMU_395**	*pepX*	X-prolyl dipeptidyl-peptidase	1.641	1.695	1.805	0.471
**SMU_396**	*glpF*	glycerol uptake facilitator protein	1.001	0.000	0.001	0.051
**SMU_399**	*cppA*	C3-degrading proteinase	1.635	0.605	0.383	0.001
**SMU_40**	-	conserved hypothetical protein	2.527	3.449	4.524	0.000
**SMU_400**	-	beta-lactamase family protein	2.462	2.683	2.548	0.096
**SMU_401c**	-	acetyltransferase	0.688	0.625	0.711	0.074
**SMU_402**	*pfl*	pyruvate formate-lyase	2.044	1.764	1.396	0.000
**SMU_403**	*dinP*	nucleotidyltransferase/DNA polymerase involved in DNA repair	1.001	0.934	1.061	0.000
**SMU_404c**	-	hypothetical protein	1.857	2.052	1.875	0.001
**SMU_405c**	-	transcriptional regulator	1.122	1.344	1.511	0.004
**SMU_406c**	-	haloacid dehalogenase-like hydrolase	0.925	0.924	1.618	0.000
**SMU_407**	-	conserved hypothetical protein	2.246	2.527	2.232	0.016
**SMU_408**	-	xanthine/uracil permease family protein	0.997	1.269	0.495	0.008
**SMU_409**	-	conserved hypothetical protein	0.114	0.002	0.004	0.060
**SMU_41**	-	hypothetical protein (protein)	3.789	3.643	3.544	0.000
**SMU_410**	*brpA*	transcriptional regulator	2.523	10.075	0.145	0.001
**SMU_411c**	-	Streptococcus-specific protein	0.580	1.205	0.637	0.000
**SMU_412c**	-	Hit-like protein	0.866	0.955	0.656	0.000
**SMU_413**	-	ABC transporter, ATP-binding protein	0.488	0.281	0.130	0.001
**SMU_414**	-	ABC transporter permease	0.751	0.520	0.259	0.001
**SMU_415**	-	conserved hypothetical protein, phosphotransferase enzyme family	0.000	0.000	0.000	0.001
**SMU_416**	*trmB*	tRNA (guanine-N(7)-)-methyltransferase	0.539	0.262	0.381	0.000
**SMU_417**	-	conserved hypothetical protein	0.719	0.049	0.006	0.001
**SMU_418**	*nusA*	transcription termination-antitermination factor NusA; elongation factor	0.000	0.001	0.001	0.001
**SMU_419**	-	conserved hypothetical protein	0.001	0.001	0.000	0.000
**SMU_42**	-	conserved hypothetical protein	2.692	4.061	4.137	0.016

**SMU_420**	-	ribosomal protein L7A family	0.112	0.002	0.000	0.000
**SMU_421**	*infB*	translation initiation factor IF-2	0.000	0.000	0.000	0.000
**SMU_422**	*rbfA*	ribosome binding factor A	0.174	0.031	0.029	0.000
**SMU_423**	*nlmD*	possible bacteriocin	4.506	2.959	2.820	0.001
**SMU_424**	*copY*	negative transcriptional regulator, CopY	2.348	1.314	1.268	0.000
**SMU_426**	*copA*	copper-transporting ATPase	1.314	1.177	1.126	0.001
**SMU_427**	*copZ*	copper chaperone	0.721	0.973	0.772	0.000
**SMU_428**	-	conserved hypothetical protein, Cof family	1.902	2.289	2.092	0.045
**SMU_429c**	-	hypothetical protein	2.389	3.094	2.463	0.484
**SMU_43**	-	conserved hypothetical protein (possible site-specific DNA-methyltransferase/restriction modification enzyme)	5.292	6.090	7.835	9.011
**SMU_431**	*cylA*	ABC transporter, ATP-binding protein (hemolysin/cytolysin)	1.466	2.047	2.167	0.001
**SMU_432**	*cylB*	ABC transporter, permease and solute binding protein	2.483	2.617	3.489	1.520
**SMU_433**	-	transcriptional regulator	1.895	1.861	1.870	0.000
**SMU_434**	-	conserved hypothetical protein	0.783	0.014	0.015	0.000
**SMU_435**	*nagA*	N-acetylglucosamine-6-phosphate deacetylase	2.445	2.454	2.088	4.877
**SMU_438c**	-	(R)-2-hydroxyglutaryl-CoA dehydratase activator-related protein	1.169	1.162	1.119	0.000
**SMU_439**	-	transcriptional regulator	1.481	1.071	1.284	0.001
**SMU_44**	-	conserved hypothetical protein	3.319	3.721	4.877	0.001
**SMU_440**	-	hypothetical protein	1.236	1.105	1.268	0.002
**SMU_441**	-	transcriptional regulator	0.453	0.348	0.394	0.001
**SMU_442**	-	conserved hypothetical protein	1.653	1.561	1.456	0.000
**SMU_444**	-	hypothetical protein	0.929	0.387	0.180	0.001
**SMU_445**	*sygA*	glycyl-tRNA synthetase alpha subunit	0.000	0.001	0.000	0.000
**SMU_446**	*sygB*	glycyl tRNA synthetase beta subunit	0.000	0.000	0.000	0.001
**SMU_447**	-	conserved hypothetical protein	0.231	0.061	0.155	0.000
**SMU_448**	-	conserved hypothetical protein	3.436	3.213	3.513	0.349
**SMU_449**	*proB*	gamma-glutamyl kinase	1.422	0.654	2.079	0.001
**SMU_45**	-	hypothetical protein	3.895	4.330	5.367	0.002
**SMU_450**	*proA*	gamma-glutamyl phosphate reductase	3.174	0.541	2.676	0.001
**SMU_451**	-	hypothetical protein	0.908	1.284	1.686	0.000
**SMU_453**	*mraW*	S-adenosyl-methyltransferase, MraW family	0.682	0.297	0.389	5.216
**SMU_454**	*ftsL*	cell division protein	0.404	0.225	0.002	0.030
**SMU_455**	*pbp2x*	penicillin-binding protein 2X	0.001	0.001	0.000	0.001
**SMU_456**	*mraY*	phospho-N-acetylmuramoyl-pentapeptide-transferase	0.001	0.001	0.000	0.002
**SMU_457**	-	hypothetical protein	0.734	1.262	1.192	0.000
**SMU_458**	*rheB*	ATP-dependent RNA helicase; DEAD/DEAH family	0.036	0.055	0.156	0.000
**SMU_459**	-	amino acid ABC transporter, substrate-binding protein	0.868	0.454	0.421	0.001

**SMU_46**	-	hypothetical protein	19.291	24.492	33.517	16.355
**SMU_460**	-	amino acid ABC transporter, permease	1.219	0.389	0.540	0.000
**SMU_461**	-	amino acid ABC transporter, ATP-binding protein	0.601	0.218	0.296	0.000
**SMU_462**	-	Streptococcus-specific protein	2.576	1.551	0.481	0.001
**SMU_463**	*trxB*	thioredoxin reductase (NADPH)	0.017	0.000	0.001	0.004
**SMU_464**	*pncB*	nicotinate phosphoribosyltransferase	0.107	0.004	0.067	0.006
**SMU_465**	*nadE*	NAD(+) synthetase (nitrogen-regulatory protein)	0.002	0.001	0.001	0.000
**SMU_466**	*pepC*	cysteine aminopeptidase C	0.843	0.619	0.736	0.071
**SMU_467**	*pbp1a*	penicillin-binding protein 1a	0.064	0.010	0.011	0.001
**SMU_469**	*recU*	recombination protein U	0.611	0.021	0.014	0.034
**SMU_47**	-	hypothetical protein	12.828	16.566	23.924	1.113
**SMU_470**	-	conserved hypothetical protein	2.303	6.375	1.826	0.001
**SMU_471**	-	conserved hypothetical protein	0.001	0.000	0.001	0.000
**SMU_472**	-	conserved hypothetical protein, RNA methylase family	1.526	3.010	1.236	0.000
**SMU_473**	-	conserved hypothetical protein	1.666	1.776	1.173	0.090
**SMU_474**	*luxS*	S-ribosylhomocysteine lyase (S-ribosylhomocysteinase)	1.484	2.706	1.912	0.001
**SMU_475**	-	conserved hypothetical protein	1.018	0.006	0.000	0.000
**SMU_478**	*kguA*	guanylate kinase	0.001	0.001	0.001	0.123
**SMU_479**	*rpoZ*	DNA-directed RNA polymerase, omega subunit	0.373	0.143	0.122	0.003
**SMU_48**	*purD*	phosphoribosylamine-glycine ligase	1.430	0.907	0.868	0.009
**SMU_480**	*priA*	primosomal replication factor Y (primosomal protein N')	0.368	0.003	0.002	0.010
**SMU_481**	*fmt*	methionyl-tRNA formyltransferase	0.001	0.000	0.003	0.003
**SMU_482**	*sunL*	RNA-binding Sun protein; possible rRNA methylase	1.481	1.126	1.059	0.022
**SMU_483**	*pppL*	phosphoprotein phosphatase (pppL protein)	0.624	0.003	0.000	0.001
**SMU_484**	*pknB*	serine/threonine protein kinase	1.493	0.046	0.036	0.002
**SMU_485**	*liaF*	conserved hypothetical protein	3.073	1.523	3.772	0.220
**SMU_486**	*liaS*	two-component sensor histidine kinase	2.228	0.745	4.123	0.000
**SMU_487**	*liaR*	two-component response regulator	0.968	0.841	3.107	0.001
**SMU_488**	*ppiB*	hydrolase; possible peptidyl-prolyl cis-trans isomerase	2.792	1.175	2.766	0.003
**SMU_489**	*pnpB*	polyribonucleotide nucleotidyltransferase (general stress protein 13)	1.627	0.371	0.425	0.000
**SMU_49**	-	hypothetical protein	1.127	0.938	0.629	0.001
**SMU_490**	*pflC*	pyruvate formate-lyase activating enzyme	1.418	1.576	1.390	0.001
**SMU_491**	-	transcriptional regulator, DeoR family	2.055	1.808	2.779	0.001
**SMU_493**	*pfl2*	formate acetyltransferase (pyruvate-formate lyase)	2.539	2.237	2.312	0.000

**SMU_494**	*mipB*	transaldolase family protein	0.756	0.542	0.397	0.001
**SMU_495**	*gldA*	glycerol dehydrogenase	1.386	1.681	1.751	0.000
**SMU_496**	*cysK*	cysteine synthetase A	1.230	0.854	0.979	0.000
**SMU_497c**	-	conserved hypothetical protein	2.390	2.167	1.796	0.000
**SMU_498**	*comF A*	late competence protein F	0.927	0.799	0.726	0.001
**SMU_499**	*comF C*	late competence protein required for DNA uptake	3.801	3.261	3.309	0.001
**SMU_50**	*purE*	phosphoribosylaminoimidazole carboxylase catalytic subunit	0.885	0.693	0.472	0.000
**SMU_500**	*yfiA*	ribosome-associated protein	4.307	9.055	4.912	0.719
**SMU_501**	-	hypothetical protein	1.986	2.099	1.893	0.129
**SMU_502**	-	conserved hypothetical protein	2.602	2.677	2.638	0.001
**SMU_503c**	-	hypothetical protein	1.578	1.171	1.374	0.990
**SMU_504**	*dam*	site-specific DNA-adenine methyltransferase	2.397	1.528	1.619	0.004
**SMU_505**	*dpn*	adenine-specific DNA methylase (DpnIIB)	3.236	3.362	3.802	0.001
**SMU_506**	*ssuRB*	type II restriction endonuclease	1.015	1.222	1.346	0.007
**SMU_507**	*glcR*	transcriptional regulator, DeoR family	0.595	0.730	0.814	0.008
**SMU_508**	-	conserved hypothetical protein (possible hydrolase)	0.731	0.633	0.592	0.013
**SMU_509**	-	conserved hypothetical protein	1.346	1.377	1.371	0.021
**SMU_51**	*purK*	phosphoribosylaminoimidazole carboxylase, ATPase subunit	1.104	0.836	0.485	0.001
**SMU_510c**	-	deoxyribonuclease	1.263	1.514	1.358	0.241
**SMU_512c**	-	conserved hypothetical protein	3.842	1.967	2.041	0.001
**SMU_513**	-	hypothetical protein	4.316	6.151	5.283	0.000
**SMU_514**	-	transcriptional regulator, AcrR family	1.193	1.398	1.163	0.002
**SMU_515**	*mycA*	67 kDa myosin-crossreactive antigen	1.457	1.541	1.479	0.004
**SMU_516**	-	conserved hypothetical protein, N6 adenine-specific DNA methylase signature domain	0.821	0.300	0.236	0.038
**SMU_517**	*coaD*	phosphopantetheine adenylyltransferase; lipopolysaccharide core biosynthesis protein	0.000	0.000	0.002	0.002
**SMU_518**	-	conserved hypothetical protein	1.945	1.863	1.476	0.002
**SMU_52**	-	conserved hypothetical protein	2.297	1.571	1.898	0.001
**SMU_520**	-	phosphodiesterase-related protein	1.462	1.405	1.044	0.001
**SMU_521**	-	conserved hypothetical protein	1.690	1.920	1.595	0.016
**SMU_522**	-	conserved hypothetical protein	1.481	2.256	1.891	0.000
**SMU_523**	-	conserved hypothetical protein, VanZ-like family	3.535	3.874	3.344	0.008
**SMU_524**	-	ABC transporter ATP-binding / permease protein	2.112	2.477	1.756	0.032
**SMU_525**	-	ABC transporter ATP-binding / permease protein	2.280	2.693	2.606	0.002
**SMU_526c**	-	transcriptional regulator	3.436	4.035	4.038	0.097
**SMU_527**	-	conserved hypothetical protein	1.601	1.700	1.773	0.000

**SMU_528c**	-	conserved hypothetical protein	1.828	1.704	0.993	0.135
**SMU_529**	-	hypothetical protein	1.995	1.061	0.759	0.032
**SMU_53**	-	conserved hypothetical protein	1.510	2.369	2.270	0.001
**SMU_530c**	-	conserved hypothetical protein	1.965	2.353	2.577	1.303
**SMU_531**	*pheA*	chorismate mutase; possible prephenate dehydrogenase	0.708	0.886	0.716	0.000
**SMU_532**	*trpE*	anthranilate synthase, component I	0.844	0.970	0.818	0.001
**SMU_533**	*trpG*	anthranilate synthase, component II	1.004	0.721	0.797	0.334
**SMU_534**	*trpD*	phosphoribosyl anthranilate transferase	1.172	1.237	0.992	0.000
**SMU_535**	*trpC*	indole-3-glycerol phosphate synthase	1.102	1.165	1.219	0.001
**SMU_536**	*trpF*	phosphoribosylanthranilate isomerase	1.274	1.162	0.945	0.000
**SMU_537**	*trpB*	tryptophan synthase, beta subunit	0.714	0.770	0.630	0.105
**SMU_538**	*trpA*	tryptophan synthase, alpha subunit	1.041	1.209	1.108	0.001
**SMU_539c**	*comC*	prepilin peptidase type IV	1.914	2.131	4.187	0.001
**SMU_54**	-	amino acid racemase	0.983	0.949	0.912	0.000
**SMU_540**	*dpr*	peroxide resistance protein / iron binding protein	3.169	0.470	0.143	0.000
**SMU_541**	-	conserved hypothetical protein	4.747	2.691	2.404	0.000
**SMU_542**	*glk*	glucose kinase	0.885	0.460	0.713	0.008
**SMU_543**	-	conserved hypothetical protein	1.893	1.570	1.688	0.002
**SMU_545**	-	hypothetical protein	1.472	1.831	2.226	0.540
**SMU_546**	*bipA*	GTP-binding protein (tyrosine phosphorylated protein A); possible elongation factor	0.951	0.281	0.171	0.000
**SMU_547**	-	conserved hypothetical protein	0.942	2.095	0.197	0.000
**SMU_548**	*murD*	D-glutamic acid adding enzyme MurD; UDP-N-acetylmuramoylalanine--D-glutamate ligase	0.001	0.000	0.001	1.103
**SMU_549**	*murG*	undecaprenyl-PP-MurNAc-pentapeptide-UDPGlcNAc GlcNAc transferase	0.023	0.007	0.016	0.002
**SMU_55**	-	hypothetical protein	5.695	4.958	5.816	0.001
**SMU_550**	*divIB*	cell division protein	1.026	1.368	0.093	0.008
**SMU_551**	*ftsA*	cell division protein FtsA	0.000	0.000	0.000	0.001
**SMU_552**	*ftsZ*	cell division protein FtsZ	3.707	0.000	0.000	0.002
**SMU_553**	*ylmE*	conserved hypothetical protein	0.174	0.130	0.112	0.003
**SMU_554**	*ylmF*	conserved hypothetical protein	1.256	0.256	0.250	0.000
**SMU_555**	*ylmG*	conserved hypothetical protein	0.925	0.812	0.552	0.004
**SMU_556**	*ylmH*	conserved hypothetical protein	0.241	0.277	0.169	0.001
**SMU_557**	*divIVA*	cell division initiation protein	0.245	0.226	0.039	0.000
**SMU_558**	*ileS*	isoleucine-tRNA synthetase	0.008	0.001	0.003	0.003
**SMU_56**	-	Streptococcus-specific protein	1.842	2.482	3.065	0.001
**SMU_560c**	-	conserved hypothetical protein	2.775	1.603	0.227	0.001
**SMU_561c**	*mutT*	mutator protein, Nudix hydrolase, MutT family	0.997	1.084	0.818	0.000
**SMU_562**	*clpE*	ATP-dependent protease ClpE	0.810	0.734	0.780	2.323

**SMU_563**	-	ornithine carbamoyltransferase	0.657	0.728	0.616	0.002
**SMU_564**	-	conserved hypothetical protein	0.331	0.245	0.182	0.000
**SMU_567**	*glnP*	amino acid (glutamine) ABC transporter permease	1.042	0.010	0.283	0.003
**SMU_568**	*glnQ*	glutamine ABC transporter, ATP-binding protein	0.936	0.027	0.428	0.007
**SMU_569**	*feoA*	ferrous ion transport protein A	0.927	1.222	1.308	0.000
**SMU_570**	*feoB*	ferrous ion transport protein B	0.926	1.035	0.870	0.377
**SMU_571**	-	hypothetical protein	1.350	1.353	1.001	0.001
**SMU_572**	*folD*	bifunctional protein: methylenetetrahydrofolate dehydrogenase / methenyltetrahydrofolate cyclohydrolase	1.390	0.048	0.000	0.008
**SMU_573**	-	conserved hypothetical protein	0.929	1.102	0.697	0.002
**SMU_574c**	*lrg*	effector of murein hydrolase	0.995	1.699	1.716	0.006
**SMU_575c**	*lrgA*	murein hydrolase regulator	1.744	2.404	2.221	0.047
**SMU_576**	*lytT*	response regulator	0.913	1.444	1.655	0.000
**SMU_577**	*lytS*	sensor histidine kinase	1.220	1.454	1.531	0.281
**SMU_58**	-	hypothetical protein	4.821	6.932	8.899	0.003
**SMU_580**	*xseA*	exodeoxyribonuclease VII, large subunit	0.639	0.223	0.292	0.000
**SMU_581**	*xseB*	exodeoxyribonuclease VII, small subunit	0.156	0.332	0.283	0.000
**SMU_582**	*fps*	farnesyl diphosphate synthase (geranyltranstransferase)	0.910	0.515	0.030	0.000
**SMU_583**	*hlyX*	probable hemolysin A	0.170	0.062	0.120	0.000
**SMU_584**	*argR*	arginine repressor	0.019	0.000	0.000	0.001
**SMU_585**	*recN*	DNA repair and genetic recombination protein	1.195	0.558	0.660	0.001
**SMU_586**	-	conserved hypothetical protein, DevG family	0.956	0.687	0.886	0.000
**SMU_587**	-	lipase/acylhydrolase with GDSL-like domain	1.182	0.858	1.017	0.003
**SMU_588**	-	conserved hypothetical protein	1.638	1.446	1.592	0.000
**SMU_589**	*hlpA*	histone-like DNA-binding protein	0.001	0.000	0.000	0.002
**SMU_59**	*purB*	adenylosuccinate lyase	0.362	0.000	0.163	0.000
**SMU_590c**	-	transposase fragment	0.081	0.048	0.030	0.000
**SMU_591c**	-	conserved hypothetical protein	4.014	5.529	4.337	0.002
**SMU_592c**	-	transcriptional regulator	2.700	4.453	2.701	0.004
**SMU_593**	*furR*	ferric uptake regulator protein FurR	2.351	2.998	1.649	0.002
**SMU_594**	-	hypothetical protein	2.172	3.148	1.654	0.001
**SMU_595**	*pyrD*	dihydroorotate dehydrogenase (dihydroorotate oxidase)	1.588	1.708	1.438	0.000
**SMU_596**	*pmgY*	phosphoglycerate mutase	0.000	0.000	0.000	0.000
**SMU_597**	*pbp2b*	penicillin-binding protein 2b	1.849	5.755	7.674	0.011
**SMU_598**	*recM*	recombination protein	0.653	0.059	0.042	0.000
**SMU_599**	*ddlA*	D-alanine-D-alanine ligase	0.029	0.042	0.049	0.005
**SMU_60**	*alkD*	DNA alkylation repair enzyme	2.203	1.061	2.134	0.000
**SMU_600c**	-	conserved hypothetical protein	1.905	1.747	1.486	0.001
**SMU_602**	-	sodium-dependent transporter	1.762	1.713	1.673	0.003

**SMU_603**	*murF*	D-Ala-D-Ala adding enzyme; UDP-N-acetylmuramoylalanyl-D-glutamyl-2,6-diaminopimelate-D-alanyl-D-alanyl ligase (UDP-N-acetylmuramyl pentapeptide synthase)	0.001	0.001	0.000	0.001
**SMU_604**	*yddK*	conserved hypothetical protein	3.923	4.291	1.850	0.012
**SMU_605**	-	hypothetical protein	3.032	6.228	2.556	0.002
**SMU_606**	-	hypothetical protein	2.337	4.971	3.125	0.164
**SMU_607**	-	conserved hypothetical protein	3.342	4.994	4.397	0.005
**SMU_608**	*prfC*	peptide chain release factor 3 ; translation elongation and release factor	1.369	0.668	1.061	0.020
**SMU_609**	*bsp*	cell wall protein precursor	0.507	0.509	0.565	0.001
**SMU_61**	*comR*	transcriptional regulator, Cro/CI family	2.456	2.809	4.336	380.442
**SMU_610**	*spaP*	cell surface antigen	0.885	1.082	1.139	0.326
**SMU_611**	*deaD*	ATP-dependent RNA helicase/DEAD family	0.407	0.029	0.117	0.004
**SMU_613**	-	hypothetical protein	1.902	1.050	1.570	0.001
**SMU_614**	-	hypothetical protein	0.650	0.482	0.904	0.002
**SMU_616**	-	hypothetical protein	1.771	3.128	4.427	0.000
**SMU_618**	-	hypothetical protein	0.985	0.152	0.211	0.002
**SMU_620**	-	hypothetical protein	1.415	1.412	1.929	0.001
**SMU_621c**	-	conserved hypothetical protein	1.565	1.285	1.894	0.012
**SMU_622c**	-	conserved hypothetical protein	0.828	1.168	1.208	0.000
**SMU_623c**	*yheN*	polysaccharide deacetylase	1.336	1.283	1.551	0.001
**SMU_624**	*plsC*	1-acylglycerol-3-phosphate O-acyltransferase	0.011	0.001	0.000	0.002
**SMU_625**	*comEA*	competence protein	1.120	0.913	1.678	0.001
**SMU_626**	*comEC*	competence protein; possible integral membrane protein	0.616	0.467	0.695	0.003
**SMU_627**	-	conserved hypothetical protein	0.551	0.934	1.306	0.077
**SMU_628**	*holA*	DNA polymerase III, delta subunit	0.001	0.001	0.000	0.003
**SMU_629**	*sodA*	superoxide dismutase	0.001	0.000	0.000	0.000
**SMU_630**	-	conserved hypothetical protein	2.342	2.509	3.770	0.001
**SMU_631**	-	conserved hypothetical protein	1.410	1.125	1.784	0.222
**SMU_632**	-	transcriptional regulator	0.504	0.720	0.613	0.000
**SMU_633**	*tesA*	thioesterase	1.294	1.226	1.486	0.002
**SMU_634**	*queA*	S-adenosylmethionine--tRNA ribosyltransferase-isomerase (queuosine (Q) biosynthesis protein)	0.763	0.665	0.797	1.112
**SMU_635**	-	conserved hypothetical protein	0.453	0.332	0.721	0.004
**SMU_636**	*nagB*	glucosamine-6-phosphate isomerase	0.685	0.725	0.865	0.000
**SMU_637c**	-	hypothetical protein	0.976	1.050	1.274	0.001
**SMU_638**	*rsuA*	ribosomal small subunit pseudouridine synthase A	0.907	0.819	1.232	0.001
**SMU_639**	*rimJ*	N-terminal acetyltransferase	2.018	2.732	2.948	0.000
**SMU_63c**	-	conserved hypothetical protein	1.687	2.085	2.125	0.239
**SMU_64**	*ruvB*	Holliday junction DNA helicase	0.412	0.013	0.013	0.001

**SMU_640c**	*ydeF*	transcriptional regulator	1.312	1.318	1.406	0.220
**SMU_641**	*qor*	oxidoreductase	1.512	1.297	1.062	0.160
**SMU_642**	-	hypothetical protein	1.658	2.300	2.944	0.000
**SMU_643**	*yeeB*	acetyl esterase/ sugar hydrolase	2.322	2.628	2.886	0.001
**SMU_644**	*coiA*	competence protein CoiA	0.926	1.041	1.228	0.002
**SMU_645**	*pepB*	PepB oligopeptidase	0.941	1.029	1.052	0.000
**SMU_646**	*gph*	hydrolase (possible phosphoglycolate phosphatase)	2.536	2.623	2.606	1.032
**SMU_647**	-	O-methyltransferase	0.707	0.603	3.507	0.745
**SMU_648**	*prtM*	protease maturation protein; possible peptidylprolyl isomerase	1.195	1.741	1.045	0.000
**SMU_649**	-	conserved hypothetical protein	0.932	0.891	1.209	0.000
**SMU_65**	*ptp*	protein tyrosine-phosphatase	1.381	1.232	0.601	0.000
**SMU_650**	*alaS*	alanyl-tRNA synthetase (alanine--tRNA ligase)	0.000	0.000	0.000	0.001
**SMU_651c**	-	ABC transporter, periplasmic substrate-binding protein	1.250	1.286	1.473	0.004
**SMU_652c**	*msmK*	ABC transporter, ATP-binding protein (possible nitrate transport system)	1.560	1.510	1.664	0.001
**SMU_653c**	*tauC*	ABC transporter, permease protein (possible taurine transport system permease)	0.717	0.739	0.774	0.022
**SMU_654**	*mutF*	ABC transporter, ATP-binding protein	1.075	1.199	1.649	0.000
**SMU_655**	*mutE1*	mutE like protein, possibly bacteriocin associated	1.037	1.390	1.483	0.000
**SMU_656**	*mutE2*	ABC transporter, permease, possibly bacteriocin associated	0.972	0.892	1.456	0.001
**SMU_657**	*mutG*	ABC transporter, permease, possibly bacteriocin associated	0.845	0.920	0.992	0.003
**SMU_658**	-	conserved hypothetical protein	1.185	1.470	1.476	0.021
**SMU_659**	*spaR*	response regulator	0.903	0.987	0.989	0.007
**SMU_66**	-	conserved hypothetical protein; possible phosphatidylinositol-4-phosphate 5-kinase	3.134	2.239	1.785	0.001
**SMU_660**	*spaK*	histidine kinase	0.636	0.906	0.946	0.001
**SMU_661**	-	transcriptional regulator	1.651	1.914	4.062	1.955
**SMU_662**	-	conserved hypothetical protein (possible membrane protein)	2.579	4.044	4.256	0.001
**SMU_663**	*argC*	N-acetyl-gamma-glutamyl-phosphate reductase (N-acetyl-glutamate-gamma-semialdehyde dehydrogenase)	1.817	1.745	1.581	0.010
**SMU_664**	*argJ*	ornithine acetyltransferase / N-acetylglutamate synthase	1.025	0.999	0.812	0.002
**SMU_665**	*argB*	acetylglutamate kinase	1.390	1.232	1.018	0.001
**SMU_666**	*argD*	N-acetylornithine aminotransferase	1.760	1.149	1.294	0.203
**SMU_667**	*nrdG*	ribonucleotide reductase, small subunit	0.000	0.000	0.000	0.001
**SMU_668c**	*nrdA*	ribonucleotide reductase, large subunit	0.000	0.000	0.000	0.002
**SMU_669c**	*nrdH*	glutaredoxin	0.601	0.100	0.162	0.001

**SMU_67**	-	acyltransferase	2.105	2.447	2.724	0.318
**SMU_670**	*citB*	aconitate hydratase; aconitase A	1.007	1.002	1.169	0.002
**SMU_671**	*citZ*	citrate synthase	1.492	1.269	1.418	0.000
**SMU_672**	*idh*	isocitrate dehydrogenase	0.788	0.826	0.955	0.000
**SMU_673**	-	conserved hypothetical protein	1.000	0.929	1.209	0.001
**SMU_674**	*ptsH*	phosphotransferase system phosphohistidine-containing protein	0.016	0.002	0.001	0.000
**SMU_675**	*ptsI*	phosphoenolpyruvate-protein phosphotransferase (enzyme I)	0.000	0.000	0.000	0.035
**SMU_676**	*gapN*	NADP-dependent non-phosphorylating glyceraldehyde-3-phosphate dehydrogenase	0.099	0.001	0.000	0.000
**SMU_677**	*rmeA*	transcriptional regulator, MerR family	1.210	0.775	0.386	0.032
**SMU_678**	*ycgG*	oxidoreductase, aldo/keto reductase family	2.458	1.969	1.637	0.070
**SMU_679**	*ycgG*	oxidoreductase, aldo/keto reductase family	1.562	1.845	1.008	0.001
**SMU_680**	-	carboxymuconolactone decarboxylase family protein	2.667	2.092	1.902	0.001
**SMU_681**	-	conserved hypothetical protein	2.220	2.040	2.707	0.000
**SMU_682**	-	hypothetical protein	2.959	3.031	3.414	0.102
**SMU_683**	-	conserved hypothetical protein	1.918	2.085	2.242	0.040
**SMU_684**	-	hypothetical protein	1.636	1.782	3.433	0.000
**SMU_685**	-	hypothetical protein	2.118	1.862	1.755	0.003
**SMU_687c**	-	hypothetical protein	1.184	1.549	1.623	0.000
**SMU_688**	-	conserved hypothetical protein	1.628	2.216	2.366	0.002
**SMU_689**	*atlA*	autolysin/endolysin	2.406	2.507	2.760	0.275
**SMU_690**	-	hypothetical protein	0.906	1.121	0.908	0.000
**SMU_691**	*pepT*	peptidase T (tripeptidase)	1.631	2.097	1.886	0.145
**SMU_692**	*thmA*	possible pore-forming peptide	1.084	0.221	0.152	0.002
**SMU_694c**	*fer*	ferredoxin	1.474	1.271	0.985	0.000
**SMU_695**	-	conserved hypothetical protein	2.104	2.052	1.298	0.001
**SMU_696**	*cmk*	cytidylate kinase	0.239	0.001	0.005	0.001
**SMU_697**	*infC*	translation initiation factor IF-3	0.000	0.000	0.000	0.000
**SMU_698**	*rpmI*	50S ribosomal protein L35	0.256	0.118	0.200	0.001
**SMU_699**	*rplT*	50S ribosomal protein L20	0.001	0.000	0.001	0.000
**SMU_70**	*thrC*	threonine synthase	0.006	0.002	0.000	0.521
**SMU_700c**	*gpmB*	phosphoglycerate mutase	1.515	2.351	1.868	0.000
**SMU_701c**	-	integral membrane protein	2.641	3.232	2.653	0.274
**SMU_702c**	*ydbF*	transcriptional regulator	0.886	3.402	2.205	0.000
**SMU_703c**	-	conserved hypothetical protein	1.443	1.357	1.399	0.001
**SMU_704c**	-	autolysin	1.749	1.682	1.907	0.000
**SMU_706c**	-	conserved hypothetical protein	0.764	0.492	0.557	0.003
**SMU_707c**	-	endolysin	0.846	0.746	1.604	0.001
**SMU_709**	-	conserved hypothetical protein	2.863	2.141	2.401	0.730
**SMU_71**	-	MATE efflux family protein	2.340	1.339	1.531	0.001
**SMU_711**	-	conserved hypothetical protein	1.391	1.007	1.126	0.001
**SMU_712**	*pepC*	phosphoenolpyruvate carboxylase	0.000	0.000	0.000	0.008
**SMU_713**	*ftsW*	cell division protein	0.002	0.001	0.002	0.002
**SMU_714**	*tuf*	translation elongation factor Tu	0.000	0.000	0.000	0.077
**SMU_715**	*tpiA*	triosephosphate isomerase	0.000	0.001	0.000	0.000

**SMU_716**	*murN*	peptidoglycan branched peptide synthesis protein, alanine adding enzyme; methicillin resistance factor	0.002	0.000	0.001	0.000
**SMU_717**	*murM*	beta-lactam resistance factor murM; Serine / alanine adding enzyme	0.043	0.426	0.021	0.002
**SMU_718c**	-	hydrolase, haloacid dehalogenase-like family	0.563	0.440	0.353	0.000
**SMU_719c**	-	conserved hypothetical protein	1.720	1.564	1.790	0.001
**SMU_72**	-	conserved hypothetical protein	2.245	4.467	2.203	0.003
**SMU_720**	-	conserved hypothetical protein	2.856	2.255	2.428	0.192
**SMU_721**	-	conserved hypothetical protein	0.612	0.574	0.536	0.001
**SMU_722**	-	hypothetical protein	1.045	1.712	2.473	0.001
**SMU_723**	*pacL*	calcium-transporting ATPase	0.140	0.000	0.039	0.027
**SMU_724**	*glpQ*	glycerophosphodiester phosphodiesterase	0.667	0.861	0.727	0.000
**SMU_725c**	-	conserved hypothetical protein	1.619	1.968	1.984	0.000
**SMU_727**	*merR*	mercury resistance operon negative regulator	1.519	1.661	1.731	0.000
**SMU_728**	-	oxidoreductase	1.723	1.938	1.855	0.010
**SMU_73**	-	conserved hypothetical protein	0.722	0.911	0.678	0.004
**SMU_730**	-	conserved hypothetical protein	1.638	1.787	1.197	0.000
**SMU_731**	-	ABC transporter, ATP-binding protein	1.681	2.074	2.169	0.852
**SMU_732**	-	conserved hypothetical protein	3.048	3.545	3.555	0.003
**SMU_734**	-	conserved hypothetical protein (probable membrane protein)	0.061	0.086	0.119	0.001
**SMU_735**	-	hypothetical protein	1.238	1.556	1.483	0.006
**SMU_737**	-	conserved hypothetical protein (possible alpha / beta superfamily hydrolase)	1.113	1.280	1.093	0.001
**SMU_738**	-	hypothetical protein	0.000	0.000	0.000	0.001
**SMU_739c**	-	hypothetical protein	1.348	1.330	1.281	0.000
**SMU_74**	*gmp*	phosphoglycerate mutase-like protein	2.335	2.951	3.148	0.001
**SMU_741**	-	conserved hypothetical protein	1.185	1.444	1.127	0.037
**SMU_742**	-	conserved hypothetical protein, Cof family	0.283	0.169	0.206	0.154
**SMU_743**	-	hydrolase, haloacid dehalogenase-like family	0.701	0.509	0.547	0.001
**SMU_744**	*ftsY*	cell division protein FtsY; signal recognition particle (docking protein)	0.173	0.029	0.014	0.000
**SMU_745**	*lmrB*	drug-export protein; multidrug resistance protein	1.095	1.251	1.726	0.002
**SMU_746c**	-	conserved hypothetical protein	1.044	1.497	0.024	0.001
**SMU_747c**	-	conserved hypothetical protein, permease-like	1.867	1.754	0.033	0.001
**SMU_748**	-	hypothetical protein	0.332	0.245	0.258	0.000
**SMU_75**	*dacF*	D-alanyl-D-alanine carboxypeptidase (penicillin binding protein)	2.487	4.396	4.812	0.017
**SMU_750c**	-		1.702	3.272	2.963	0.000

**SMU_751**	-	conserved hypothetical protein (possible transcriptional regulator)	0.666	0.247	0.293	0.001
**SMU_752**	-	conserved hypothetical protein	0.849	0.317	0.370	0.001
**SMU_753**	-	conserved hypothetical protein	2.232	0.920	0.271	0.001
**SMU_754**	*hprK*	HPr (serine) kinase/phosphatase	0.002	0.001	0.004	0.000
**SMU_755**	*lgt*	prolipoprotein diacylglyceryl transferase	0.438	0.099	0.005	0.001
**SMU_756**	-	conserved hypothetical protein	1.882	1.199	0.262	0.001
**SMU_757**	-	conserved hypothetical protein	1.158	1.454	0.472	0.002
**SMU_758c**	-	conserved hypothetical protein	1.624	2.696	0.839	7.118
**SMU_759**	-	protease, U32 family	1.524	1.572	1.503	0.000
**SMU_76**	*ampD*	N-acetyl-muramidase (N-acetylmuramoyl-L-alanine amidase)	2.849	5.876	6.250	0.001
**SMU_761**	*yugD*	protease; related to collagenase	1.289	1.020	0.999	0.000
**SMU_764**	*ahpC*	alkyl hydroperoxide reductase, subunit C	0.834	0.553	0.415	0.002
**SMU_765**	*ahpF*	alkyl hydroperoxide reductase, subunit F	0.564	0.432	0.354	0.001
**SMU_766**	-	transposase	0.000	0.000	0.000	0.000
**SMU_768c**	-	conserved hypothetical protein	1.193	1.147	1.391	0.006
**SMU_769**	-	conserved hypothetical protein	0.661	0.968	1.013	0.011
**SMU_770c**	-	manganese transporter/possible HitA ferric iron-binding periplasmic protein	1.125	0.713	1.042	7.557
**SMU_771c**	-	hypothetical protein	3.719	3.173	2.662	0.001
**SMU_772**	*gbpD*	bifunctional glucan-binding protein D and lipase	1.582	1.131	1.287	0.056
**SMU_773c**	*lysS*	lysyl-tRNA synthetase	0.000	0.001	0.000	0.000
**SMU_774**	-	hydrolase, haloacid dehalogenase-like	1.095	0.752	0.051	0.001
**SMU_775c**	-	conserved hypothetical protein	0.104	1.305	0.033	0.024
**SMU_776**	-	conserved hypothetical protein; possible methyltransferase	0.572	0.335	0.464	0.194
**SMU_777**	*aroD*	3-dehydroquinate dehydratase	0.000	0.001	0.000	0.001
**SMU_778**	*aroE*	shikimate 5-dehydrogenase	0.001	0.000	0.000	0.000
**SMU_779**	*aroB*	3-dehydroquinate synthase	0.000	0.000	0.001	0.001
**SMU_78**	*fruA*	fructan hydrolase; exo-beta-D-fructosidase	2.664	3.469	3.850	0.001
**SMU_780**	*aroC*	chorismate synthase	0.007	0.000	0.000	0.001
**SMU_781**	*tyrA*	prephenate dehydrogenase	0.048	0.019	0.025	0.001
**SMU_782**	-	conserved hypothetical protein	0.480	0.695	0.524	0.001
**SMU_784**	*aroA*	3-phosphoshikimate 1-carboxyvinyltransferase (5-enolpyruvylshikimate-3-phosphate synthase)	0.006	0.000	0.000	0.000
**SMU_785**	*aroK*	shikimate kinase	0.001	0.001	0.000	0.000
**SMU_786**	*pheA*	prephenate dehydratase	0.446	0.612	0.493	0.000
**SMU_787**	*psr*	transcriptional regulator, Psr-like protein	1.008	0.567	0.423	0.001
**SMU_788**	*trmA*	RNA methyltransferase, TrmA family	0.706	0.751	0.465	0.001

**SMU_789**	*blpY*	conserved hypothetical protein (similar to BlpY)	1.201	1.107	1.291	0.001
**SMU_79**	*fruB*	fructan hydrolase; exo-beta-D-fructosidase	2.622	3.240	3.502	0.002
**SMU_790**	-	conserved hypothetical protein	0.761	0.541	0.608	0.000
**SMU_791c**	-	hypothetical protein	1.511	1.673	1.943	0.008
**SMU_793**	-	conserved hypothetical protein	0.736	0.675	0.793	0.000
**SMU_794**	-	hypothetical protein	1.187	0.960	1.519	0.001
**SMU_795**	-	conserved hypothetical protein (possible esterase)	1.132	1.251	1.119	0.003
**SMU_796**	*ytfG*	conserved hypothetical protein (possible oxidoreductase)	1.605	2.014	1.790	0.162
**SMU_797**	-	conserved hypothetical protein	2.828	2.445	2.420	0.001
**SMU_798c**	-	hypothetical protein	0.288	0.475	0.457	0.000
**SMU_799c**	-	conserved hypothetical protein	0.801	0.745	0.608	0.002
**SMU_80**	*hrcA*	heat-inducible transcription repressor	0.251	0.005	0.001	0.001
**SMU_800**	-	conserved hypothetical protein	1.632	1.561	1.229	0.008
**SMU_801**	*obg*	GTP-binding protein, GTP1/Obg family	0.002	0.001	0.000	0.002
**SMU_802**	-	conserved hypothetical protein	0.179	0.177	0.610	0.000
**SMU_803c**	*ykhF*	ABC transporter, ATP-binding protein	0.432	0.400	0.397	0.001
**SMU_804**	-	hypothetical protein	5.120	6.487	6.821	0.120
**SMU_805c**	-	amino acid ABC transporter, ATP-binding protein	1.009	0.702	0.480	0.000
**SMU_806c**	*glnP*	glutamine ABC transporter, permease and solute binding protein	1.282	0.904	0.819	0.001
**SMU_807**	-	conserved hypothetical protein (possible membrane protein)	0.449	0.403	0.411	0.001
**SMU_809**	*uvrB*	excinuclease ABC (subunit B); helicase subunit of the DNA excision repair complex	1.467	0.737	0.783	0.001
**SMU_81**	*grpE*	co-chaperone protein GrpE	0.001	0.000	0.000	0.001
**SMU_811**	-	hypothetical protein	1.795	1.400	1.806	0.003
**SMU_812**	-	hypothetical protein	0.781	0.399	0.737	0.001
**SMU_813**	*yabB*	hypothetical protein (possible transcriptional regulator)	1.337	1.295	1.715	0.001
**SMU_814**	*mutT*	MutT/nudix family protein	0.854	0.787	1.030	0.003
**SMU_815**	-	amino acid ABC transporter, periplasmic amino acid-binding protein	1.115	1.434	1.288	0.000
**SMU_816**	*arcT*	aminotransferase	1.127	0.965	0.955	0.000
**SMU_817**	*glnH*	amino acid ABC transporter, substrate-binding protein	0.924	1.059	1.104	0.001
**SMU_818**	*rpsU*	30S ribosomal protein S21	0.066	0.006	0.009	0.000
**SMU_819**	*mscL*	large conductance mechanosensitive channel	1.767	1.320	1.294	0.000
**SMU_82**	*dnaK*	chaperone protein, DnaK	0.013	0.000	0.001	0.001
**SMU_820**	-	conserved hypothetical protein	1.277	1.205	1.227	0.001
**SMU_821**	*dnaG*	DNA primase	0.001	0.001	0.000	0.000
**SMU_822**	*rpoD*	RNA polymerase sigma-70 factor	0.000	0.000	0.000	0.000
**SMU_823**	-	conserved hypothetical protein	0.702	0.267	0.508	0.002

**SMU_824**	*rmlD*	dTDP-4-keto-L-rhamnose reductase	0.002	0.000	0.001	0.000
**SMU_825**	*rgpA*	polysaccharide biosynthesis protein; rhamnosyl transferase	0.111	0.000	0.000	0.034
**SMU_826**	*rgpB*	rhamnosyltransferase; polysaccharide biosynthesis protein	0.025	0.023	0.031	0.000
**SMU_827**	*rgpC*	polysaccharide ABC transporter membrane-spanning protein	0.002	0.001	0.001	0.001
**SMU_828**	*rgpD*	ABC transporter ATP-binding protein	0.083	0.063	0.018	0.001
**SMU_829**	*rgpE*	glycosyltransferase	0.010	0.001	0.002	0.001
**SMU_83**	*dnaJ*	co-chaperone protein DnaJ	0.148	0.008	0.000	0.000
**SMU_830**	*rgpF*	polysaccharide biosynthesis protein	0.001	0.001	0.001	0.001
**SMU_831**	-	conserved hypothetical protein	0.540	0.417	0.002	0.003
**SMU_832**	-	hypothetical protein	9.579	1.997	1.714	0.002
**SMU_833**	*gtrB*	glycosyltransferase, family 2	2.781	0.820	0.047	0.002
**SMU_834**	-	glycosyltransferase	2.624	1.127	3.391	0.001
**SMU_835**	-	conserved hypothetical protein (possible membrane protein)	10.925	9.396	3.594	0.090
**SMU_836**	-	hypothetical protein	2.041	2.896	1.863	0.000
**SMU_837**	*ycgG*	oxidoreductase, aldo/keto reductase family	1.416	1.457	1.076	0.000
**SMU_838**	*gshR*	glutathione reductase	1.423	1.177	1.305	0.001
**SMU_839**	*fol*	folyl-polyglutamate synthetase/ dihydrofolate synthase	0.941	0.807	0.682	0.004
**SMU_84**	*truA*	tRNA pseudouridine synthase A	0.916	0.521	0.717	0.001
**SMU_840c**	-	hypothetical protein	0.935	0.994	1.205	0.006
**SMU_841**	-	aminotransferase, class V; possible cysteine desulphurase	0.899	0.677	0.961	0.001
**SMU_842**	*thiI*	thiamine biosynthesis protein	0.709	0.562	0.788	0.000
**SMU_843**	-	; possible capsule biosynthesis protein CapA	1.214	0.856	1.069	0.160
**SMU_844**	-	conserved hypothetical protein	1.087	1.059	0.959	0.001
**SMU_845**	-	conserved hypothetical protein	1.886	1.536	2.304	0.096
**SMU_846**	*rplU*	Ribosomal protein L21-within SMU_847c on opposite strand	0.007	0.000	0.001	0.000
**SMU_847c**	-	hypothetical protein	0.041	0.020	0.011	0.002
**SMU_848**	-	conserved hypothetical protein	0.572	0.247	0.498	0.000
**SMU_849**	*rpmA*	50S ribosomal protein L27	0.000	0.000	0.000	0.000
**SMU_85**	*thiD*	phosphomethylpyrimidine kinase	2.242	1.443	0.539	0.000
**SMU_850**	-	acetyltransferase, GNAT family	1.200	1.161	1.248	0.000
**SMU_851**	-	conserved hypothetical protein	1.124	1.721	1.504	3.438
**SMU_852**	-	transcriptional regulator, LysR family	0.133	0.000	0.001	0.001
**SMU_853**	*lspA*	lipoprotein signal peptidase	0.260	0.066	0.034	0.001
**SMU_854**	*rluD*	ribosomal large subunit pseudouridine synthase D	0.919	0.158	0.479	0.000
**SMU_855**	-	conserved hypothetical protein	1.072	0.369	0.021	0.005
**SMU_856**	*pyrR*	bifunctional protein: pyrimidine operon regulatory protein and uracil phosphoribosyltransferase	0.602	0.465	0.025	0.036
**SMU_857**	*pbuX*	xanthine/uracil permease	0.866	0.870	0.096	0.002

**SMU_858**	*pyrB*	aspartate transcarbamoylase	0.768	0.619	0.008	0.001
**SMU_859**	*carA*	carbamoyl-phosphate synthase,small subunit	1.069	0.951	0.074	0.006
**SMU_86**	-	conserved hypothetical protein	2.253	1.796	1.932	0.001
**SMU_860**	*carB*	carbamoyl-phosphate synthase, large subunit	1.099	0.806	0.005	0.001
**SMU_862**	-	conserved hypothetical protein	1.247	1.455	1.145	6.750
**SMU_863**	-	ABC transporter, ATP-binding protein	0.507	0.892	0.476	0.007
**SMU_864**	-	ABC transporter, permease component	1.522	2.020	1.654	0.001
**SMU_865**	*rpsP*	30S ribosomal protein S16	0.019	0.000	0.000	0.000
**SMU_866**	-	conserved hypothetical protein	0.502	0.033	0.129	0.002
**SMU_867**	*rimM*	16S rRNA processing protein	0.165	0.041	0.008	0.000
**SMU_868**	*trmD*	tRNA (guanine-N1)-methyltransferase	0.002	0.001	0.000	0.020
**SMU_869**	*trxB2*	thioredoxin reductase	0.010	0.005	0.000	0.000
**SMU_87**	-	conserved hypothetical protein	1.767	1.585	1.722	0.000
**SMU_870**	*fruR*	lactose phosphotransferase system repressor/transcriptional repressor of the fructose operon	1.441	1.079	8.044	1.140
**SMU_871**	*fruB*	fructose-1-phosphate kinase	0.695	0.404	5.538	0.000
**SMU_872**	*fruA*	fructose-specific PTS system enzyme IIBC component	0.836	0.824	1.851	0.005
**SMU_873**	*metE*	5-methyltetrahydropteroyltriglutamate--homocysteine S-methyltransferase	0.701	0.877	0.935	0.001
**SMU_874**	*metH*	5-methyltetrahydrofolate--homocysteine methyltransferase	0.768	0.906	1.078	0.000
**SMU_876**	*msmR*	MSM operon regulatory protein	0.829	1.022	1.083	0.000
**SMU_877**	*agaL*	alpha-galactosidase (melibiase)	1.624	1.125	1.439	0.281
**SMU_878**	*msmE*	ABC transporter, sugar-binding protein	1.165	1.102	1.430	0.001
**SMU_879**	*msmF*	ABC transporter, sugar permease protein	1.531	1.390	1.541	0.010
**SMU_880**	*msmG*	multiple sugar-binding transport system permease protein MsmG	1.521	1.387	1.809	0.215
**SMU_881**	*gtfA*	sucrose phosphorylase	1.164	1.330	1.461	0.001
**SMU_882**	*msmK*	multiple sugar-binding transport ATP-binding protein MsmK	0.684	0.634	0.617	0.000
**SMU_883**	*dexB*	glucan 1,6-alpha-glucosidase	1.047	0.991	0.985	0.000
**SMU_885**	*galR*	galactose repressor	1.089	1.058	1.126	0.100
**SMU_886**	*galK*	galactokinase	0.986	0.913	0.816	1.489
**SMU_887**	*galT*	galactose-1-phosphate uridylyltransferase	1.464	1.612	1.576	0.690
**SMU_888**	*galE*	UDP-glucose 4-epimerase	0.615	0.793	0.823	0.000
**SMU_889**	*pbpX*	penicillin-binding protein 2X	0.779	0.647	0.793	0.003
**SMU_88c**	-	mechanosensitive ion channel protein	2.146	2.572	3.090	0.001
**SMU_890**	-	conserved hypothetical protein	2.872	11.425	0.751	0.349
**SMU_891**	*hsdM*	type I restriction-modification system DNA methylase	1.023	1.464	1.202	0.001

**SMU_892**	*hsdS*	type I restriction-modification system specificity determinant	1.389	1.383	1.290	0.019
**SMU_893**	*prrC*	anticodon nuclease	2.755	2.468	2.610	0.001
**SMU_895**	-	conserved hypothetical protein	0.092	0.008	0.000	0.002
**SMU_896**	-	conserved hypothetical protein	2.076	2.514	2.013	0.000
**SMU_897**	*hsdR*	type I restriction-modification system,helicase subunits	1.892	2.164	2.636	0.367
**SMU_898**	-	conserved hypothetical protein	0.480	0.436	0.155	0.002
**SMU_899**	-	conserved hypothetical protein, DegV family	1.508	1.302	1.243	0.000
**SMU_89c**	-	nitrite transporter	1.421	1.428	1.502	0.001
**SMU_900**	*dapB*	dihydrodipicolinate reductase	0.147	0.026	0.000	0.000
**SMU_901**	*cca*	CCA-adding enzyme; tRNA nucleotidyltransferase	0.019	0.014	0.001	0.000
**SMU_902**	*ypjG*	ABC transporter, ATP-binding protein	0.343	0.293	0.242	0.031
**SMU_905**	-	ABC-type multidrug/protein/lipid transport system, ATPase component	2.068	1.756	1.546	0.229
**SMU_906**	-	ABC transporter, ATP-binding / permease protein	1.195	0.907	0.829	0.001
**SMU_909**	*mln*	malate permease	1.047	1.078	1.062	0.000
**SMU_91**	*ropA*	peptidyl-prolyl isomerase, trigger factor	1.846	1.351	0.940	1.862
**SMU_910**	*gtfD*	glucosyltransferase-S	1.100	1.036	1.051	0.008
**SMU_911c**	-	hypothetical protein	0.835	0.714	1.208	0.001
**SMU_913**	-	NADP-specific glutamate dehydrogenase	1.243	1.059	0.469	0.001
**SMU_914c**	-	conserved hypothetical protein	1.917	2.106	2.184	0.000
**SMU_915c**	-	conserved hypothetical protein	1.342	1.240	1.218	0.826
**SMU_916c**	-	conserved hypothetical protein	1.986	2.097	1.767	15.802
**SMU_917c**	*ygcM*	6-pyruvoyl tetrahydropterin synthase, PTPS	1.311	1.320	1.011	0.770
**SMU_919c**	-	ATPase, confers aluminum resistance	3.155	2.309	1.750	30.804
**SMU_921**	*rcrR*	transcriptional regulator	1.233	0.639	0.458	0.001
**SMU_922**	*rcrP*	ABC-type multidrug / protein/ lipid transport system, ATPase component	0.139	0.132	0.229	0.001
**SMU_923**	*rcrQ*	ABC-type multidrug/protein/lipid transport system,ATPase component	0.092	0.047	0.066	0.001
**SMU_924**	*tpx*	thiol peroxidase	0.789	0.815	0.826	0.000
**SMU_925**	*cipI*	bacteriocin immunity protein	0.638	0.331	0.663	0.002
**SMU_926**	*relP*	GTP pyrophosphokinase family protein	0.938	1.013	1.128	0.001
**SMU_927**	*relR*	response regulator	1.097	0.787	0.687	0.002
**SMU_928**	*relS*	sensor histidine kinase	0.629	0.587	0.735	0.046
**SMU_929c**	-	conserved hypothetical protein	1.283	2.173	1.975	0.000
**SMU_92c**	-		1.104	1.228	1.271	0.000
**SMU_930c**	*cpsY*	transcriptional regulator	0.767	1.150	0.909	0.000
**SMU_932**	-	conserved hypothetical protein	0.837	0.645	0.499	0.000

**SMU_933**	*atmA*	amino acid ABC transporter, amino acid substrate-binding protein	1.338	1.347	1.022	54.969
**SMU_934**	-	amino acid ABC transporter, permease protein	1.748	2.031	1.609	0.001
**SMU_935**	-	amino acid ABC transporter, permease protein	0.902	0.962	0.969	0.002
**SMU_936**	-	amino acid ABC transporter, ATP-binding protein	0.391	0.425	0.455	0.000
**SMU_937**	*mvaD*	diphosphomevalonate decarboxylase	0.092	0.011	0.000	0.001
**SMU_938**	*mvaK2*	phosphomevalonate kinase	0.088	0.072	0.004	0.001
**SMU_939**	*fni*	isopentenyl-diphosphate delta-isomerase	0.002	0.003	0.001	0.005
**SMU_93c**	-		1.009	1.841	1.102	0.000
**SMU_940c**	*patB*	hemolysin III-related protein	2.515	0.003	0.009	0.003
**SMU_941c**	-	conserved hypothetical protein	0.311	0.052	0.050	0.005
**SMU_942**	*mvaA*	3-hydroxy-3-methylglutaryl-CoA reductase	0.001	0.000	0.005	0.001
**SMU_943c**	*hmcM*	hydroxymethylglutaryl-CoA synthase	0.001	0.000	0.000	0.000
**SMU_944**	*thyA*	thymidylate synthase	0.012	0.005	0.005	0.000
**SMU_946**	*pnuC*	transporter for NMN	1.611	1.508	1.460	0.002
**SMU_947**	*dfrA*	dihydrofolate reductase	0.000	0.000	0.000	0.000
**SMU_948**	-	conserved hypothetical protein	2.740	1.798	0.191	0.002
**SMU_949**	*clpX*	ATP-dependent Clp protease subunit X	0.972	0.028	0.011	0.000
**SMU_94c**	*tpn*	transposase fragment	1.681	2.101	1.909	0.009
**SMU_950**	*era*	GTP-binding protein	0.000	0.001	0.000	0.007
**SMU_951**	*hutM*	amino acid permease	0.495	0.509	0.347	0.001
**SMU_952**	*mmuM*	homocysteine S-methyltransferase	1.800	1.902	1.643	0.003
**SMU_953c**	-	transcriptional regulator, GntR family	1.448	1.791	1.372	0.133
**SMU_954**	*pdxK*	pyridoxal kinase	1.823	1.834	1.546	0.001
**SMU_955**	-	conserved hypothetical protein	0.377	0.507	0.384	0.001
**SMU_956**	*clp*	ATP-dependent Clp protease, ATP-binding subunit	0.788	0.725	0.634	0.001
**SMU_957**	*rplJ*	50S ribosomal protein L10	0.000	0.000	0.000	0.000
**SMU_958**	-	hypothetical protein	0.035	0.001	0.000	0.000
**SMU_959c**	-	hypothetical protein	0.000	0.000	0.002	0.000
**SMU_96**	*rpoE*	DNA-directed RNA polymerase, delta subunit	0.118	0.007	0.019	0.000
**SMU_960**	*rpl*	50S ribosomal protein L7/L12	0.000	0.000	0.000	0.000
**SMU_961**	-	macrophage infectivity potentiator-related protein	3.809	4.789	4.505	0.652
**SMU_962**	*mmgC*	acyl-CoA dehydrogenase	0.705	0.717	0.695	0.004
**SMU_963c**	-	polysaccharide deacetylase family protein	1.251	1.052	1.020	0.001
**SMU_965**	*hom*	homoserine dehydrogenase	0.000	0.000	0.000	0.000
**SMU_966**	*thrB*	homoserine kinase	0.000	0.001	0.000	0.023
**SMU_967**	*folC*	bifunctional protein: folylpolyglutamate synthase/dihydrofolate synthase	0.135	0.003	0.001	0.001

**SMU_968**	*folE*	GTP cyclohydrolase I	0.777	0.499	0.025	0.276
**SMU_969**	*folP*	dihydropteroate synthase	0.382	0.233	0.000	0.001
**SMU_97**	*pyrG*	CTP synthetase	0.000	0.000	0.000	0.000
**SMU_970**	*folA*	dihydroneopterin aldolase	0.998	1.133	0.002	0.000
**SMU_971**	*folK*	2-amino-4-hydroxy-6-hydroxymethyldihydropteridine pyrophosphokinase	1.940	1.418	0.001	0.699
**SMU_972**	*murB*	UDP-N-acetylenolpyruvoylglucosamine reductase	0.012	0.086	0.067	0.000
**SMU_973**	*potA*	ABC transporter ATP-binding protein - spermidine/putrescine transport	0.563	0.282	0.313	0.001
**SMU_974**	*potB*	spermidine / putrescine ABC transporter (permease protein)	0.885	0.258	0.373	0.001
**SMU_975**	*potC*	spermidine/putrescine ABC transporter, permease	1.204	0.530	0.721	0.004
**SMU_976**	*potD*	spermidine/putrescine ABC transporter, spermidine/putrescine-binding protein	2.101	1.489	2.800	0.002
**SMU_977**	*licT*	transcription antiterminator LicT (fragment)	0.790	0.988	0.761	0.000
**SMU_980**	*bglP*	beta-glucoside-specific EII permease	0.812	0.787	0.835	23.142
**SMU_981**	*bglB1*	beta-glucosidase, BglB protein	1.331	1.069	0.998	0.000
**SMU_982**	*bglB2*	beta-glucosidase, BglB protein	1.140	1.162	1.204	0.000
**SMU_983**	*bglC*	transcriptional regulator	1.171	0.861	0.794	0.009
**SMU_984**	-	hypothetical protein	2.246	3.092	2.541	0.002
**SMU_985**	*bglA*	6-phospho-beta-glucosidase	1.115	1.192	1.160	0.002
**SMU_986c**	-	conserved hypothetical protein	1.367	1.326	1.581	0.000
**SMU_987**	*wapA*	cell wall surface anchor family protein	1.257	1.593	1.437	0.202
**SMU_988**	*cls*	cardiolipin synthase	1.097	0.323	0.223	0.002
**SMU_989**	*asd*	aspartate-semialdehyde dehydrogenase	0.002	0.001	0.000	0.013
**SMU_99**	*fbaA*	fructose-bisphosphate aldolase	0.000	0.000	0.000	0.001
**SMU_990**	*dapA*	dihydrodipicolinate synthase	0.947	0.377	0.403	0.003
**SMU_991**	-	conserved hypothetical protein (similar to ribonucleotide reductase alpha subunit)	2.766	3.526	3.586	0.001
**SMU_992**	-	hypothetical protein	1.190	1.124	1.092	0.117
**SMU_993**	*ylqL*	GTP-binding protein	0.000	0.000	0.000	0.000
**SMU_994**	*rnh*	ribonuclease HII	0.282	0.143	0.202	0.000
**SMU_995**	*yclN*	ferrichrome ABC transporter (permease)	1.156	1.318	1.135	0.022
**SMU_996**	*yclN*	ABC transporter, permease protein;possible ferrichrome transport system	1.659	1.571	1.612	0.013
**SMU_997**	*fecE*	inorganic ion ABC transporter,ATP-binding protein; possible ferrichrome transport system	0.953	1.081	1.016	0.001

**SMU_998**	*fatB*	ABC transporter, ferrichrome-binding protein	0.442	0.613	0.366	0.001
**SMU_999**	-	hypothetical protein	1.569	1.311	1.283	0.002
**SMU_r01**	-		0.000	0.000	0.000	0.000
**SMU_r02**	-		0.000	0.000	0.000	0.000
**SMU_r03**	-		0.000	0.000	0.000	0.000
**SMU_r04**	-		0.000	0.000	0.000	0.000
**SMU_r05**	-		0.000	0.000	0.000	0.000
**SMU_r06**	-		0.000	0.000	0.000	0.000
