# Supplementary material for: Genomewide Identification of Essential Genes and Fitness Determinants of Streptococcus mutans UA159
Source: mSphere. 2018 Feb 7;3(1):e00031-18. doi: 10.1128/mSphere.00031-18 (PMC5806208; doi:10.1128/mSphere.00031-18)
Supplement: TABLE S3 [file sph001182474st3.pdf]

Table S3

Locus	Gene	Product	*S. agalactiae* A909	*S. pneumoniae*	*S. pyogenes* MGAS5448	*S. sanguninis*	Essential in other bacteria[a]	Core Genome[b]
**SMU_01**	*dnaA*	chromosomal replication initiator protein DnaA	essential	non	essential	essential	-	yes
**SMU_02**	*dnaN*	DNA polymerase III, beta subunit	essential	non	essential	essential	-	yes
**SMU_07**	*pth*	peptidyl-tRNA hydrolase	essential	essential	non	essential	-	yes
**SMU_1045c**	*ppnK*	NAD(+) kinase (ATP-NAD kinase)	essential	essential	essential	essential	-	no
**SMU_1051**	*nifS*	aminotransferase (class V); possible iron-sulfur cofactor synthesis protein;pyridoxal-phosphate dependent aminotransferase	non	essential	essential	essential	-	no
**SMU_1075**	*dfp*	DNA/pantothenate metabolism flavoprotein	non	essential	essential	non	-	no
**SMU_1085**	*rf1*	peptide chain release factor 1	essential	essential	essential	essential	-	yes
**SMU_1114**	*gyrA*	DNA gyrase A subunit	essential	non	essential	essential	-	yes
**SMU_1115**	*ldh*	L-lactate dehydrogenase	non	non	non	non	essential	yes
**SMU_1126**	*coaA*	pantothenate kinase	non	essential	essential	non	-	yes
**SMU_1136**	*pstC*	phosphate ABC transporter, permease	essential	non	non	essential	-	yes
**SMU_1137**	*pstC1*	phosphate ABC transporter, permease	essential	non	non	non	-	yes
**SMU_1138**	*pstS*	phosphate ABC transporter, periplasmic phosphate-binding protein	essential	non	non	essential	-	yes
**SMU_1143c**	*mreA*	bifunctional protein: riboflavin kinase/flavin adenine dinucleotide (FAD) synthase	essential	non	essential	essential	-	yes

**SMU_1187**	*glmS*	L-glutamine-D-fructose-6-phosphate amidotransferase	essential	essential	essential	essential	-	yes
**SMU_1190**	*pykF*	pyruvate kinase	essential	essential	essential	essential	-	yes
**SMU_1191**	*pfk*	6-phosphofructokinase	essential	essential	essential	essential	-	yes
**SMU_1192**	*dnaE*	DNA-polymerase III subunit alpha	essential	essential	essential	essential	-	yes
**SMU_1201c**	-	conserved hypothetical protein	non	non	non	non	non	yes
**SMU_1204**	*parC*	DNA topoisomerase IV subunit A	essential	essential	essential	essential	-	no
**SMU_1210**	*parE*	DNA topoisomerase IV subunit B	essential	essential	essential	essential	-	no
**SMU_1211**	-	conserved hypothetical protein	essential	essential	essential	essential	-	no
**SMU_123**	*polC*	DNA polymerase III, alpha subunit	essential	non	essential	essential	-	yes
**SMU_1247**	*eno*	enolase	essential	essential	essential	essential	-	no
**SMU_1277**	*gyrB*	DNA gyrase subunit B	essential	essential	essential	essential	-	no
**SMU_1294**	*flaW*	flavodoxin	essential	non	non	non	-	yes
**SMU_13**	-	cell-cycle protein, PP-loop superfamily	essential	non	essential	essential	-	yes
**SMU_1326**	*prfB*	peptide chain release factor	essential	essential	essential	essential	-	no
**SMU_1426c**	*femD*	phosphoglucosamine mutase (phosphoacetylglucosamine mutase)	essential	essential	essential	essential	-	yes
**SMU_1429**	*murC2*	UDP-N-acetylmuramyl tripeptide synthetase	essential	essential	essential	essential	-	yes
**SMU_1457**	*rmlB*	dTDP-glucose-4,6-dehydratase	essential	essential	essential	non	-	yes
**SMU_1460**	*rmlC*	dTDP-4-dehydrorhamnose 3,5-epimerase	essential	non	non	non	-	yes
**SMU_1461**	*rmlA*	glucose-1-phosphate thymidyltransferase	essential	non	essential	essential	-	yes

**SMU_1465c**	*dnaD*	DNA replication protein DnaD	essential	essential	non	essential	-	yes
**SMU_1474c**	*rnz*	ribonuclease Z	essential	essential	essential	essential	-	yes
**SMU_15**	*ftsH*	cell division protein FtsH	essential	non	essential	non	-	yes
**SMU_1510**	*syfB*	phenylalanyl-tRNA synthetase, beta subunit	essential	essential	essential	essential	-	yes
**SMU_1512**	*syfA*	phenylalanyl-tRNA synthetase, alpha subunit	essential	non	essential	essential	-	yes
**SMU_1528**	*atpB*	ATPase beta subunit	essential	essential	essential	essential	-	yes
**SMU_1529**	*atpC*	ATPase, gamma subunit	essential	essential	essential	essential	-	yes
**SMU_1530**	*atpD*	ATP synthase alpha subunit	essential	essential	essential	essential	-	yes
**SMU_1534**	*atpH*	ATPase, c subunit	essential	non	non	essential	-	yes
**SMU_1543**	*dnlJ*	DNA ligase	essential	essential	essential	essential	-	yes
**SMU_1573**	*metK*	S-adenosylmethionine synthetase	essential	essential	essential	essential	-	yes
**SMU_158**	*cysS*	cysteinyl-tRNA synthetase	essential	non	essential	essential	-	yes
**SMU_1581**	*dnaX*	DNA polymerase III subunits gamma and tau	essential	essential	essential	essential	-	yes
**SMU_1609c**	*secG*	preprotein translocase, SecG subunit	essential	essential	essential	essential	-	yes
**SMU_1613c**	-	dephospho-CoA kinase	essential	essential	essential	essential	-	yes
**SMU_1618**	*dagK*	diacylglycerol kinase	non	non	essential	non	-	yes
**SMU_1625**	*pyrH*	uridylate kinase (UMP-kinase)	essential	essential	essential	essential	-	yes
**SMU_1626**	*rl1*	50S ribosomal protein L1	essential	non	essential	essential	-	yes
**SMU_1627**	*rl11*	50S ribosomal protein L11	essential	non	non	essential	-	yes
**SMU_1663**	*kthY*	thymidylate kinase	essential	non	essential	essential	-	yes
**SMU_1687**	*ppaC*	manganese-dependent inorganic pyrophosphatase (type II PPase)	essential	essential	non	essential	-	yes
**SMU_169**	*rplM*	50S ribosomal protein L13	essential	non	non	essential	-	yes
**SMU_1734**	*accA*	acetyl-CoA carboxylase alpha subunit	essential	essential	essential	essential	-	yes

**SMU_1735**	*accD*	acetyl-CoA carboxylase beta subunit	essential	essential	essential	essential	-	yes
**SMU_1736**	*accC*	acetyl-CoA carboxylase biotin carboxylase subunit	essential	essential	essential	essential	-	yes
**SMU_1737**	*fabZ*	(3R)-hydroxymyristoyl-(acyl carrier protein) dehydratase	essential	essential	non	essential	-	yes
**SMU_1738**	*bccP*	biotin carboxyl carrier protein of acetyl-CoA carboxylase	essential	essential	essential	essential	-	yes
**SMU_1740**	*fabG*	3-oxoacyl-acyl-carrier-protein reductase / 3-ketoacyl-acyl carrier protein reductase	essential	essential	essential	essential	-	yes
**SMU_1742c**	*fabK*	trans-2-enoyl-ACP reductase II	essential	essential	non	essential	-	yes
**SMU_1743**	*acp*	acyl carrier protein	essential	non	essential	essential	-	yes
**SMU_1748**	*akh*	aspartate kinase / homoserine dehydrogenase	non	non	non	non	essential	yes
**SMU_1783**	*proS*	prolyl-tRNA synthetase	essential	essential	essential	essential	-	yes
**SMU_1785**	*cdsA*	phosphatidate cytidylyltransferase (CDP-diglyceride synthase)	essential	non	essential	essential	-	yes
**SMU_1799**	*nadD*	nicotinate mononucleotide adenylyltransferase (nicotinate-nucleotide adenylyltransferase)	essential	essential	essential	non	-	yes
**SMU_18**	-	hypothetical protein	non	non	non	non	non	no
**SMU_1801c**	-	conserved hypothetical protein, GTP-binding	non	essential	non	non	-	yes
**SMU_1802c**	-	conserved hypothetical protein	essential	essential	essential	essential	-	yes
**SMU_1819**	*gatB*	glutamyl-tRNA (Gln) amidotransferase subunit B	essential	essential	essential	essential	-	yes
**SMU_1820c**	*gatA*	glutamyl-tRNA(Gln) amidotransferase subunit A	essential	non	essential	essential	-	yes

**SMU_1821c**	*gatC*	glutamyl-tRNA (Gln) amidotransferase subunit C	essential	essential	essential	essential	-	yes
**SMU_1834**	*alr*	alanine racemase	essential	essential	non	essential	-	yes
**SMU_1836**	*aroG*	phospho-2-dehydro-3-deoxyheptonate aldolase (DAHP synthase)	non	non	non	non	essential	yes
**SMU_1837**	*aroH*	3-deoxy-7-phosphoheptulonate synthase	non	non	non	non	essential	no
**SMU_1852**	-	cation transporter, CorA family	essential	non	essential	non	-	yes
**SMU_1859**	*ssb*	single-stranded DNA-binding protein	essential	essential	non	essential	-	yes
**SMU_1860**	*rs6*	30S ribosomal protein S6	non	non	non	essential	-	yes
**SMU_1886**	*sys*	seryl-tRNA synthetase	non	essential	essential	essential	-	yes
**SMU_1920**	*pgdA*	phosphoglycerate dehydrogenase	essential	essential	essential	essential	-	yes
**SMU_1921**	*dnaI*	DNA replication protein; primosome component	essential	essential	essential	essential	-	yes
**SMU_1922**	*dnaB*	chromosome replication initiation and membrane attachment protein	essential	non	essential	essential	-	yes
**SMU_1943**	*syl*	leucyl-tRNA synthetase	essential	non	essential	essential	-	yes
**SMU_1948**	*secE*	preprotein translocase, SecE subunit	essential	non	non	essential	-	yes
**SMU_1954**	*groEL*	chaperonin GroEL	non	essential	essential	essential	-	yes
**SMU_2000**	*rl17*	50S ribosomal protein L17	essential	non	essential	essential	-	yes
**SMU_2001**	*rpoA*	DNA-directed RNA polymerase, alpha subunit	essential	non	essential	essential	-	yes
**SMU_2004**	*if1*	translation initiation factor IF-1	essential	non	essential	essential	-	yes

**SMU_2005**	*adk*	adenylate kinase (ATP-AMP transphosphorylase) (superoxide-inducible protein 16)	essential	essential	non	essential	-	yes
**SMU_2006**	*secY*	preprotein translocase	essential	non	essential	essential	-	yes
**SMU_2007**	*rl15*	50S ribosomal protein L15, N-terminal fragment	essential	non	non	non	-	yes
**SMU_2008**	*rl30*	50S ribosomal protein L30	essential	non	essential	non	-	yes
**SMU_2010**	*rl18*	50S ribosomal protein L18	non	non	non	essential	-	yes
**SMU_2012**	*rs8*	30S ribosomal protein S8	essential	non	non	essential	-	yes
**SMU_2014**	*rs14*	30S ribosomal protein S14	essential	non	non	essential	-	yes
**SMU_2015**	*rl5*	50S ribosomal protein L5	essential	non	non	non	-	yes
**SMU_2016**	*rl24*	50S ribosomal protein L24	essential	non	non	essential	-	yes
**SMU_2017**	*rl14*	50S ribosomal protein L14	essential	non	non	essential	-	yes
**SMU_2018**	*rs17*	30S ribosomal protein S17	essential	non	non	essential	-	yes
**SMU_2019**	*rl29*	50s ribosomal protein L29	essential	non	non	non	-	yes
**SMU_2020**	*rl16*	50S ribosomal protein L16	essential	non	essential	essential	-	yes
**SMU_2021**	*rs3*	30S ribosomal protein S3	essential	non	essential	essential	-	yes
**SMU_2022**	*rl22*	50S ribosomal protein L22	essential	non	essential	essential	-	yes
**SMU_2023c**	*rps19*	30S ribosomal protein S19, C-terminal fragment	essential	non	non	essential	-	yes
**SMU_2024c**	*rl4*	50S ribosomal protein L4, N-terminal fragment	essential	non	essential	essential	-	yes
**SMU_2025**	*rl3*	50S ribosomal protein L3	essential	non	essential	essential	-	yes
**SMU_2031**	*eftS*	translation elongation factor Ts	essential	non	essential	essential	-	yes
**SMU_2032**	*rs2*	30S ribosomal protein S2	non	non	essential	essential	-	yes
**SMU_2093**	*argR*	arginine repressor	non	non	non	non	essential	yes
**SMU_2102**	*hisS*	histidine-tRNA synthetase	essential	non	essential	essential	-	yes
**SMU_2135c**	*rpsD*	30S Ribosomal protein S4	essential	non	essential	essential	-	yes

**SMU_2143c**	*trmU*	tRNA (5-methylaminomethyl-2-thiouridylate)-methyltransferase	essential	non	essential	essential	-	yes
**SMU_2149c**	-	ABC transporter, ATP-binding protein	essential	essential	essential	essential	-	yes
**SMU_2151**	*pgsA*	CDP-diacylglycerol--glycerol-3-phosphate 3-phosphatidyltransferase	essential	essential	non	essential	-	yes
**SMU_2158c**	*trsA*	tryptophanyl-tRNA synthetase	essential	essential	essential	essential	-	yes
**SMU_2166**	*rplW*	Ribosomal protein L23	essential	non	non	essential	-	no
**SMU_22**	*gbpB*	glucan-binding protein B	essential	non	essential	non	-	yes
**SMU_23**	*prs*	phosphoribosyl pyrophosphate synthetase (PRPP synthetase)	essential	non	essential	essential	-	yes
**SMU_307**	*pgi*	glucose-6-phosphate isomerase	essential	essential	essential	essential	-	yes
**SMU_322c**	*galU*	glucose-1-phosphate uridylyltransferase	essential	essential	essential	essential	-	yes
**SMU_323**	*gpsA*	glycerol-3-phosphate dehydrogenase (NAD(P)+)	essential	non	essential	non	-	yes
**SMU_330**	*gltX*	glutamyl-tRNA synthetase	essential	essential	essential	essential	-	yes
**SMU_357**	*rpsL*	30S ribosomal protein S12	non	non	essential	essential	-	yes
**SMU_358**	*rpsG*	30S ribosomal protein S7	essential	non	essential	essential	-	yes
**SMU_359**	*efg*	translation elongation factor G/EF-2	essential	essential	essential	essential	-	yes
**SMU_360**	*gapC*	glyceraldehyde-3-phosphate dehydrogenase; plasmin receptor	essential	essential	essential	essential	-	yes
**SMU_361**	*pgk*	phosphoglycerate kinase	essential	essential	essential	essential	-	yes
**SMU_368c**	-	metallo-beta-lactamase superfamily protein	essential	essential	essential	essential	-	yes
**SMU_393**	-	conserved hypothetical protein	non	non	non	non	non	yes

**SMU_415**	-	conserved hypothetical protein, phosphotransferase enzyme family	essential	non	essential	essential	-	yes
**SMU_418**	*nusA*	transcription termination-antitermination factor NusA; elongation factor	non	essential	essential	non	-	yes
**SMU_419**	-	conserved hypothetical protein	essential	essential	non	non	-	yes
**SMU_421**	*infB*	translation initiation factor IF-2	essential	non	essential	non	-	yes
**SMU_445**	*sygA*	glycyl-tRNA synthetase alpha subunit	essential	essential	essential	essential	-	yes
**SMU_446**	*sygB*	glycyl tRNA synthetase beta subunit	essential	non	essential	essential	-	yes
**SMU_455**	*pbp2x*	penicillin-binding protein 2X	essential	essential	essential	essential	-	yes
**SMU_456**	*mraY*	phospho-N-acetylmuramoyl-pentapeptide-transferase	essential	essential	essential	essential	-	yes
**SMU_465**	*nadE*	NAD(+) synthetase (nitrogen-regulatory protein)	essential	non	essential	essential	-	yes
**SMU_471**	-	conserved hypothetical protein	non	non	non	non	essential	yes
**SMU_478**	*kguA*	guanylate kinase	essential	non	essential	essential	-	yes
**SMU_481**	*fmt*	methionyl-tRNA formyltransferase	essential	essential	essential	essential	-	yes
**SMU_517**	*coaD*	phosphopantetheine adenylyltransferase; lipopolysaccharide core biosynthesis protein	essential	essential	essential	essential	-	yes
**SMU_548**	*murD*	D-glutamic acid adding enzyme MurD; UDP-N-acetylmuramoylalanine--D-glutamate ligase	essential	essential	essential	essential	-	yes
**SMU_551**	*ftsA*	cell division protein FtsA	essential	essential	essential	non	-	yes

**SMU_558**	*ileS*	isoleucine-tRNA synthetase	essential	essential	essential	essential	-	yes
**SMU_589**	*hlpA*	histone-like DNA-binding protein	essential	essential	non	essential	-	yes
**SMU_596**	*pmgY*	phosphoglycerate mutase	essential	non	non	essential	-	yes
**SMU_603**	*murF*	D-Ala-D-Ala adding enzyme; UDP-N-acetylmuramoylalanyl-D-glutamyl-2,6-diaminopimelate-D-alanyl-D-alanyl ligase (UDP-N-acetylmuramyl pentapeptide synthase)	essential	essential	essential	essential	-	yes
**SMU_628**	*holA*	DNA polymerase III, delta subunit	essential	non	essential	essential	-	yes
**SMU_629**	*sod*	superoxide dismutase	non	non	non	non	essential	yes
**SMU_650**	*alaS*	alanyl-tRNA synthetase (alanine--tRNA ligase)	essential	non	essential	essential	-	yes
**SMU_667**	*nrdB*	ribonucleotide reductase, small subunit	essential	non	non	essential	-	yes
**SMU_668c**	*nrdA*	ribonucleotide reductase, large subunit	essential	essential	non	essential	-	yes
**SMU_675**	*ptsI*	phosphoenolpyruvate-protein phosphotransferase (enzyme I)	essential	non	non	non	-	yes
**SMU_697**	*infC*	translation initiation factor IF-3	essential	essential	non	essential	-	yes
**SMU_699**	*rplT*	50S ribosomal protein L20	essential	non	non	essential	-	yes
**SMU_70**	*thrC*	threonine synthase	non	non	non	non	essential	yes
**SMU_712**	capP	phosphoenolpyruvate carboxylase	non	non	non	non	essential	yes
**SMU_713**	ftsW	cell division protein	essential	essential	essential	essential	-	yes
**SMU_714**	tuf	translation elongation factor Tu	essential	non	essential	essential	-	yes
**SMU_715**	tpiA	triosephosphate isomerase	essential	essential	non	essential	-	yes

**SMU_716**	murN	peptidoglycan branched peptide synthesis protein, alanine adding enzyme; methicillin resistance factor	non	non	essential	non	-	yes
**SMU_738**	-	hypothetical protein	non	non	non	non	non	no
**SMU_754**	-	HPr (serine) kinase/phosphatase	essential	non	essential	non	-	yes
**SMU_766**	-	transposase	non	non	non	non	non	no
**SMU_773c**	lysS	lysyl-tRNA synthetase	essential	essential	essential	essential	-	yes
**SMU_777**	aroD	3-dehydroquinate dehydratase	non	non	non	non	essential	yes
**SMU_778**	aroE	shikimate 5-dehydrogenase	non	non	non	non	essential	yes
**SMU_779**	aroB	3-dehydroquinate synthase	non	non	non	non	essential	yes
**SMU_780**	aroC	chorismate synthase	non	non	non	non	essential	yes
**SMU_784**	aroA	3-phosphoshikimate 1-carboxyvinyltransferase (5-enolpyruvylshikimate-3-phosphate synthase)	non	non	non	non	essential	yes
**SMU_785**	aroK	shikimate kinase	non	non	non	non	essential	yes
**SMU_801**	obg	GTP-binding protein, GTP1/Obg family	essential	essential	essential	essential	-	yes
**SMU_81**	grpE	co-chaperone protein GrpE	essential	non	essential	non	-	yes
**SMU_821**	dnaG	DNA primase	essential	essential	essential	essential	-	yes
**SMU_822**	rpoD	RNA polymerase sigma-70 factor	essential	essential	essential	essential	-	yes
**SMU_824**	rmlD	dTDP-4-keto-L-rhamnose reductase	essential	non	essential	non	-	yes
**SMU_827**	rgpC	polysaccharide ABC transporter membrane-spanning protein	non	non	essential	non	-	yes
**SMU_830**	rgpF	polysaccharide biosynthesis protein	non	non	essential	non	-	yes

**SMU_846**	rplU	Ribosomal protein L21-within SMU_847c on opposite strand	essential	non	non	non	-	yes
**SMU_849**	rpmA	50S ribosomal protein L27	essential	non	non	essential	-	yes
**SMU_868**	trmD	tRNA (guanine-N1)-methyltransferase	non	essential	essential	essential	-	no
**SMU_939**	fni	isopentenyl-diphosphate delta-isomerase	non	non	essential	essential	-	yes
**SMU_942**	mvaA	3-hydroxy-3-methylglutaryl-CoA reductase	essential	essential	non	essential	-	no
**SMU_943c**	hmcM	hydroxymethylglutaryl-CoA synthase	essential	essential	essential	essential	-	no
**SMU_947**	dfrA	dihydrofolate reductase	essential	essential	essential	essential	-	no
**SMU_950**	era	GTP-binding protein	essential	essential	essential	essential	-	no
**SMU_957**	rplJ	50S ribosomal protein L10	essential	non	essential	non	-	no
**SMU_959c**	-	hypothetical protein	non	non	non	non	non	no
**SMU_960**	rpl	50S ribosomal protein L7/L12	essential	non	non	essential	-	no
**SMU_965**	hom	homoserine dehydrogenase	non	non	non	non	essential	no
**SMU_966**	thrB	homoserine kinase	non	non	non	non	essential	no
**SMU_97**	pyrG	CTP synthetase	essential	non	non	non	-	yes
**SMU_989**	asd	aspartate-semialdehyde dehydrogenase	non	non	non	non	essential	no
**SMU_99**	fbaA	fructose-bisphosphate aldolase	essential	essential	essential	essential	-	yes
**SMU_993**	ylqL	GTP-binding protein	non	non	non	non	essential	no

[a]Essential genes not conserved among the streptococci strains were compared against the rest of the essential genomes stored in the DEG

[b]A gene is defined as being in the accessory genome if only 1 of the 67 sequenced strains lacks the gene
