# Supplementary material for: Genomewide Identification of Essential Genes and Fitness Determinants of Streptococcus mutans UA159
Source: mSphere. 2018 Feb 7;3(1):e00031-18. doi: 10.1128/mSphere.00031-18 (PMC5806208; doi:10.1128/mSphere.00031-18)
Supplement: TABLE S4 [file sph001182474st4.pdf]

Table S4

Strain or plasmid	Genotype or description	Reference
Streptococcus mutans		
UA159	Wild-type	ATCC 700610
Δ*clpP*	clpP::km	(1)
Δ*sufC*	sufC::km	(2)
Δ*rpmH*	SMu.340::km	(2)
Δ*pppL*	pppL::km	This work
Δ*pknB*	pknB::km	(2)
Δ*divIB*	divIB::km	(2)
Δ*ltaS*	Smu.831::km	(2)
Δ*rgpI*	Smu.833::km	(2)
Δ852	SMu.852::km	This work
Δ1542	Smu.1542c::km	(2)
Δ*dltA*	dltA::km	(2)
Δ*scrB*	scrB::km	(3)
Δ*vicK*	vicK::km	(4)
Δ*sodA*	sodA::km	This work
Plasmids		
pALH124	Non-polar kanamycin cassette	(5)
pMalC9	Plasmid that expresses the mariner transposase, MarC9, fused to an N-terminal maltose binding protein to allow purification	(6)
pMagellan6	Magellan6 transposon is carried on this plasmid, including a spectinomycin resistance gene (*aad9*)	(7)

REFERENCES

1. Lemos JAC, Burne RA. 2002. Regulation and Physiological Significance of ClpC and ClpP in *Streptococcus mutans*. J Bacteriol 184:6357–66.
2. Shields RC, O'Brien G, Maricic N, Kesterson A, Grace M, Hagen SJ, Burne RA. 2018. Genome-wide screens reveal new gene products that influence genetic competence in *Streptococcus mutans*. J Bacteriol 200:16 e00508-17.
3. Zeng L, Burne RA. 2013. Comprehensive mutational analysis of sucrose-metabolizing pathways in *Streptococcus mutans* reveals novel roles for the sucrose phosphotransferase system permease. J Bacteriol 195:833–43.
4. Ahn S-J, Burne RA. 2007. Effects of oxygen on biofilm formation and the AtlA autolysin of *Streptococcus mutans*. J Bacteriol 189:6293–302.
5. Ahn S-J, Burne RA. 2006. The *atlA* operon of *Streptococcus mutans*: role in autolysin maturation and cell surface biogenesis. J Bacteriol 188:6877–88.
6. Lampe DJ, Akerley BJ, Rubin EJ, Mekalanos JJ, Robertson HM. 1999. Hyperactive transposase mutants of the Himar1 mariner transposon. Proc Natl Acad Sci U S A 96:11428–33.
7. van Opijnen T, Bodi KL, Camilli A. 2009. Tn-seq: high-throughput parallel sequencing for fitness and genetic interaction studies in microorganisms. Nat Methods 6:767–72.
