# Supplementary material for: Genomewide Identification of Essential Genes and Fitness Determinants of Streptococcus mutans UA159
Source: mSphere. 2018 Feb 7;3(1):e00031-18. doi: 10.1128/mSphere.00031-18 (PMC5806208; doi:10.1128/mSphere.00031-18)
Supplement: TABLE S5 [file sph001182474st5.pdf]

Table S5

Primer	Sequence (5' to 3')[a,b,c]	Reference
**Tn-seq**		
TnseqBarcode1A	TTCCCTACACGACGCTCTTCCGATCT TATAGCCT NN	This work
TnseqBarcode1B	p-AGGCTATA AGATCGGAAGAGCGTCGTGTAGGGAAAGAG-p	This work
TnseqBarcode2A	TTCCCTACACGACGCTCTTCCGATCT ATAGAGGC NN	This work
TnseqBarcode2B	p-GCCTCTAT AGATCGGAAGAGCGTCGTGTAGGGAAAGAG-p	This work
TnseqBarcode3A	TTCCCTACACGACGCTCTTCCGATCT CCTATCCT NN	This work
TnseqBarcode3B	p-AGGATAGG AGATCGGAAGAGCGTCGTGTAGGGAAAGAG-p	This work
TnseqBarcode4A	TTCCCTACACGACGCTCTTCCGATCT GGCTCTGA NN	This work
TnseqBarcode4B	p-TCAGAGCC AGATCGGAAGAGCGTCGTGTAGGGAAAGAG-p	This work
TnseqBarcode5A	TTCCCTACACGACGCTCTTCCGATCT AGGCGAAG NN	This work
TnseqBarcode5B	p-CTTCGCCT AGATCGGAAGAGCGTCGTGTAGGGAAAGAG-p	This work
TnseqBarcode6A	TTCCCTACACGACGCTCTTCCGATCT TAATCTTA NN	This work
TnseqBarcode6B	p-TAAGATTA AGATCGGAAGAGCGTCGTGTAGGGAAAGAG-p	This work
TnseqBarcode7A	TTCCCTACACGACGCTCTTCCGATCT CAGGACGT NN	This work
TnseqBarcode7B	p-ACGTCCTG AGATCGGAAGAGCGTCGTGTAGGGAAAGAG-p	This work
TnseqBarcode8A	TTCCCTACACGACGCTCTTCCGATCT GTACTGAC NN	This work

TnseqBarcode8B	p-GTCAGTAC AGATCGGAAGAGCGTCGTGTAGGGAAAGAG-p	This work
P1_M6_MmeI	CAA GCA GAA GAC GGC ATA CGA AGA CCG GGG ACT TAT CAT CCA ACC TGT	(1)
ADPT-Tnseq-PCRPrimer	AAT GAT ACG GCG ACC ACC GAG ATC TAC ACT CTT TCC CTA CAC GAC GCT CTT CCG ATCT	(1)
Genomic DNA Sequencing Primer	ACA CTC TTT CCC TAC ACG ACG CTC TTC CGA TCT	(1)
**Mutagenesis**		
Streptococcus mutans		
SMu.483cA	AAGCCGCAAAAATTAGATGC	This work
SMu.483cB	ATGCGGATCCCATGCCATCTGCCAATACAA	This work
SMu.483cC	ATGCGGATCCTGAAAAAGCCAAGCATTTGA	This work
SMu.483cD	CCGTTCCATCTTTGGTCAGT	This work
SMu.483cE	ATTTGACGACAGGCCAGATT	This work
SMu.483cF	GCGCCTGTTCAGGAGATAAG	This work
SMu.852A	CGAGGCAAAGAGCAAGATTC	This work
SMu.852B	CGATGGATCCCAAACTGGGCTGACTGACAA	This work
SMu.852C	CGATGGATCCTTATCCACGTGTGCTCTCAAA	This work
SMu.852D	ATCAGCCAAAGCCTTATCCA	This work
SMu.852E	ATTGGCTAAGTGCCAGCAAG	This work
SMu.852F	CAGCGACATCCTCATCTTCA	This work
SMu.689A	TGCGTCTTCAGGGAGAAGAT	This work
SMu.689B	ATGCGGATCCAGGGTCATCGTTTCAGCATC	This work
SMu.689C	ATGCGGATCCTTGGAACACTGTTGCTCGTC	This work
SMu.689D	GGACCCTTAGCAGAATAACAAGA	This work
SMu.689E	TCAGTGAGGCTATTCCCAAGA	This work
SMu.689F	GGTTCACTTGATTCGGGAGT	This work

[a]N indicates a degenerate base that could be either A, C, G, or T

[b]p indicates that the base adjacent has a phosphorylation modification

[c]underlined sequences indicate restriction enzyme recognition sites

REFERENCE

1. van Opijnen T, Camilli A. 2010. Genome-wide fitness and genetic interactions determined by Tn-seq, a high-throughput massively parallel sequencing method for microorganisms. Curr Protoc Microbiol Chapter 1:Unit1E.3.
